# Supplementary material for: Orthopedic Treatment for Class II Malocclusion with Functional Appliances and Its Effect on Upper Airways: A Systematic Review with Meta-Analysis
Source: J Clin Med. 2020 Nov 25;9(12):3806. doi: 10.3390/jcm9123806 (PMC7759817; doi:10.3390/jcm9123806)

SUPPLEMENTARY MATERIAL

Orthopedic Treatment for Class II Malocclusion with Functional Appliances and Its Effect on Upper Airways: A Systematic Review with Meta-Analysis

Darius Bidjan, Rahel Sallmann, Theodore Eliades and Spyridon N. Papageorgiou *

Clinic of Orthodontics and Pediatric Dentistry, Center of Dental Medicine, University of Zurich, 8032 Zurich, Switzerland; darius.bidjan@bluewin.ch (D.B.); rahel.sallmann@hotmail.com (R.S.); theodore.eliades@zzm.uzh.ch (T.E.)

* Correspondence: snpapage@gmail.com

Table S1. Literature searches with resulting hits (last search date: October 20th, 2020)

Nr	Database	Search strategy	Limits	Hits
1	PubMed	(orthodon* OR orthop* OR "Class II" OR malocclusion OR (mandib* AND retrogn*)) AND ("functional appliance" OR "functional appliances" OR "bite-jumping" OR "mandibular advancement" OR Activator OR Biobloc OR Bionator OR Dynamax OR Eureka OR Forsus OR Frankel OR Fränkel OR Harvold OR Herbst OR "Jasper Jumper" OR Klammt OR "Mandibular Anterior Repositioning Appliance" OR MARA OR MiniScope OR Monobloc OR "Mono-bloc" OR PowerScope OR "R Appliance" OR Sander OR "Schwarz appliance" OR "Schwarz double platte" OR Twinblock OR "Twin-Block" OR "Twin-Blocks" OR Xbow) AND (airway* OR breath* OR "Apnea Hypopnea index" OR "Eppworth Sleepiness Scale")	-	864
2	Embase	Same as PubMed	-	130
3	CDSR	Same as PubMed	-	9
4	CENTRAL	Same as PubMed	-	164
5	DARE	Same as PubMed	-	0
6	Scopus	(TITLE-ABS-KEY ((orthodon* OR orthop* OR "Class II" OR malocclusion OR (mandib* AND retrogn*))) AND TITLE-ABS-KEY (("functional appliance" OR "functional appliances" OR "bite-jumping" OR "mandibular advancement" OR Activator OR Biobloc OR Bionator OR Dynamax OR Eureka OR Forsus OR Frankel OR Fränkel OR Harvold OR Herbst OR "Jasper Jumper" OR Klammt OR "Mandibular Anterior Repositioning Appliance" OR MARA OR MiniScope OR Monobloc OR "Mono-bloc" OR PowerScope OR "R Appliance" OR Sander OR "Schwarz appliance" OR "Schwarz double platte" OR Twinblock OR "Twin-Block" OR "Twin-Blocks" OR Xbow)) AND TITLE-ABS-KEY ((airway* OR breath* OR "Apnea Hypopnea index" OR "Eppworth Sleepiness Scale"))) AND (LIMIT-TO (SUBJAREA , "DENT"))	Dentistry	279
7	Web of Science	Same as PubMed	DENTISTRY ORAL SURGERY MEDICINE	559
8	Virtual Health Library	Same as PubMed	-	90
9	ClinicalTrials.gov	-	-	-
**TOTAL**				**2095**

CDSR, Cochrane Database of Systematic Reviews; CENTRAL, Cochrane Central Register of Controlled Trials; DARE, Cochrane Database of Abstracts of Reviews of Effects

Table S2. List of studies identified from the literature search and their inclusion / exclusion status, with reasons.

Nr	Paper	Status
1	{ACTRN} A Randomized, Double-Blind, Placebo-Controlled, Parallel-Group, Study to Assess The Effects of Intravenous BG9928 on Body Weight in Subjects with Acute Decompensated Heart Failure and Renal Insufficiency. http://wwwwhoint/trialsearch/Trial2aspx?TrialID=ACTRN12608000607370. 2008.	Excluded by title
2	{ACTRN} Feasibility and potential benefits of high-intensity interval cycling for knee osteoarthritic patients: a randomised control feasibility trial? http://wwwwhoint/trialsearch/Trial2aspx?TrialID=ACTRN12616000273482. 2016.	Excluded by title
3	{ACTRN} Mouthguard and positional trainer(vibrating collar) in supine sleep apnoea. http://wwwwhoint/trialsearch/Trial2aspx?TrialID=ACTRN12618000207213. 2018.	Excluded by title
4	{CTRI} Comparison of efficacy of 2 different mouthrinses. http://wwwwhoint/trialsearch/Trial2aspx?TrialID=CTRI/2018/01/011237. 2018.	Excluded by title
5	{CTRI} Home based exercises in pulmonary hypertension. http://wwwwhoint/trialsearch/Trial2aspx?TrialID=CTRI/2010/091/001492. 2011.	Excluded by title
6	{DRKS} a randomized controlled trial investigating the efficacy and benefit of the health program "initiative.rücken" in members of a private health insurance with persistent unspecific low back pain. http://wwwwhoint/trialsearch/Trial2aspx?TrialID=DRKS00015463. 2018.	Excluded by title
7	{DRKS} Influence of non-surgical and surgical orthopedic treatment on stress and depression levels, sympathetic and parasympathetic cardiovascular modulation and baroreflex sensitivity in patients with osteoarthritis. http://wwwwhoint/trialsearch/Trial2aspx?TrialID=DRKS00004602. 2012.	Excluded by title
8	{DRKS} The spinal anesthesia with hyperbaric Lokalanästhestikum performed in the lateral position, compared to general anesthesia in outpatient anesthesia, in terms of effectiveness, process times, hemodynamic stability, perioperative complications, and postoperative pain. http://wwwwhoint/trialsearch/Trial2aspx?TrialID=DRKS00004906. 2013.	Excluded by title
9	{Euctr FR} A double-blind, randomized, placebo-controlled, study evaluating the safety and activity of four escalating single doses of AVE0657 in congestive heart failure patients presenting as Cheyne-Stokes Breathing Syndrome. http://wwwwhoint/trialsearch/Trial2aspx?TrialID=EUCTR2007-002172-34-FR. 2007.	Excluded by title
10	{Euctr IT} Breathing disorders in heart failure: high altitude hypoxia as a model to define diagnostic tools, therapeutic strategies and gender related differences. - ND. http://wwwwhoint/trialsearch/Trial2aspx?TrialID=EUCTR2010-019986-27-IT. 2010.	Excluded by title
11	{Irct138810223051N} Comparison of the effect of intratracheal and intravenous lidocaine on bucking, cough, and emergence time at the end of anesthesia. http://wwwwhoint/trialsearch/Trial2aspx?TrialID=IRCT138810223051N1. 2010.	Excluded by title
12	{Irct2013022712123N} Cost-effectiveness analysis of intravenous propofol anesthesia versus inhalational Isoflurane anesthesia in patients undergo open surgery at Shahroud's Imam Hossein hospital. http://wwwwhoint/trialsearch/Trial2aspx?TrialID=IRCT2013022712123N2. 2013.	Excluded by title
13	{Irct2016081729405N} Effects of 8 weeks of combined aerobic and resistive exercise on fat percentage, quality of life and fatigue in patients with pulmonary sarcoidosis. http://wwwwhoint/trialsearch/Trial2aspx?TrialID=IRCT2016081729405N1. 2016.	Excluded by title
14	{ISRCTN} Therapy-efficacy of a new mode of Automatic Servo-Ventilation in patients with complicated breathing patterns during sleep. http://wwwwhoint/trialsearch/Trial2aspx?TrialID=ISRCTN62161274. 2010.	Excluded by title
15	{KCT} Effects of Core Stabilization Exercise and Hip-targeted Muscles Stretching and Strengthening Exercise on Physical Function and Activity in Patients with Non-specific Low Back Pain. http://wwwwhoint/trialsearch/Trial2aspx?TrialID=KCT0004140. 2019.	Excluded by title
16	{NCT} A Pilot Study in Severe Patients With Takayasu Arteritis. https://clinicaltrialsgov/show/NCT04300686. 2020.	Excluded by title
17	{NCT} A Trial to Study the Effects of Pulmonary Rehabilitation Program on Exercise Capacity and Quality of Life in Patients With Severe Form of Chronic Obstructive Pulmonary Disease (COPD). Https://clinicaltrialsgov/show/nct02512549 [Internet]. 2015. Available from: https://www.cochranelibrary.com/central/doi/10.1002/central/CN-01491264/full.	Excluded by title
18	{NCT} Biofeedback Training, Efficacy Evaluation of Fibromyalgia Treatment, a Pilot Study. https://clinicaltrialsgov/show/NCT04121832. 2019.	Excluded by title
19	{NCT} Effect of Chronic Changes in Heart Rate on Congestive Heart Failure. Https://clinicaltrialsgov/show/nct00389649 [Internet]. 2006. Available from: https://www.cochranelibrary.com/central/doi/10.1002/central/CN-01513198/full.	Excluded by title
20	{NCT} Effect of Single Rinsing With Three Different Types of Mouthwash on VSC Levels in Morning Breath. https://clinicaltrialsgov/show/NCT04279106. 2020.	Excluded by title
21	{NCT} Effect of the Nursing Process on the Quality of Life of the Patient With Definitive Pacemaker Implantation. https://clinicaltrialsgov/show/NCT03999671. 2019.	Excluded by title
22	{NCT} Effectiveness of Chest Wall Mobilization Program in Improving Respiratory Function for Patients With COPD. https://clinicaltrialsgov/show/NCT04122365. 2019.	Excluded by title
23	{NCT} Effects of Two Different Dry-Needling Techniques for Low Back Pain. https://clinicaltrialsgov/show/NCT03970486. 2019.	Excluded by title
24	{NCT} Efficiency of Methotrexate and Tofacitinib in Mild and Moderate Patients. https://clinicaltrialsgov/show/NCT04299971. 2020.	Excluded by title
25	{NCT} Evaluation of Combined Action Between Natrecor and Furosemide on Kidney and Neurohormone Responses in Chronic Heart Failure: a Phase-IV study704.351 / DSS. Https://clinicaltrialsgov/show/nct00652652 [Internet]. 2008. Available from: https://www.cochranelibrary.com/central/doi/10.1002/central/CN-01518124/full.	Excluded by title
26	{NCT} Evaluation of the Effect of Different Rapid Maxillary Expansion Appliances on Airway by Acoustic Rhinometry. https://clinicaltrialsgov/show/NCT04529057. 2020.	Excluded by title
27	{NCT} Feasibility & Effect of a Tele-rehabilitation Program in Idiopathic Pulmonary Fibrosis (IPF). Https://clinicaltrialsgov/show/nct03548181 [Internet]. 2018. Available from: https://www.cochranelibrary.com/central/doi/10.1002/central/CN-01660104/full.	Excluded by title
28	{NCT} Ketorolac for Analgesia followiNG Autologous Breast RecOnstructiOn. Https://clinicaltrialsgov/show/nct03007381 [Internet]. 2017. Available from: https://www.cochranelibrary.com/central/doi/10.1002/central/CN-01560962/full.	Excluded by title
29	{NCT} Nalbuphine Versus Fentanyl As Additives To Bupivacaine In Spinal Anaesthesia For Internal FixationI Of Tibia. Https://clinicaltrialsgov/show/nct03535792 [Internet]. 2018. Available from: https://www.cochranelibrary.com/central/doi/10.1002/central/CN-01659816/full.	Excluded by title
30	{NCT} Patient Education and Basic Body Awareness Therapy in Hip Osteoarthritis: a Randomized Controlled Trial. Https://clinicaltrialsgov/show/nct02884531 [Internet]. 2016. Available from: https://www.cochranelibrary.com/central/doi/10.1002/central/CN-01520517/full.	Excluded by title
31	{NCT} Pelvic Health and Physical Therapy to Improve Lives of Prostate Cancer Patients Undergoing Prostatectomy. https://clinicaltrialsgov/show/NCT04027270. 2019.	Excluded by title
32	{NCT} Polygraphic Evaluation of the Effects of Different Rapid Maxillary Expansion Appliances on Sleep Quality. https://clinicaltrialsgov/show/NCT04529213. 2020.	Excluded by title
33	{NCT} Pulmonary Rehabilitation Program and PROactive Tool. https://clinicaltrialsgov/show/NCT02437994. 2015.	Excluded by title
34	{NCT} SheppHeartCABG - Phase One Rehabilitation After Coronary Artery Bypass Grafting. https://clinicaltrialsgov/show/NCT02290262. 2014.	Excluded by title
35	{NCT} Surgical Adhesive Drapes for Prevention and Healing of Wound Infections After Total Hip Arthroplasty. Https://clinicaltrialsgov/show/nct02707302 [Internet]. 2016. Available from: https://www.cochranelibrary.com/central/doi/10.1002/central/CN-01556492/full.	Excluded by title
36	{NCT} The Effect of Diaphragmatic Breathing Exercise on Pain, Anxiety, and Depression. https://clinicaltrialsgov/show/NCT04225169. 2020.	Excluded by title
37	{NCT} The Effect of Kinesio Taping on Pulmonary Function and Functional Capacity in Patients With Chronic Heart Failure. Https://clinicaltrialsgov/show/nct03597646 [Internet]. 2018. Available from: https://www.cochranelibrary.com/central/doi/10.1002/central/CN-01661386/full.	Excluded by title
38	{NCT} The Effect of N-Acetylcystein on Quality of Life and Air Trapping During Rest and After Exercise. https://clinicaltrialsgov/show/NCT00476736. 2007.	Excluded by title

39	{NCT} The Goal of This Study is to Evaluate the Effect of Ultrasound Guided Erector Spinae Plane Block on Postoperative Pain After Major Abdominal Surgeries. https://clinicaltrialsgov/show/NCT04382209. 2020.	Excluded by title
40	{NCT} The Use of Fibrin Sealant to Reduce Post Operative Pain in Cleft Palate Surgery. Https://clinicaltrialsgov/show/nct02953145 [Internet]. 2016. Available from: https://www.cochranelibrary.com/central/doi/10.1002/central/CN-01559734/full.	Excluded by title
41	{NCT} Treatment of Predominant Central Sleep Apnoea by Adaptive Servo Ventilation in Patients With Heart Failure. https://clinicaltrialsgov/show/NCT00733343. 2008.	Excluded by title
42	Abi-Ramia LBP. Avaliação tridimensional da via aérea superior de pacientes com apneia obstrutiva do sono que utilizaram aparelho de avanço mandibular. 2009:93-.	Excluded by title
43	Achilleos S, Krogstad O, Lyberg T. Surgical mandibular advancement and changes in uvuloglossopharyngeal morphology and head posture: a short- and long-term cephalometric study in males. Eur J Orthod. 2000;22(4):367-81.	Excluded by title
44	Agarwal SS, Jayan B, Kumar S. Therapeutic efficacy of a hybrid mandibular advancement device in the management of obstructive sleep apnea assessed with acoustic reflection technique. Indian J Dent Res. 2015;26(1):86-9.	Excluded by title
45	Ahn HW, Cho IS, Cho KC, Choi JY, Chung JW, Baek SH. Surgical treatment modality for facial esthetics in an obstructive sleep apnea patient with protrusive upper Hp and acute nasolabial angle. Angle Orthodontist. 2013;83(2):355-63.	Excluded by title
46	Ahn HW, Lee BS, Kim SW, Kim SJ. Stability of Modified Maxillomandibular Advancement Surgery in a Patient With Preadolescent Refractory Obstructive Sleep Apnea. J Oral Maxillofac Surg. 2015;73(9):1827-41.	Excluded by title
47	Ajiboye OA, Anigbogu CN, Ajuluchukwu JN, Jaja SI. Exercise training improves functional walking capacity and activity level of Nigerians with chronic biventricular heart failure. Hong kong physiotherapy journal. 2015;33(1):42-9.	Excluded by title
48	Akin M, Ucar F, Chousein C, Sari Z. Effects of chincup or facemask therapies on the orofacial airway and hyoid position in Class III subjects. Fortschritte der kieferorthopadie [journal of orofacial orthopedics] [Internet]. 2015; 76(6):[520-30 pp.]. Available from: https://www.cochranelibrary.com/central/doi/10.1002/central/CN-01366664/full.	Excluded by title
49	Albaladejo A, Montero J, López-Valverde A, Gómez de Diego R. Empleo del twin block en el paciente infantil con clase II esquelética y labio superior evertido. Ortod esp (Ed impr). 2010;50(2):419-25.	Excluded by title
50	Albaladejo A, Montero J, López-Valverde A, Gómez de Diego R. Opciones de tratamiento. Así fue tratado: Empleo del twin block en el paciente infantil con clase II esquelética y labio superior evertido. Ortod esp (Ed impr). 2010;50(2):428-35.	Excluded by title
51	Alcalde LFA. Avaliação das alterações nas vias aéreas superiores através de tomografia computadorizada Cone-Beam em pacientes submetidos à cirurgia ortognática de avanço bimaxilar. 2017:77-.	Excluded by title
52	Amini M, Heravi F, Zandi B, Eslami S, Mohajerzadeh M, Rohani M. The effect of mandibular advancement device on physiologic parameters and volumetric MRI in mild to moderate obstructive sleep apnea-a randomized controlled trial. Sleep Medicine. 2017;40:e14-e5.	Excluded by title
53	Andrade LLd, Rodrigues J. Tratamento da síndrome do respirador bucal com mordida aberta através da aparatologia ortopédica funcional. J bras ortodontia ortop maxilar. 1996;1(2):3-13.	Excluded by title
54	Aoki A, Prahl-Andersen B. Mandibular distraction osteogenesis for treatment of extreme mandibular hypoplasia. Am J Orthod Dentofacial Orthop. 2007;132(6):848-55.	Excluded by title
55	Aragão W. Tratamento da mordida aberta anterior, com disto-relação basal com o regulador de função Aragão V (RFA V). J bras ortodontia ortop maxilar. 1996;1(4):23-31.	Excluded by title
56	Aragão W. Tratamento precoce da classe III com o Regulador de Função Aragão III. J bras ortodon ortop facial. 1998;3(18):16-22.	Excluded by title
57	Argandoña J, Marino A, Quiñones P, Cortez C. The changes in the upper airways area following orthognathic surgery. A comparative study. International Journal of Oral and Maxillofacial Surgery. 2011;40(10):1083.	Excluded by title
58	Arisaka T, Ito C, Sato K, Tonogi M, Yamane GY, Nakajima T. Examination of changes in the pharyngeal airway space under anterior traction of the mandible: Influence of detachment of the periosteum during orthognathic surgery. Journal of Oral and Maxillofacial Surgery, Medicine, and Pathology. 2014;26(4):540-4.	Excluded by title
59	Aronovich S. Skeletal reconstruction of craniofacial deformities with alloplastic total joint components and orthognathic surgery. Cleft Palate-Craniofacial Journal. 2016;53(4):e101-e2.	Excluded by title
60	Arx JDv, Merino Arends M, Echarri P, Carrasco A. Tabla de diagnóstico y tratamiento Multifunction System “MFS”, herramienta básica de la estimuloterapia programada. Dentum (Barc). 2009;9(3):119-25.	Excluded by title
61	Aude Celis M. La terapéutica de ortopedia dentofacial y su efecto en la función pulmonar. Rev Círc Argent Odontol. 1999;28(186):24-31.	Excluded by title
62	Awad M, Gouveia C, Zaghi S, Camacho M, Liu SYC. Changing practice: Trends in skeletal surgery for obstructive sleep apnea. Journal of Cranio-Maxillofacial Surgery. 2019;47(8):1185-9.	Excluded by title
63	Azevedo JCM, Cavalcanti AH, Muxfeldt ES. Blood pressure variation on cardiopulmonary test after continuous positive airway pressure therapy in resistant hypertensive patients with obstructive sleep apnea. Sleep science. 2019;12:27-8.	Excluded by title
64	Azevedo WRS, Feitoza CC, Vargas CS, Jr., Pizzol KEDC. Changes in head posture, hyoid bone position and airway dimensions after orthognatic surgery. Brazilian Journal of Oral Sciences. 2018;17.	Excluded by title
65	Baccetti T, Franchi L, Mucedero M, Cozza P. Treatment and post-treatment effects of facemask therapy on the sagittal pharyngeal dimensions in Class III subjects. Eur J Orthod. 2010;32(3):346-50.	Excluded by title
66	Balaji SM. Bilateral pediatric mandibular distraction for micrognathia with temporomandibular joint ankylosis and sleep apnea. Indian J Dent Res. 2017;28(5):588-91.	Excluded by title
67	Bamagoos AA, Cistulli PA, Sutherland K, Madronio M, Eckert DJ, Hess L, et al. Polysomnographic Endotyping to Select Patients with Obstructive Sleep Apnea for Oral Appliances. Ann Am Thorac Soc. 2019;16(11):1422-31.	Excluded by title
68	Bangiyev JN, Traboulsi H, Abdulhamid I, Rozzelle A, Thottam PJ. Sleep architecture in Pierre-Robin sequence: The effect of mandibular distraction osteogenesis. Int J Pediatr Otorhinolaryngol. 2016;89:72-5.	Excluded by title
69	Barth FA, Fritscher GG, Almeida-Pedrin RRd, Ladewig VdM, Casteluci CEVF, Conti ACdCF. Protocolo de Tratamento Orto-Cirúrgico da Má Oclusão de Classe II em paciente com Sindrome da Apnéia e/ou Hipoapnéia Obstrutiva do sono: relato de caso. Rev Salusvita (Online). 2019;38(3):693-711.	Excluded by title
70	Brunetto DP, Velasco L, Koerich L, Araujo MT. Prediction of 3-dimensional pharyngeal airway changes after orthognathic surgery: a preliminary study. Am J Orthod Dentofacial Orthop. 2014;146(3):299-309.	Excluded by title
71	Brunso J, Franco M, Constantinescu T, Barbier L, Santamaría JA, Alvarez J. Custom-Machined Miniplates and Bone-Supported Guides for Orthognathic Surgery: A New Surgical Procedure. Journal of Oral and Maxillofacial Surgery. 2016;74(5):1061.e1-.e12.	Excluded by title
72	Buijze GA, De Jong HMY, Kox M, van de Sande MG, Van Schaardenburg D, Van Vugt RM, et al. An add-on training program involving breathing exercises, cold exposure, and meditation attenuates inflammation and disease activity in axial spondyloarthritis - A proof of concept trial. PLoS One. 2019;14(12):e0225749.	Excluded by title
73	Burstein FD. Resorbable distraction of the midface and orbits and frontal bone: Indications and operative technique. Operative Techniques in Otolaryngology - Head and Neck Surgery. 2005;16(4):260-3.	Excluded by title
74	Butler G, Naughton M, Rahman M, Bradley T, Floras J. Continuous positive airway pressure increases heart rate variability in congestive heart failure. Journal of the american college of cardiology [Internet]. 1995; 25(3):[672-9 pp.]. Available from: https://www.cochranelibrary.com/central/doi/10.1002/central/CN-00110693/full.	Excluded by title
75	Cao MT, Sternbach JM, Guilleminault C. Continuous positive airway pressure therapy in obstuctive sleep apnea: benefits and alternatives. Expert Rev Respir Med. 2017;11(4):259-72.	Excluded by title
76	Capan E, Ersu R, Kiyan E, Yener HM, Arman A, Kilicoglu H. Monoblock appliance for treatment of children with sleep disordered breathing. American Journal of Respiratory and Critical Care Medicine. 2015;191.	Excluded by title
77	Caplan R, Ready L, Oden R, Matsen F, Nessly M, Olsson G. Transdermal fentanyl for postoperative pain management. A double-blind placebo study. Jama [Internet]. 1989; 261(7):[1036-9 pp.]. Available from: https://www.cochranelibrary.com/central/doi/10.1002/central/CN-00057859/full.	Excluded by title
78	Caravadossi A. El individuo indiviso a través de un caso clínico. Rev Asoc Argent Ortop Funcional Maxilares. 2004;34(1):21-4.	Excluded by title

79	Carlstedt K, Henningsson G, Dahllöf G. A four-year longitudinal study of palatal plate therapy in children with Down syndrome: Effects on oral motor function, articulation and communication preferences. Acta Odontologica Scandinavica. 2003;61(1):39-46.	Excluded by title
80	Carra MC, Lavigne G, Rompré P. Sleep bruxismand headache in adolescents. Sleep Medicine. 2011;12:S21.	Excluded by title
81	Cerruto C, Di VL, Doldo T, Giovannetti A, Polimeni A, Goracci C. A computerized photographic method to evaluate changes in head posture and scapular position following rapid palatal expansion: a pilot study. Journal of clinical pediatric dentistry [Internet]. 2012; 37(2):[213-8 pp.]. Available from: https://www.cochranelibrary.com/central/doi/10.1002/central/CN-00858181/full.	Excluded by title
82	Chang KK, Kim KB, McQuilling MW, Movahed R. Fluid structure interaction simulations of the upper airway in obstructive sleep apnea patients before and after maxillomandibular advancement surgery. Am J Orthod Dentofacial Orthop. 2018;153(6):895-904.	Excluded by title
83	Chang MK, Sears C, Huang JC, Miller AJ, Kushner HW, Lee JS. Correlation of Airway Volume With Orthognathic Surgical Movement Using Cone-Beam Computed Tomography. J Oral Maxillofac Surg. 2015;73(12 Suppl):S67-76.	Excluded by title
84	Chen NH, Lin SW, Chuang LP, Cistulli PA, Hsieh MJ, Kao KC, et al. Pharyngeal distensibility during expiration is an independent predictor of the severity of obstructive sleep apnoea. Respirology. 2019;24(6):582-9.	Excluded by title
85	Cho J, Neugarten J, Grewal N, Veler H. Polysomnography to Determine the End Point of Neonatal/Pediatric Mandibular Distraction. Journal of Oral and Maxillofacial Surgery. 2019;77(9):e66-e7.	Excluded by title
86	Chrysohoou C, Pitsavos C, Tsitsinakis G, Aggelis A, Tsiachris D, Vogiatzis J, et al. High intensity, interval exercise improves pulse wave analysis of the aortic pressure waveform in severe left ventricular systolic dysfunction: a phase III randomized clinical trial. European heart journal [Internet]. 2014; 35:[63 p.]. Available from: https://www.cochranelibrary.com/central/doi/10.1002/central/CN-01055271/full.	Excluded by title
87	Chrysohoou C, Pitsavos C, Tsitsinakis G, Vogiatzis J, Tsiachris D, Aggelis A, et al. High intensity, interval exercise improves the quality of life, ventricular diastolic function, ergometric capacity and psychological status of patients with chronic heart failure: a phase III randomi. European heart journal [Internet]. 2014; 35:[62 p.]. Available from: https://www.cochranelibrary.com/central/doi/10.1002/central/CN-01055272/full.	Excluded by title
88	Chrysohoou C, Tsitsinakis G, Vogiatzis I, Cherouveim E, Antoniou C, Tsiantilas A, et al. High intensity, interval exercise improves quality of life of patients with chronic heart failure: a randomized controlled trial. QJM : monthly journal of the association of physicians [Internet]. 2014; 107(1):[25-32 pp.]. Available from: https://www.cochranelibrary.com/central/doi/10.1002/central/CN-01002468/full.	Excluded by title
89	Claudino LV. Avaliação das vias aéreas em pacientes submetidos à cirurgia de avanço mandibular. 2017:86-.	Excluded by title
90	Cleland J, Dargie H, Ball S, Gillen G, Hodsman G, Morton J, et al. Effects of enalapril in heart failure: a double blind study of effects on exercise performance, renal function, hormones, and metabolic state. British heart journal [Internet]. 1985; 54(3):[305-12 pp.]. Available from: https://www.cochranelibrary.com/central/doi/10.1002/central/CN-00039608/full.	Excluded by title
91	Cobo J, Canut JA, Carlos F, Vijande M, Llamas JM. Changes in the upper airway of patients who wear a modified functional appliance to treat obstructive sleep apnea. Int J Adult Orthodon Orthognath Surg. 1995;10(1):53-7.	Excluded by title
92	Coelho-Ferraz MJP, Siqueira VCVd, Nouer DF, Sousa MAd. Avaliação cefalométrica da posição do osso hióide em respiradores bucais - relato de caso clínico. J bras ortodon ortop facial. 2006;11(61):10-20.	Excluded by title
93	Cohen-Levy J, Garcia R, Petelle B, Fleury B. Treatment of the obstructive sleep apnea syndrome in adults by mandibular advancement device: the state of the art. Int Orthod. 2009;7(3):287-304.	Excluded by title
94	Cohen-Levy J, Petelle B, Vieille E, Dumitrache M, Fleury B. Changes in facial profile after maxillomandibular advancement surgery for obstructive sleep apnea syndrome. Int Orthod. 2013;11(1):71-92.	Excluded by title
95	Comotto M, Saghazadeh S, Bagherifard S, Aliakbarian B, Kazemzadeh-Narbat M, Sharifi F, et al. Breathable hydrogel dressings containing natural antioxidants for management of skin disorders. J Biomater Appl. 2019;33(9):1265-76.	Excluded by title
96	Cortes M, Gomez M, Park S, Singh D. A combined approach for upper airway remodeling for skeletal class iii malocclusion with complex OSA. Sleep. 2018;41:A206.	Excluded by title
97	Cortese LG, Palacios S, Oliver L. Trastornos temporomandibulares en niños y adolescentes. Bol Asoc Argent Odontol Niños. 2004;33(2):12-9.	Excluded by title
98	Damiani G, Pigatto PDM, Marzano AV, Rizzi M, Santus P, Radovanovic D, et al. Malar rash is a predictor of subclinical airway inflammation in patients with systemic lupus erythematosus: a pilot study. Clin Rheumatol. 2019;38(9):2541-6.	Excluded by title
99	Dauria D, Marsh JL. Mandibular distraction osteogenesis for Pierre Robin sequence: what percentage of neonates need it? J Craniofac Surg. 2008;19(5):1237-43.	Excluded by title
100	de Sousa Miranda W, Alvares de Castro Rocha V, Lara Dos Santos Marques K, Trindade Neto AI, do Prado CJ, Zanetta-Barbosa D. Three-dimensional evaluation of superior airway space after orthognathic surgery with counterclockwise rotation and advancement of the maxillomandibular complex in Class II patients. Oral Surg Oral Med Oral Pathol Oral Radiol. 2015;120(4):453-8.	Excluded by title
101	Demko BG. The treatment of obstructive sleep apnea with oral devices. J Mass Dent Soc. 2008;57(2):20-3.	Excluded by title
102	Demko BG. Therapeutic options for obstructive sleep apnea. J Mass Dent Soc. 2008;57(2):16-9.	Excluded by title
103	Do Amaral CER, Do Amaral CAR, Ghizoni E. Monobloc fronto-facial advancement with distraction osteogenesis for syndromic craniosynostosis: Comparison between 2 distractors. Child's Nervous System. 2014;30(11):1962.	Excluded by title
104	Do DC, Mu J, Ke X, Sachdeva K, Qin Z, Wan M, et al. miR-511-3p protects against cockroach allergen-induced lung inflammation by antagonizing CCL2. JCI Insight. 2019;4(20).	Excluded by title
105	Do JBA, Bellerive A, Julien AS, Leclerc JE. Cleft Palates and Occlusal Outcomes in Pierre Robin Sequence. Otolaryngol Head Neck Surg. 2019;160(2):246-54.	Excluded by title
106	Do K. The Effects of Mandibular Position on Airway and TMD: A Case Report. Int J Orthod Milwaukee. 2016;27(4):53-7.	Excluded by title
107	Duff H, Oates J, Roden D, Woosley R. The antiarrhythmic activity of meobentine sulfate in man. Journal of cardiovascular pharmacology [Internet]. 1984; 6(4):[650-6 pp.]. Available from: https://www.cochranelibrary.com/central/doi/10.1002/central/CN-00192406/full.	Excluded by title
108	Eckardt F, Engel J, Mann ST, Muller M, Zajonz T, Koerner CM, et al. LMA Protector Airway: first experience with a new second generation laryngeal mask. Minerva Anestesiol. 2019;85(1):45-52.	Excluded by title
109	Eckardt F, Engel J, Mann ST, Müller M, Zajonz T, Koerner CM, et al. LMA Protector™ Airway: first experience with a new second generation laryngeal mask. Minerva Anestesiol. 2019;85(1):45-52.	Excluded by title
110	Efunkoya AA, Bamgbose BO, Adebola RA, Adeoye JB, Akpasa IO. Maxillomandibular distraction osteogenesis. J Craniofac Surg. 2014;25(5):1787-92.	Excluded by title
111	Eichhorn ME. Análisis de un caso clínico. Rev Asoc Argent Ortop Funcional Maxilares. 2007;35(3):21-37.	Excluded by title
112	Engel M, Berger M, Hoffmann J, Kuhle R, Ruckschloss T, Ristow O, et al. Midface correction in patients with Crouzon syndrome is Le Fort III distraction osteogenesis with a rigid external distraction device the gold standard? Journal of Cranio-Maxillofacial Surgery. 2019;47(3):420-30.	Excluded by title
113	Eveloff SE, Rosenberg CL, Carlisle CC, Millman RP. Efficacy of a Herbst mandibular advancement device in obstructive sleep apnea. Am J Respir Crit Care Med. 1994;149(4 Pt 1):905-9.	Excluded by title
114	Faber J, Faber C, Faber AP. Obstructive sleep apnea in adults. Dental Press Journal of Orthodontics. 2019;24(3):99-109.	Excluded by title
115	Farronato G, Salvadori S, Nolet F, Borgonovo A, Esposito L. Pycnodysostosis: Review and case report. Minerva Stomatologica. 2014;63(6):229-37.	Excluded by title
116	Farronato M, Fama A, Lanteri V, Nucci F, Farronato G, Maspero C. Lymphangioma of the tongue associated with open bite: case report. Eur J Paediatr Dent. 2019;20(4):311-4.	Excluded by title
117	Feitoza CC, Azevedo WRdS, Emery JMT, Pereira CV, Vargas Junior CS, Pizzol KEDC. Cirurgia ortognática no tratamento da síndrome da apneia obstrutiva do sono relato de caso. Ortho Sci, Orthod sci pract. 2017;10(38):98-105.	Excluded by title
118	Feitoza CC, Da-Silva MC, Nascimento YL, Leite ES, Pereira CV, Patrocínio LG. Surgical treatment of a pattern i obstructive sleep apnea syndrome individual-clinical case report. Sleep Science. 2017;10(4):168-73.	Excluded by title
119	Feres MAL, Feres R. A Ortodontia no diagnóstico e no tratamento da apneia obstrutiva do sono em crianças. Ortho Sci, Orthod sci pract. 2013;6(23):410-4.	Excluded by title

120	Ferraro FV, Gavin JP, Wainwright T, McConnell A. The effects of 8 weeks of inspiratory muscle training on the balance of healthy older adults: a randomized, double-blind, placebo-controlled study. Physiological reports. 2019;7(9):e14076.	Excluded by title
121	Ferraro FV, Gavin JP, Wainwright TW, McConnell AK. Comparison of balance changes after inspiratory muscle or Otago exercise training. PLoS One. 2020;15(1):e0227379.	Excluded by title
122	Figueroa AA, Polley JW. Management of the severe cleft and syndromic midface hypoplasia. Orthod Craniofac Res. 2007;10(3):167-79.	Excluded by title
123	Fleury B, Cohen-Levy J, Lacassagne L, Buchet I, Geraads A, Pegliasco H, et al. [Treatment of obstructive sleep apnea syndrome using a mandibular advancement device]. Rev Mal Respir. 2010;27 Suppl 3:S146-56.	Excluded by title
124	Foltan R, Hoffmannova J, Pavlikova G, Hanzelka T, Klima K, Horka E, et al. The influence of orthognathic surgery on ventilation during sleep. Int J Oral Maxillofac Surg. 2011;40(2):146-9.	Excluded by title
125	Fong CH, Bebien M, Didierlaurent A, Nebauer R, Hussell T, Broide D, et al. An antiinflammatory role for IKKbeta through the inhibition of "classical" macrophage activation. J Exp Med. 2008;205(6):1269-76.	Excluded by title
126	Franco J, Coppage J, Carstens MH. Mandibular distraction using bone morphogenic protein and rapid distraction in neonates with Pierre Robin syndrome. J Craniofac Surg. 2010;21(4):1158-61.	Excluded by title
127	Fransson A. A mandibular protruding device in obstructive sleep apnea and snoring. Swed Dent J Suppl. 2003(163):1-49.	Excluded by title
128	Fransson AM, Tegelberg A, Svenson BA, Lennartsson B, Isacsson G. Influence of mandibular protruding device on airway passages and dentofacial characteristics in obstructive sleep apnea and snoring. Am J Orthod Dentofacial Orthop. 2002;122(4):371-9.	Excluded by title
129	Furtenbach M. The Significance of the Tongue in Orthodontics and Logopedics. Informationen Aus Orthodontie Und Kieferorthopaedie. 2017;49(1):31-4.	Excluded by title
130	Gajewska BU, Swirski FK, Alvarez D, Ritz SA, Goncharova S, Cundall M, et al. Temporal-spatial analysis of the immune response in a murine model of ovalbumin-induced airways inflammation. Am J Respir Cell Mol Biol. 2001;25(3):326-34.	Excluded by title
131	Gao XM, Zeng XL, Fu MK, Tan QF. [An adjustable appliance in treatment of obstructive sleep apnea-hypopnea syndrome]. Zhonghua Kou Qiang Yi Xue Za Zhi. 2005;40(2):137-40.	Excluded by title
132	Garretto AL. Terapia miofuncional orofacial y su relación con la ortodoncia. Ortodoncia.22-30.	Excluded by title
133	Gasparini G, Azzuni C, Rinaldo FM, Cervelli D, Marianetti TM, Sferrazza A, et al. OSAS treatment with oral appliance: assessment of our experience through the use of a new device. Eur Rev Med Pharmacol Sci. 2013;17(3):385-91.	Excluded by title
134	Gibson D, Laiteerapong N. Pulmonary artery sarcoma: A rare pulmonary artery filling defect. Journal of General Internal Medicine. 2015;30:S442.	Excluded by title
135	Glos M, Penzel T, Schoebel C, Nitzsche GR, Zimmermann S, Rudolph C, et al. Comparison of effects of OSA treatment by MAD and by CPAP on cardiac autonomic function during daytime. Sleep Breath. 2016;20(2):635-46.	Excluded by title
136	Goldfinger M, Chua A, LeFrancois D. Lessons from the heart:The role of echocardiography and thrombolysis in the diagnosis and treatment of pulmonary embolism. Journal of General Internal Medicine. 2013;28:S352.	Excluded by title
137	Goldsmith S. Effect of amlodipine on norepinephrine kinetics and baroreflex function in patients with congestive heart failure. American heart journal [Internet]. 1997; 134(1):[13-9 pp.]. Available from: https://www.cochranelibrary.com/central/doi/10.1002/central/CN-00247399/full.	Excluded by title
138	Goncalves JR, Buschang PH, Goncalves DG, Wolford LM. Postsurgical stability of oropharyngeal airway changes following counter-clockwise maxillo-mandibular advancement surgery. J Oral Maxillofac Surg. 2006;64(5):755-62.	Excluded by title
139	Gonçalves RdC, Raveli DB, Santos-Pinto Ad. Trainer for kids (T4K), um único aparelho para o tratamento de dois problemas verticais: mordida aberta anterior e mordida profunda. Ortodontia. 2011;44(2):174-82.	Excluded by title
140	Gordon F, Wijeyekoon J, Ellis S, Marianayagam T. Nurse-led annual review service for stable inflammatory arthritis. Rheumatology (united kingdom). 2015;54:i98-i9.	Excluded by title
141	Gori S, Alcain J, Vanzulli S, Moreno Ayala MA, Candolfi M, Jancic C, et al. Acetylcholine-treated murine dendritic cells promote inflammatory lung injury. PLoS One. 2019;14(3):e0212911.	Excluded by title
142	Graven LJ, Keltner JG, Fuller K. Problems experienced by heart failure patients the first month after discharge from a heart failure related hospitalization. Journal of cardiac failure. 2017;23(8):S87-.	Excluded by title
143	Gregg JM, Zedalis D, Howard CW, Boyle RP, Prussin AJ. Surgical alternatives for treatment of obstructive sleep apnoea: review and case series. Ann R Australas Coll Dent Surg. 2000;15:181-4.	Excluded by title
144	Grozinger A, Rommelspacher Y, Wirtz DC, Sander K, Pflugmacher R. [Influence of Parkinson's Disease on the Perioperative Course of Patients after Lumbar Fusion Surgery]. Z Orthop Unfall. 2015;153(3):277-81.	Excluded by title
145	Gunaseelan R, Anantanarayanan P, Veerabahu M, Vikraman B. Simultaneous genial distraction and interposition arthroplasty for management of sleep apnoea associated with temporomandibular joint ankylosis. Int J Oral Maxillofac Surg. 2007;36(9):845-8.	Excluded by title
146	Guo J, Gao C, Xin H, Li J, Li B, Wei Z, et al. The application of "upper-body yoga" in elderly patients with acute hip fracture: a prospective, randomized, and single-blind study. J Orthop Surg Res. 2019;14(1):250.	Excluded by title
147	Gupta K, Tiwari V, Gupta P, Pandey M, Singhal A, Shubham G. Clonidine as an adjuvant for ultrasound guided supraclavicular brachial plexus block for upper extremity surgeries under tourniquet: a clinical study. Journal of anaesthesiology, clinical pharmacology [Internet]. 2014; 30(4):[533-7 pp.]. Available from: https://www.cochranelibrary.com/central/doi/10.1002/central/CN-01022051/full.	Excluded by title
148	Gursoy S, Hukki J, Hurmerinta K. Five year follow-up of mandibular distraction osteogenesis on the dentofacial structures of syndromic children. Orthod Craniofac Res. 2008;11(1):57-64.	Excluded by title
149	Gutierrez-Bautista AJ, Morgaz J, Granados MDM, Gomez-Villamandos RJ, Dominguez JM, Fernandez-Sarmiento JA, et al. Evaluation and comparison of postoperative analgesic effects of dexketoprofen and methadone in dogs. Veterinary anaesthesia and analgesia. 2018;45(6):820-30.	Excluded by title
150	Hall A, Ziadi M, Leech J, Chen S, Burwash I, Renaud J, et al. Does short term continuous positive airway pressure alter myocardial energetics and presynaptic sympathetic nerve function in patients with heart failure and obstructive sleep apnea? A randomized study. Canadian journal of cardiology Conference: 65th annual meeting of the canadian cardiovascular society toronto, ON canada Conference start: 20121027 conference end: 20121031 Conference publication: (varpagings) [Internet]. 2012; 28(5 suppl. 1):[S179 p.]. Available from: https://www.cochranelibrary.com/central/doi/10.1002/central/CN-00839950/full.	Excluded by title
151	Hall A, Ziadi M, Leech J, Chen S-Y, Burwash I, Renaud J, et al. Determination of the impact of short term continuous positive airway pressure on myocardial energetics in a randomized study of patients with chronic stable heart failure and obstructive sleep apnea. Circulation [Internet]. 2011; 124(21 Suppl 1):[A14320 p.]. Available from: https://www.cochranelibrary.com/central/doi/10.1002/central/CN-00834096/full.	Excluded by title
152	Han YZ, Jing FY, Xu M, Guo XY. [Anesthesia management of cervical chordoma resection: A case report]. Beijing Da Xue Xue Bao Yi Xue Ban. 2019;51(5):981-3.	Excluded by title
153	Harvold EP, Tomer BS, Vargervik K, Chierici G. Primate experiments on oral respiration. Am J Orthod. 1981;79(4):359-72.	Excluded by title
154	Harvold EP, Vargervik K, Chierici G. Primate experiments on oral sensation and dental malocclusions. Am J Orthod. 1973;63(5):494-508.	Excluded by title
155	Hatipoğlu Ş, Gaş S. Is Information for Surgically Assisted Rapid Palatal Expansion Available on YouTube Reliable? J Oral Maxillofac Surg. 2020;78(6):1017.e1-.e10.	Excluded by title
156	Hiyama S, Ono T, Ishiwata Y, Kuroda T. Changes in mandibular position and upper airway dimension by wearing cervical headgear during sleep. Am J Orthod Dentofacial Orthop. 2001;120(2):160-8.	Excluded by title
157	Hobbins L, Gaoua N, Hunter S, Girard O. Psycho-physiological responses to perceptually-regulated interval runs in hypoxia and normoxia. Physiol Behav. 2019;209:112611.	Excluded by title
158	Hoppenreijs TJ, van der Linden FP, Freihofer HP, van 't Hof MA, Tuinzing DB, Voorsmit RA, et al. Occlusal and functional conditions after surgical correction of anterior open bite deformities. Int J Adult Orthodon Orthognath Surg. 1996;11(1):29-39.	Excluded by title
159	Hopper RA, Ettinger RE, Purnell CA, Dover MS, Pereira AR, Tuncbilek G. Thirty Years Later: What Has Craniofacial Distraction Osteogenesis Surgery Replaced? Plastic and Reconstructive Surgery. 2020;145(6):1073E-88E.	Excluded by title

160	Iatrou I, Theologie-Lygidakis N, Schoinohoriti O. Mandibular distraction osteogenesis for severe airway obstruction in Robin Sequence. Case report. Journal of Cranio-Maxillofacial Surgery. 2010;38(6):431-5.	Excluded by title
161	Incerti Parenti S, Bortolotti F, Alessandri-Bonetti G. Oral appliances for obstructive sleep apnea. Journal of the World Federation of Orthodontists. 2019;8(1):3-8.	Excluded by title
162	Islam S, Taylor C, Ormiston IW. Effect of preoperative continuous positive airway pressure duration on outcomes after maxillofacial surgery for obstructive sleep apnoea. Br J Oral Maxillofac Surg. 2015;53(2):183-6.	Excluded by title
163	Islam S, Taylor CJ, Ormiston IW. The predictive value of obstructive sleep apnoea severity on clinical outcomes following maxillomandibular advancement surgery. British Journal of Oral and Maxillofacial Surgery. 2015;53(3):263-7.	Excluded by title
164	Itsara M, Sood A, Teuber S. IMMEDIATE HYPERSENSITIVITY TO A DENTAL APPLIANCE CONTAINING NICKEL AND METHYL METHACRYLATE. Annals of Allergy, Asthma and Immunology. 2018;121(5):S73.	Excluded by title
165	Jeong WS, Kim YC, Chung YS, Lee CY, Choi JW. Change in Posterior Pharyngeal Space After Counterclockwise Rotational Orthognathic Surgery for Class II Dentofacial Deformity Diagnosed With Obstructive Sleep Apnea Based on Cephalometric Analysis. J Craniofac Surg. 2017;28(5):e488-e91.	Excluded by title
166	Jia P, Fu M, Zeng X. [Changes of upper airway morphology induced by mandibular advancement in patients with obstructive sleep apnea syndrome]. Beijing Da Xue Xue Bao Yi Xue Ban. 2003;35(6):663-7.	Excluded by title
167	Jiang C, Yi Y, Jiang C, Fang S, Wang J. Pharyngeal Airway Space and Hyoid Bone Positioning After Different Orthognathic Surgeries in Skeletal Class II Patients. J Oral Maxillofac Surg. 2017;75(7):1482-90.	Excluded by title
168	Johal A, Battagel JM. An investigation into the changes in airway dimension and the efficacy of mandibular advancement appliances in subjects with obstructive sleep apnoea. Br J Orthod. 1999;26(3):205-10.	Excluded by title
169	Kandil A, Hammad R, Shafei M, Kabarity R, Ozairy H. Preemptive use of epidural magnesium sulphate to reduce narcotic requirements in orthopedic surgery. Egyptian journal of anaesthesia [Internet]. 2012; 28(1):[17-22 pp.]. Available from: https://www.cochranelibrary.com/central/doi/10.1002/central/CN-00897739/full.	Excluded by title
170	Karacay S, Akin E, Ortakoglu K, Bengi AO. Dynamic MRI evaluation of tongue posture and deglutitive movements in a surgically corrected open bite. Angle Orthod. 2006;76(6):1057-65.	Excluded by title
171	Kastoer C, Op de Beeck S, Dom M, Neirinckx T, Verbraecken J, Braem MJ, et al. Drug-Induced Sleep Endoscopy Upper Airway Collapse Patterns and Maxillomandibular Advancement. Laryngoscope. 2020;130(4):E268-e74.	Excluded by title
172	Keropian B, Murphy NC, Yong M. Treating obstructive sleep apnea. A manifest destiny for 21st century dentists. Dent Today. 2014;33(9):138, 40-2.	Excluded by title
173	Kessler P. Role of neuraxial analgesia/anesthesia in spine surgery. Regional anesthesia and pain medicine. 2016;41(5):e4-e6.	Excluded by title
174	Khandwalla RM, Birkeland KT, Heywood JT, Owens RL, Steinhubl SR, Grant D, et al. AWAKE-HF: rationale and design of a study using a wearable biosensor to objectively evaluate the effect of sacubitril/valsartan initiation on measures of physical activity, symptoms, and sleep, as health-related quality of life functions in subjects with heart failure. Journal of cardiac failure. 2017;23(8):S69-.	Excluded by title
175	Khonsari RH, Way B, Nysjo J, Odri GA, Olszewski R, Evans RD, et al. Fronto-facial advancement and bipartition in Crouzon-Pfeiffer and Apert syndromes: Impact of fronto-facial surgery upon orbital and airway parameters in FGFR2 syndromes. J Craniomaxillofac Surg. 2016;44(10):1567-75.	Excluded by title
176	Kilinc AS, Arslan SG, Kama JD, Ozer T, Dari O. Effects on the sagittal pharyngeal dimensions of protraction and rapid palatal expansion in Class III malocclusion subjects. Eur J Orthod. 2008;30(1):61-6.	Excluded by title
177	Kim KB, McShane PG, McQuilling M, Oliver DR, Schauseil M. Computational airflow analysis before and after maxillomandibular advancement surgery. Journal of the World Federation of Orthodontists. 2016;5(1):2-8.	Excluded by title
178	Kinirons B, Borovickova L. Refresher course: regional anesthesia in the acute trauma setting: defining it's role. Regional anesthesia and pain medicine. 2015;40(5):e51-e4.	Excluded by title
179	Klaassen CB. [Introduction to the treatment of class II malocclusions with Frankel's functional appliance]. Ned Tijdschr Tandheelkd. 1977;84(4):116-22 concl.	Excluded by title
180	Kocabas U, Altay H, Yildirimturk O, Ozkalayci F, Saritas B, Pehlivanoglu S. Successful thrombolysis of a subacute prosthetic valve thrombosis with modified ultra-slow thrombolytic therapy. European Heart Journal Cardiovascular Imaging. 2019;20:i68.	Excluded by title
181	Kocuta M. Apnea en niños: diagnóstico y tratamiento. Rev Ateneo Argent Odontol. 2017;57(2):19-25.	Excluded by title
182	Kohno A, Kitamura Y, Kato S, Imai H, Masuda Y, Sato Y, et al. Displacement of the hyoid bone by muscle paralysis and lung volume increase: the effects of obesity and obstructive sleep apnea. Sleep. 2019;42(1).	Excluded by title
183	Kornegay EC, Brame JL. Obstructive Sleep Apnea and the Role of Dental Hygienists. J Dent Hyg. 2015;89(5):286-92.	Excluded by title
184	Kronbichler A, Shin JI, Lee KH, Nakagomi D, Quintana LF, Busch M, et al. Clinical associations of renal involvement in ANCA-associated vasculitis. Autoimmun Rev. 2020;19(4):102495.	Excluded by title
185	Kyung SH, Park YC, Pae EK. Obstructive sleep apnea patients with the oral appliance experience pharyngeal size and shape changes in three dimensions. Angle Orthodontist. 2005;75(1):15-22.	Excluded by title
186	Laganà F, Rossi D, Romano M, Giannì A. An orthodontic-orthognathic conceptual approach to OSAS surgery: Our experience and results. Sleep Medicine. 2013;14:e246.	Excluded by title
187	Lewkowicz B. Vínculo entre salud oral y envejecimiento saludable. Aporte de la ortodoncia-ortopedia a la salud bucal del adulto mayor: parte 2. Rev Ateneo Argent Odontol. 2019;61(2):41-50.	Excluded by title
188	Leyder P, Altounian G, Quilichini J. Adjustable selective maxillary expansion combined with one-stage maxillomandibular surgery: A prospective study of osseous widening in fifty-five consecutive patients. Journal of Cranio-Maxillofacial Surgery. 2018;46(9):1408-20.	Excluded by title
189	Lim SW, Choi JY, Baek SH. Longitudinal management of recurrent temporomandibular joint ankylosis from infancy to adulthood in perspective of surgical and orthodontic treatment. Korean Journal of Orthodontics. 2019;49(6):413-26.	Excluded by title
190	Lin S-J, Albert C, Drummond-Sibley K, Holmgren M, Ababneh A. Effects of physical activity promotion and inspiratory muscle training in people with chronic heart failure-a preliminary report. Physiotherapy (united kingdom) [Internet]. 2015; 101:[eS884 p.]. Available from: https://www.cochranelibrary.com/central/doi/10.1002/central/CN-01198391/full.	Excluded by title
191	Lin Y, Shen J, Chen L, Yuan W, Cong H, Luo J, et al. Cardiopulmonary Function in Patients with Congenital Scoliosis: An Observational Study. J Bone Joint Surg Am. 2019;101(12):1109-18.	Excluded by title
192	Lin Y, Tan H, Rong T, Chen C, Shen J, Liu S, et al. Impact of Thoracic Cage Dimension and Geometry on Cardiopulmonary Function in Patients With Congenital Scoliosis: A Prospective Study. Spine (Phila Pa 1976). 2019;44(20):1441-8.	Excluded by title
193	Liu Y, Zeng X, Fu M, Huang X, Lowe AA. Effects of a mandibular repositioner on obstructive sleep apnea. Am J Orthod Dentofacial Orthop. 2000;118(3):248-56.	Excluded by title
194	Lo Presti D, Carnevale A, D'Abbraccio J, Massari L, Massaroni C, Sabbadini R, et al. A Multi-Parametric Wearable System to Monitor Neck Movements and Respiratory Frequency of Computer Workers. Sensors (Basel). 2020;20(2).	Excluded by title
195	Long L, Mordi I, Bridges C, Sagar V, Davies E, Coats A, et al. Exercise-based cardiac rehabilitation for adults with heart failure. Cochrane Database of Systematic Reviews [Internet]. 2019; (1). Available from: http://dx.doi.org/10.1002/14651858.CD003331.pub5.	Excluded by title
196	Lorenz GI, Ceccarelli AA, Rivas NH. Las mesiorrelaciones subclínicas: su diagnóstico. Rev Asoc Argent Ortop Funcional Maxilares. 2002;33(1):39-43.	Excluded by title
197	Luu TH, Roberts MM, Gadulov Y, Fields KG, Kahn RL, Gulotta LV, et al. Recovery profile among orthopedic patients receiving peripheral nerve blocks. A pilot study. Regional anesthesia and pain medicine. 2017;42(6).	Excluded by title
198	Lye KW. Effect of orthognathic surgery on the posterior airway space (PAS). Ann Acad Med Singapore. 2008;37(8):677-82.	Excluded by title
199	Maia S, Raveli DB, dos Santos-Pinto A, Raveli TB, Gomez SP. Computed Tomographic evaluation of a young adult treated with the Herbst appliance. Dental Press Journal of Orthodontics. 2010;15(5):130-6.	Excluded by title
200	Mann V, Limberg F, Mann STW, Little S, Muller M, Sander M, et al. [Skill retention using extraglottic airways in out-of-hospital emergencies: efficacy and long-term results of simulator-based medical education : A prospective follow-up study]. Med Klin Intensivmed Notfmed. 2018.	Excluded by title

201	Mann V, Limberg F, Mann STW, Little S, Müller M, Sander M, et al. [Skill retention using extraglottic airways in out-of-hospital emergencies: efficacy and long-term results of simulator-based medical education : A prospective follow-up study]. Med Klin Intensivmed Notfmed. 2019;114(6):541-51.	Excluded by title
202	Marcu I, Patru S, Matei D, Bighea A. Role of physical exercise in patients with knee arthroplasty for osteoarthritis. Osteoporosis international [Internet]. 2017; 28:[S330- pp.]. Available from: https://www.cochranelibrary.com/central/doi/10.1002/central/CN-01409681/full.	Excluded by title
203	Martín Romero M, Reina Marfil N, Ortega Sáenz De Tejada E, Hidalgo Sánchez R, Amecrane N, Hidalgo Sanjuán M. Oral appliances in obstructive sleep apnea syndrome: Our experience. Sleep Medicine. 2013;14:e247-e8.	Excluded by title
204	Martinez-Anton A, Eberlein M, Logun C, Kern S, Danner R, Shelhamer JH. Effect of interferon-gamma on gene expression in air-liquid interface differentiated Normal Human Bronchial Epithelial Cells (NHBECS). American Journal of Respiratory and Critical Care Medicine. 2011;183(1).	Excluded by title
205	Matsumoto H, Kasai T, Suda S, Yatsu S, Shitara J, Murata A, et al. Randomized controlled trial of an oral appliance (SomnoDent) for sleep-disordered breathing and cardiac function in patients with heart failure. Clinical cardiology [Internet]. 2018; (no pagination). Available from: https://www.cochranelibrary.com/central/doi/10.1002/central/CN-01644663/full.	Excluded by title
206	Mattos CT, Vilani GN, Sant'Anna EF, Ruellas AC, Maia LC. Effects of orthognathic surgery on oropharyngeal airway: a meta-analysis. Int J Oral Maxillofac Surg. 2011;40(12):1347-56.	Excluded by title
207	Matuska P, Kara T, Homolka P, Belehrad M. Advances in the management of sleep-disordered breathing in heart failure. Cor ET vasa. 2013;55(5):E411-E8.	Excluded by title
208	Melnik AK. Orthodontic movement of a supplemental maxillary incisor through the midpalatal suture area. Am J Orthod Dentofacial Orthop. 1993;104(1):85-90.	Excluded by title
209	Meslemani D, Jones LR. Skeletal surgery in sleep apnea. Curr Opin Otolaryngol Head Neck Surg. 2011;19(4):307-11.	Excluded by title
210	Metz JE, Attarian HP, Harrison MC, Blank JE, Takacs CM, Smith DL, et al. High-resolution pulse oximetry and titration of a mandibular advancement device for obstructive sleep apnea. Frontiers in Neurology. 2019;10(JUL).	Excluded by title
211	Milano F, Billi MC, Marra F, Sorrenti G, Gracco A, Bonetti GA. Factors associated with the efficacy of mandibular advancing device treatment in adult OSA patients. Int Orthod. 2013;11(3):278-89.	Excluded by title
212	Miller D, Lewis S, Pritchard M, Schofield-Robinson O, Shelton C, Alderson P, et al. Intravenous versus inhalational maintenance of anaesthesia for postoperative cognitive outcomes in elderly people undergoing non-cardiac surgery. Cochrane Database of Systematic Reviews [Internet]. 2018; (8). Available from: http://dx.doi.org/10.1002/14651858.CD012317.pub2.	Excluded by title
213	Mitani Y, Banabilh SM, Singh GD. Craniofacial changes in patients with Class III malocclusion treated with the RAMPA system. Int J Orthod Milwaukee. 2010;21(2):19-25.	Excluded by title
214	Momomura S-I, null, Momomura S, Wada H, Sugawara Y, Ikeda N, et al. Adaptive servo-ventilation therapy for patients with chronic heart failure in a confirmatory, multicenter, randomized, controlled study. Circulation journal [Internet]. 2015; 79(5):[982-90 pp.]. Available from: https://www.cochranelibrary.com/central/doi/10.1002/central/CN-01070100/full.	Excluded by title
215	Mucedero M, Baccetti T, Franchi L, Cozza P. Effects of maxillary protraction with or without expansion on the sagittal pharyngeal dimensions in Class III subjects. Am J Orthod Dentofacial Orthop. 2009;135(6):777-81.	Excluded by title
216	Mutoh M, Kimura S, Takahashi-Iwanaga H, Hisamoto M, Iwanaga T, Iida J. RANKL regulates differentiation of microfold cells in mouse nasopharynx-associated lymphoid tissue (NALT). Cell Tissue Res. 2016;364(1):175-84.	Excluded by title
217	Nakamura M, Yanagita T, Matsumura T, Yamashiro T, Iida S, Kamioka H. A case of severe mandibular retrognathism with bilateral condylar deformities treated with Le Fort I osteotomy and two advancement genioplasty procedures. Korean J Orthod. 2016;46(6):395-408.	Excluded by title
218	Nisser J, Smolenski U, Sliwinski GE, Schumann P, Heinke A, Malberg H, et al. The FED-Method (Fixation, Elongation, Derotation) - a Machine-supported Treatment Approach to Patients with Idiopathic Scoliosis - Systematic Review. Z Orthop Unfall. 2020;158(3):318-32.	Excluded by title
219	Nunes WR, Francesco-Mion RCD. Early treatment and preventive strategies for obstructive sleep apnea and hypopnea with the bioajusta x orthodontic- orthopedic treatment. Sleep Medicine. 2009;10:S41-S2.	Excluded by title
220	Okushi T, Sano C, Arisaka T, Kobayashi S, Sato K, Tonoki M, et al. Effect of maxillofacial surgery on the morphology of nasopharyngeal space in Japanese patient. Sleep. 2009;32:A201.	Excluded by title
221	Oldroyd K, Gray C, Carter R, Harvey K, Borland W, Beastall G, et al. Activation and inhibition of the endogenous opioid system in human heart failure. British heart journal [Internet]. 1995; 73(1):[41-8 pp.]. Available from: https://www.cochranelibrary.com/central/doi/10.1002/central/CN-00111849/full.	Excluded by title
222	Orrell R, Copeland S, Rose M. Scapular fixation in muscular dystrophy. Cochrane Database of Systematic Reviews [Internet]. 2010; (1). Available from: http://dx.doi.org/10.1002/14651858.CD003278.pub2.	Excluded by title
223	Oswal P, Nagarathna R, Ebnezar J, Nagendra H. The effect of add-on yogic prana energization technique (YPET) on healing of fresh fractures: a randomized control study. Journal of alternative and complementary medicine (new york, NY) [Internet]. 2011; 17(3):[253-8 pp.]. Available from: https://www.cochranelibrary.com/central/doi/10.1002/central/CN-00801189/full.	Excluded by title
224	Otsuka R, Almeida FR, Lowe AA, Ryan F. A comparison of responders and nonresponders to oral appliance therapy for the treatment of obstructive sleep apnea. Am J Orthod Dentofacial Orthop. 2006;129(2):222-9.	Excluded by title
225	Owen AH, 3rd. Morphologic changes in the transverse dimension using the Frankel appliance. Am J Orthod. 1983;83(3):200-17.	Excluded by title
226	Owens RL, Birkeland K, Heywood JT, Steinhubl S, Dorn J, Grant D, et al. SLEEP OUTCOMES FROM AWAKE-HF, A RANDOMIZED CLINICAL TRIAL WITH OPEN-LABEL EXTENSION OF SACUBITRIL/VALSARTAN VERSUS ENALAPRIL IN PATIENTS WITH HEART FAILURE WITH REDUCED EJECTION FRACTION. Journal of the american college of cardiology. 2019;73(9):849-.	Excluded by title
227	Park B, Chung MY. Effect of nasal high flow system on oxygenation during sedation under spinal anesthesia in elderly patients. Respirology (carlton, vic). 2017;22:228-.	Excluded by title
228	Paz-Alegría MC, Gómez-Forero D, Osorio-Patiño J, Jaramillo-Echeverry A. Behavioral and dental management of a patient with Tatton-Brown-Rahman syndrome: Case report. Special Care in Dentistry. 2020.	Excluded by title
229	Pennington L, Parker N, Kelly H, Miller N. Speech therapy for children with dysarthria acquired before three years of age. Cochrane Database of Systematic Reviews [Internet]. 2016; (7). Available from: http://dx.doi.org/10.1002/14651858.CD006937.pub3.	Excluded by title
230	Pepperell J, Maskell N, Jones D, Langford-Wiley B, Crosthwaite N, Stradling J, et al. A randomized controlled trial of adaptive ventilation for Cheyne-Stokes breathing in heart failure. American journal of respiratory and critical care medicine [Internet]. 2003; 168(9):[1109-14 pp.]. Available from: https://www.cochranelibrary.com/central/doi/10.1002/central/CN-00457457/full.	Excluded by title
231	Pettit NJ, Auvenshine RC. Change of hyoid bone position in patients treated for and resolved of myofascial pain. Cranio-the Journal of Craniomandibular & Sleep Practice. 2020;38(2):74-90.	Excluded by title
232	Pinto ML, Felix MMR, Bordalo C, Silva SH, Do Carmo Ferrão K, De Britto Pereira Bandeira De Mello M, et al. A survey study of medical knowledge about anaphylaxis. World Allergy Organization Journal. 2015;8.	Excluded by title
233	Polverino F, Lu B, Quintero JR, Vargas SO, Patel AS, Owen CA, et al. CFTR regulates B cell activation and lymphoid follicle development. Respir Res. 2019;20(1):133.	Excluded by title
234	Ponikowski P, Oldenburg O, Javaheri S, Goldberg L, Stellbrink C, Kao AC, et al. Treatment of central sleep apnoea with phrenic nerve stimulation is associated with reduction in heart failure-related hospitalizations. European journal of heart failure. 2019;21:346-.	Excluded by title
235	Posnick JC, Adachie A, Singh N, Choi E. "Silent" Sleep Apnea in Dentofacial Deformities and Prevalence of Daytime Sleepiness After Orthognathic and Intranasal Surgery. Journal of Oral and Maxillofacial Surgery. 2018;76(4):833-43.	Excluded by title
236	Posnick JC, Egolum N, Tremont TJ. Primary Mandibular Deficiency Dentofacial Deformities: Occlusion and Facial Aesthetic Surgical Outcomes. Journal of Oral and Maxillofacial Surgery. 2018;76(10):2209.e1-.e15.	Excluded by title
237	Posnick JC, Liu S, Tremont TJ. Long-Face Dentofacial Deformities: Occlusion and Facial Esthetic Surgical Outcomes. Journal of Oral and Maxillofacial Surgery. 2018;76(6):1291-308.	Excluded by title
238	Posnick JC, Nakano P, Taylor M. A modified occlusal splint to avoid tracheotomy for total midface osteotomies. Ann Plast Surg. 1992;29(3):223-30.	Excluded by title

239	Posnick JC. Craniofacial dysostosis. Staging of reconstruction and management of the midface deformity. Neurosurg Clin N Am. 1991;2(3):683-702.	Excluded by title
240	Qu SY, Ti XY, Zhang J, Wu CG. Disruption of the Notch pathway aggravates airway inflammation by inhibiting regulatory T cell differentiation via regulation of plasmacytoid dendritic cells. Scand J Immunol. 2020;91(5):e12865.	Excluded by title
241	Quesada MB, Ferreyra JD, González MA. ¿Qué hay detrás de la boca? Rev Asoc Argent Ortop Funcional Maxilares. 2016;41(2):9-13.	Excluded by title
242	Quintela MdM, Lima Júnior A, Sallum RA, Pacheco Filho F, Flório FM, Motta RHL. Avaliação da eficácia de um aparelho de avanço mandibular semiflexível na apneia obstrutiva do sono: estudo clínico e polissonográfico. Ortodontia. 2013;46(4):358-69.	Excluded by title
243	Quintela MdM, Pacheco Filho F, Uechi CH. O exercício da medicina do sono na clínica ortodôntica: pesquisas, protocolos e perspectivas. Ortho Sci, Orthod sci pract. 2010;3(9):43-50.	Excluded by title
244	Quintela MdM. Descrição de um aparelho intra-oral para o tratamento dos distúrbios respiratórios do sono. 2009:68-.	Excluded by title
245	rachana mandru MBBS, Grewal H, Gada K, Paul M. RIGHT VENTRICULAR THROMBUS (RVT) AIDING IN DIAGNOSIS OF ADENOCARCINOMA. Chest. 2019;156(4):A607.	Excluded by title
246	Rajagopalan G, Iijima K, Singh M, Kita H, Patel R, David CS. Intranasal exposure to bacterial superantigens induces airway inflammation in HLA class II transgenic mice. Infect Immun. 2006;74(2):1284-96.	Excluded by title
247	Rbr zf. Comparison of training with video games and conventional physiotherapy on balance, gait, breathing, memory and quality of life of the elderly: a randomized clinical trial. Http://wwwensaiosclinicosgovbr/rg/rbr-4z4f48/ [Internet]. 2016. Available from: https://www.cochranelibrary.com/central/doi/10.1002/central/CN-01308231/full.	Excluded by title
248	Rechnitzer PA, Cunningham DA, Jones N. A controlled prospective study of the effect of endurance training on the recurrence rate of myocardial infarction. The Ontario exercise heart collaborative trial. An interim report. Cardiology. 1977;62(2):no. 53.	Excluded by title
249	Resnick CM, Frid P, Norholt SE, Stoustrup P, Peacock ZS, Kaban LB, et al. An Algorithm for Management of Dentofacial Deformity Resulting From Juvenile Idiopathic Arthritis: Results of a Multinational Consensus Conference. Journal of Oral and Maxillofacial Surgery. 2019;77(6):1152.e1-.e33.	Excluded by title
250	Rief M, Zoidl P, Zajic P, Heschl S, Orlob S, Silbernagel G, et al. Atlanto-occipital dislocation in a patient presenting with out-of-hospital cardiac arrest: a case report and literature review. J Med Case Rep. 2019;13(1):44.	Excluded by title
251	Riepponen A, Myllykangas R, Savolainen J, Kilpelainen P, Kellokoski J, Pahkala R. Changes in posterior airway space and hyoid bone position after surgical mandibular advancement. Acta Odontol Scand. 2017;75(1):73-8.	Excluded by title
252	Riguey Mira L N. Odontología y trastornos respiratorios: apena del sueño. Rev Fac Odontol Univ Antioq. 2004;15(1):40-52.	Excluded by title
253	Ristow O, Ruckschloss T, Berger M, Grotz T, Kargus S, Krisam J, et al. Short- and long-term changes of the pharyngeal airway after surgical mandibular advancement in Class II patients-a three-dimensional retrospective study. J Craniomaxillofac Surg. 2018;46(1):56-62.	Excluded by title
254	Robertson CJ. The effect of long-term mandibular advancement on the hyoid bone and pharynx as it relates to the treatment of obstructive sleep apnoea. Aust Orthod J. 2000;16(3):157-66.	Excluded by title
255	Rodriguez RF, Townsend NE, Aughey RJ, Billaut F. Muscle oxygenation maintained during repeated-sprints despite inspiratory muscle loading. PLoS One. 2019;14(9):e0222487.	Excluded by title
256	Ronconi TC. Recursos terapéuticos. Presentación de un caso. Rev Asoc Argent Ortop Funcional Maxilares. 1994;27(73/74):45-53.	Excluded by title
257	Saletu A, Parapatics S, Saletu B, Anderer P, Prause W, Putz H, et al. On the pharmacotherapy of sleep bruxism: placebo-controlled polysomnographic and psychometric studies with clonazepam. Neuropsychobiology [Internet]. 2005; 51(4):[214-25 pp.]. Available from: https://www.cochranelibrary.com/central/doi/10.1002/central/CN-00511749/full.	Excluded by title
258	Sam K, Lam B, Ooi CG, Cooke M, Ip MS. Effect of a non-adjustable oral appliance on upper airway morphology in obstructive sleep apnoea. Respir Med. 2006;100(5):897-902.	Excluded by title
259	Santos Junior JF, Abrahao M, Gregorio LC, Zonato AI, Gumieiro EH. Genioplasty for genioglossus muscle advancement in patients with obstructive sleep apnea-hypopnea syndrome and mandibular retrognathia. Braz J Otorhinolaryngol. 2007;73(4):480-6.	Excluded by title
260	Sathiya K, Kumar M. Effectiveness of myotherapy on pain among patients subjected to major orthopaedic surgery at a tertiary care hospital. Research journal of pharmacy and technology. 2019;12(11):5399-406.	Excluded by title
261	Schneider S, Witt E. [The functional findings before and after a combined orthodontic and oral surgical treatment of Angle class-III patients]. Fortschr Kieferorthop. 1991;52(1):51-9.	Excluded by title
262	Sears CR, Miller AJ, Chang MK, Huang JC, Lee JS. Comparison of pharyngeal airway changes on plain radiography and cone-beam computed tomography after orthognathic surgery. J Oral Maxillofac Surg. 2011;69(11):e385-94.	Excluded by title
263	Shaeran TAT, Samsudin AR. Temporomandibular Joint Ankylosis Leading to Obstructive Sleep Apnea. J Craniofac Surg. 2019;30(8):e714-e7.	Excluded by title
264	Shaikh S, Turk A, Grewal V, Slawsky M, Hernandez-Montfort J. Right ventricular stress relief by intra-pulmonary shunting in portopulmonary hypertension. Journal of Cardiac Failure. 2016;22:S131-S2.	Excluded by title
265	Sharma I, Rana S, Choudhary B, Dhiman T, Sharma S, Kumar M. Comparative analgesic efficacy of intravenous vs intrathecal dexmedetomidine as an adjuvant to hyperbaric bupivacaine in subarachnoid block for below knee orthopaedic surgery. Indian journal of anaesthesia. 2020;64(6):463-9.	Excluded by title
266	Shete CS, Bhad WA. Three-dimensional upper airway changes with mandibular advancement device in patients with obstructive sleep apnea. Am J Orthod Dentofacial Orthop. 2017;151(5):941-8.	Excluded by title
267	Shoemaker MJ, Dias KJ, Lefebvre KM, Heick JD, Collins SM. Physical Therapist Clinical Practice Guideline for the Management of Individuals With Heart Failure. Phys Ther. 2020;100(1):14-43.	Excluded by title
268	Shokri A, Mollabashi V, Zahedi F, Tapak L. Position of the hyoid bone and its correlation with airway dimensions in different classes of skeletal malocclusion using cone-beam computed tomography. Imaging Science in Dentistry. 2020;50(2):105-15.	Excluded by title
269	Silva PRDd, Silva PPDd, Dias LPT, Domaneschi C, Uvo SAB. Tratamento cirúrgico para apnéia obstrutiva do sono. Ortodontia. 2010;43(1):63-8.	Excluded by title
270	Simmonds JC, Hollis RJ, Tamberino RK, Vecchiotti MA, Scott AR. Comparing the real and perceived cost of adenotonsillectomy using time-driven activity-based costing. Laryngoscope. 2019;129(6):1347-53.	Excluded by title
271	Skilton M, Krishan A, Patel S, Sinha I, Southern K. Potentiators (specific therapies for class III and IV mutations) for cystic fibrosis. Cochrane Database of Systematic Reviews [Internet]. 2019; (1). Available from: http://dx.doi.org/10.1002/14651858.CD009841.pub3.	Excluded by title
272	Solem RC, Martz M, Weiss E, Reese P, Kawamoto H, Lee JC. Multidisciplinary Treatment of Antley-Bixler Syndrome. Cleft Palate Craniofac J. 2017;54(1):100-8.	Excluded by title
273	Sônia Rodrigues Álvares R, Beleigoli AM, De Moura Nigri Hilário Ferreira M, Guilherme De Oliveira Freitas L, Gama Van Eijk V, De Araújo Silva L, et al. Resilience and social support are determinants of quality of life after bariatric surgery: Results of the 9-year follow-up of a Brazilian cohort. Obesity Facts. 2018;11:238.	Excluded by title
274	Southern K, Patel S, Sinha I, Nevitt S. Correctors (specific therapies for class II CFTR mutations) for cystic fibrosis. Cochrane Database of Systematic Reviews [Internet]. 2018; (8). Available from: http://dx.doi.org/10.1002/14651858.CD010966.pub2.	Excluded by title
275	Souza EdCVd. Abordagem odontológica dos dirtúrbios do sono. 2012:23-.	Excluded by title
276	Spiegel R, Haefelfinger R, Sutter R, Bingisser R. An unexpected cause of shock in a patient with tachycardiomyopathy. Critical Care Medicine. 2018;46:143.	Excluded by title
277	Starr BW, Morrison JM, Billmire DA. Orthognathic surgery in pediatric burn patients. Journal of Burn Care and Research. 2019;40:S215.	Excluded by title
278	Steffanina A, Proietti L, Antonaglia C, Palange P, Angelici E, Canipari R. The Plasminogen System and Transforming Growth Factor-beta in Subjects With Obstructive Sleep Apnea Syndrome: Effects of CPAP Treatment. Respir Care. 2015;60(11):1643-51.	Excluded by title
279	Sujuan F, Taimei SHI. Application of breath-guided active flexion and extension of foot and ankle joint in prevention of lower extremity deep venous thrombosis after orthopedic surgery. Chinese nursing research. 2020;34(12):2126-31.	Excluded by title
280	Sun L, Zhao J, Wang H, Pan Y, Wang L, Zhang WB. Mechanical stress promotes matrix synthesis of mandibular condylar cartilage via the RKIP-ERK pathway. J Mol Histol. 2017;48(5-6):437-46.	Excluded by title

281	Sutherland K, Chan ASL, Deane SA, Zeng B, Lee RWW, Darendeliler MA, et al. Comparison of the effects of two oral appliances on upper airway anatomy in obstructive sleep aponea. Sleep and Biological Rhythms. 2009;7:A38.	Excluded by title
282	Tan SK, Tang ATH, Leung WK, Zwahlen RA. Three-dimensional pharyngeal airway changes in dento-skeletal class II patients after two-jaw orthognathic surgery with segmentation - a pilot study. J Stomatol Oral Maxillofac Surg. 2018;119(6):461-8.	Excluded by title
283	Tanaka O, Costa D, Niwa M, Bastos S, Pithon M. Surgery-first approach in a case of mandibular asymmetry. European Journal of General Dentistry. 2020;9(3):181-9.	Excluded by title
284	Tanellari O, Baruti E, Savin C, Costan V, Olteanu D, Feier R, et al. Surgical Treatment of Obstructive Sleep Apnea - Case Report. Romanian Journal of Oral Rehabilitation. 2020;12(1):208-14.	Excluded by title
285	Taylor JA, Bartlett SP. What's New in Syndromic Craniosynostosis Surgery? Plast Reconstr Surg. 2017;140(1):82e-93e.	Excluded by title
286	Taylor S, Frost H, Taylor A, Barker K. Reliability and responsiveness of the shuttle walking test in patients with chronic low back pain. Physiotherapy research international [Internet]. 2001; 6(3):[170-8 pp.]. Available from: https://www.cochranelibrary.com/central/doi/10.1002/central/CN-00472059/full.	Excluded by title
287	Teixeira AOdB. Uso de aparelho de protrusão mandibular como recurso para tratamento da síndrome da apnéia obstrutiva do sono. 2008:119-.	Excluded by title
288	Templier L, Rossi C, Miguez M, Pérez JDLC, Curto A, Albaladejo A, et al. Combined surgical and orthodontic treatments in children with OSA: A systematic review. Journal of Clinical Medicine. 2020;9(8):1-18.	Excluded by title
289	Teschler H, Cowie M, D'Ortho M, Angermann C, Erdmann E, Levy P, et al. Rationale and design of the serve HF study: treatment of sleep-disordered breathing with predominant central sleep apnea by adaptive servo ventilation in patients with heart failure. American journal of respiratory and critical care medicine [Internet]. 2010; 181(1 MeetingAbstracts). Available from: https://www.cochranelibrary.com/central/doi/10.1002/central/CN-01031598/full.	Excluded by title
290	Tihacek-Sojic L, Andjelkovic M, Milic-Lemic A, Milosevic B. The effectiveness of oral appliances in elderly patients with obstructive sleep apnoea treated with lorazepam - a pilot study. Journal of Oral Rehabilitation. 2012;39(10):785-90.	Excluded by title
291	Tomonari H, Takada H, Hamada T, Kwon S, Sugiura T, Miyawaki S. Micrognathia with temporomandibular joint ankylosis and obstructive sleep apnea treated with mandibular distraction osteogenesis using skeletal anchorage: a case report. Head Face Med. 2017;13(1):20.	Excluded by title
292	Tsuiki S, Almeida FR, Lowe AA, Su J, Fleetham JA. The interaction between changes in upright mandibular position and supine airway size in patients with obstructive sleep apnea. Am J Orthod Dentofacial Orthop. 2005;128(4):504-12.	Excluded by title
293	Tsuiki S, Lowe AA, Almeida FR, Fleetham JA. Effects of an anteriorly titrated mandibular position on awake airway and obstructive sleep apnea severity. Am J Orthod Dentofacial Orthop. 2004;125(5):548-55.	Excluded by title
294	Turnbull NR, Battagel JM. The effects of orthognathic surgery on pharyngeal airway dimensions and quality of sleep. J Orthod. 2000;27(3):235-47.	Excluded by title
295	Turvey TA. Skeletal surgery for mandibulofacial dysostosis (Treacher Collins, Nager Syndrome). International Journal of Oral and Maxillofacial Surgery. 2017;46:4.	Excluded by title
296	Vaisberg M, Paixão V, Almeida EB, Santos JMB, Foster R, Rossi M, et al. Daily Intake of Fermented Milk Containing Lactobacillus casei Shirota (Lcs) Modulates Systemic and Upper Airways Immune/Inflammatory Responses in Marathon Runners. Nutrients. 2019;11(7).	Excluded by title
297	Valladares-Neto J, Silva MA, Bumann A, Paiva JB, Rino-Neto J. Effects of mandibular advancement surgery combined with minimal maxillary displacement on the volume and most restricted cross-sectional area of the pharyngeal airway. Int J Oral Maxillofac Surg. 2013;42(11):1437-45.	Excluded by title
298	van Ginkel S, de Haan A, Woerdeman J, Vanhees L, Serné E, de Koning J, et al. Exercise intensity modulates capillary perfusion in correspondence with ACE I/D modulated serum angiotensin II levels. Applied and Translational Genomics. 2015.	Excluded by title
299	van Sickels JE, Wallender A. Closure of anterior open bites with mandibular surgery: Advantages and disadvantages of this approach. Oral and Maxillofacial Surgery. 2012;16(4):361-7.	Excluded by title
300	Vigneron A, Tamisier R, Orset E, Pepin JL, Bettega G. Maxillomandibular advancement for obstructive sleep apnea syndrome treatment: Long-term results. J Craniomaxillofac Surg. 2017;45(2):183-91.	Excluded by title
301	Villalba Rodriguez A, Felzani Moreno R, Vega Lagos O. Changes in the pharyngeal airway space based on cephalometric study after orthognathic surgery. International Journal of Oral and Maxillofacial Surgery. 2011;40(10):1086-7.	Excluded by title
302	von Treuenfels H. Empresa internacional para medicina dental integral. Ortopedia maxilar con focalización integral. Información para odontólogos, médicos, terapeutas y pacientes (trad). Rev Asoc Argent Ortop Funcional Maxilares. 1994;27(73/74):7-13.	Excluded by title
303	Wang M, Wang K, Deng G, Liu X, Wu X, Hu H, et al. Mitochondria-Modulating Porous Se@SiO(2) Nanoparticles Provide Resistance to Oxidative Injury in Airway Epithelial Cells: Implications for Acute Lung Injury. Int J Nanomedicine. 2020;15:2287-302.	Excluded by title
304	Wang X, Gong X, Yu Z, Gao X, Zhao Y. Follow-up study of dental and skeletal changes in patients with obstructive sleep apnea and hypopnea syndrome with long-term treatment with the Silensor appliance. Am J Orthod Dentofacial Orthop. 2015;147(5):559-65.	Excluded by title
305	Ward P, Kotecha A, Lee C, Collier J, Jin Y, Chae W. Orthognathic surgery: Development of a multidisciplinary patient pathway. Trends in Anaesthesia and Critical Care. 2020;30:e134.	Excluded by title
306	Watakabe K, Yonemitsu I, Ikeda Y, Huan T, Ono T. Unilateral nasal obstruction induces morphological changes of the mandibular condyle in growing rats. Orthodontic Waves. 2018;77(3):157-68.	Excluded by title
307	Wiley D, Yepes JF, Sanders BJ, Jones JE, Johnson KB, Tang Q. Pediatric Phantom Dosimetry Evaluation of the Extraoral Bitewing. Pediatr Dent. 2020;42(1):41-6.	Excluded by title
308	Wittmer V, Simoes G, Sogame L, Vasquez E. Effects of continuous positive airway pressure on pulmonary function and exercise tolerance in patients with congestive heart failure. Chest [Internet]. 2006; 130(1):[157-63 pp.]. Available from: https://www.cochranelibrary.com/central/doi/10.1002/central/CN-00566526/full.	Excluded by title
309	Wolford LM, Goncalves JR. Condylar Resorption of the Temporomandibular Joint: How Do We Treat It? Oral and Maxillofacial Surgery Clinics of North America. 2015;27(1):47-+.	Excluded by title
310	Xu C, Wu X, Lu M, Tang L, Yao H, Wang J, et al. Protein tyrosine phosphatase 11 acts through RhoA/ROCK to regulate eosinophil accumulation in the allergic airway. Faseb j. 2019;33(11):11706-20.	Excluded by title
311	Yamada K, Abe Y, Satoh S, Yanagibashi Y, Hyakumachi T, Masuda T. Large Increase in Blood Pressure After Extubation and High Body Mass Index Elevate the Risk of Spinal Epidural Hematoma After Spinal Surgery. Spine (Phila Pa 1976). 2015;40(13):1046-52.	Excluded by title
312	Yamashita AL, Filho LI, Da Silva MC, Takeshita WM, Iwaki LCV. Effect of orthognathic surgery on pharyngeal airway space: a cephalometric evaluation using dolphin imaging software. Acta Scientiarum - Health Sciences. 2015;37(2):205-9.	Excluded by title
313	Yisheng W, Fuying Z, Limin W, Junwei L, Guofu P, Weidong W. First aid and treatment for cervical spinal cord injury with fracture and dislocation. Indian J Orthop. 2007;41(4):300-4.	Excluded by title
314	Yu LM, Zhang WH, Han XX, Li YY, Lu Y, Pan J, et al. Hypoxia-Induced ROS Contribute to Myoblast Pyroptosis during Obstructive Sleep Apnea via the NF-κB/HIF-1α Signaling Pathway. Oxid Med Cell Longev. 2019;2019:4596368.	Excluded by title
315	Yue S, Wang Z, Wang L, Peng Q, Xue B. Transcriptome functional analysis of mammary gland of cows in heat stress and thermoneutral condition. Animals. 2020;10(6):1-18.	Excluded by title
316	zccgr RBR. Effects of complementary therapies associated with Cardiac Rehabilitation in patients with Chronic Heart Failure. http://wwwhoint/trialsearch/Trial2aspx?TrialID=RBR-7zccgr. 2018.	Excluded by title
317	Zhao X, Liu Y, Gao Y. Three-dimensional upper-airway changes associated with various amounts of mandibular advancement in awake apnea patients. Am J Orthod Dentofacial Orthop. 2008;133(5):661-8.	Excluded by title
318	Zheng S, Xu W, Bose S, Banerjee AK, Haque SJ, Erzurum SC. Impaired nitric oxide synthase-2 signaling pathway in cystic fibrosis airway epithelium. Am J Physiol Lung Cell Mol Physiol. 2004;287(2):L374-81.	Excluded by title
319	Zhu T, Chen Z, Chen G, Wang D, Tang S, Deng H, et al. Curcumin Attenuates Asthmatic Airway Inflammation and Mucus Hypersecretion Involving a PPARγ-Dependent NF-κB Signaling Pathway In Vivo and In Vitro. Mediators Inflamm. 2019;2019:4927430.	Excluded by title

320	Bakradze A, Vadachkoria Z, Kvachadze I. ELECTROPHYSIOLOGICAL CORRELATES OF MASTICATORY MUSCLES IN NASAL AND ORAL BREATHING MODES. Georgian Med News. 2020(303):55-8.	Excluded by abstract
321	[No authors] 20th Anniversary Meeting of the American Academy of Dental Sleep Medicine. Sleep and Breathing. 2011;15(2).	Excluded by abstract
322	{NCT} Effects of Inspiratory Muscle Training on Dyspnea Perception During Exercise in Patients With COPD. Https://clinicaltrialsgov/show/nct01900873 [Internet]. 2013. Available from: https://www.cochranelibrary.com/central/doi/10.1002/central/CN-01488949/full.	Excluded by abstract
323	{NCT} Feasibility & Effect of a Tele-rehabilitation Program for Chronic Obstructive Pulmonary Disease vs. Standard Rehabilitation. Https://clinicaltrialsgov/show/nct03569384 [Internet]. 2018. Available from: https://www.cochranelibrary.com/central/doi/10.1002/central/CN-01660652/full.	Excluded by abstract
324	Aarab G, Lobbezoo F, Hamburger HL, Naeije M. Effects of an oral appliance with different mandibular protrusion positions at a constant vertical dimension on obstructive sleep apnea. Clin Oral Investig. 2010;14(3):339-45.	Excluded by abstract
325	Abramson Z, Susarla SM, Lawler M, Bouchard C, Troulis M, Kaban LB. Three-dimensional computed tomographic airway analysis of patients with obstructive sleep apnea treated by maxillomandibular advancement. J Oral Maxillofac Surg. 2011;69(3):677-86.	Excluded by abstract
326	Achmad H, Tahir H, Adam M, Ramadhany YF. Increased overjet in growing child, problem solving in Pediatric Dentistry. Journal of International Dental and Medical Research. 2017;10(2):374-9.	Excluded by abstract
327	Agbaje JO, Salem AS, Lambrichts I, Braem M, Politis C. Possible association between mandibular repositioning device for sleep apnea and osseous lytic lesion with fracture of the coronoid process of the mandible: A case report. Quintessence Int. 2016;47(2):141-5.	Excluded by abstract
328	Agostino P, Ugolini A, Signori A, Silvestrini-Biavati A, Harrison J, Riley P. Orthodontic treatment for posterior crossbites. Cochrane Database of Systematic Reviews [Internet]. 2014; (8). Available from: http://dx.doi.org/10.1002/14651858.CD000979.pub2.	Excluded by abstract
329	Alessandri-Bonetti G, Bortolotti F, Bartolucci ML, Marini I, D'Anto V, Michelotti A. The Effects of Mandibular Advancement Device on Pressure Pain Threshold of Masticatory Muscles: A Prospective Controlled Cohort Study. J Oral Facial Pain Headache.30(3):234-40.	Excluded by abstract
330	Alessandri-Bonetti G, D'Anto V, Stipa C, Rongo R, Incerti-Parenti S, Michelotti A. Dentoskeletal effects of oral appliance wear in obstructive sleep apnoea and snoring patients. Eur J Orthod. 2017;39(5):482-8.	Excluded by abstract
331	Alía García E, Martinez-Gónzalez A, Cruz Pérez Jdl. Eficacia y efectos adversos de los aparatos intraorales en el tratamiento de la apnea obstructiva del sueño. Cient dent (Ed impr). 2010;7(2):99-106.	Excluded by abstract
332	Al-Jewair TS, Gaffar BO, Flores-Mir C. Quality Assessment of Systematic Reviews on the Efficacy of Oral Appliance Therapy for Adult and Pediatric Sleep-Disordered Breathing. J Clin Sleep Med. 2016;12(8):1175-83.	Excluded by abstract
333	Almeida FR, Lowe AA. Principles of oral appliance therapy for the management of snoring and sleep disordered breathing. Oral Maxillofac Surg Clin North Am. 2009;21(4):413-20.	Excluded by abstract
334	Almeida FR, Parker JA, Hodges JS, Lowe AA, Ferguson KA. Effect of a titration polysomnogram on treatment success with a mandibular repositioning appliance. J Clin Sleep Med. 2009;5(3):198-204.	Excluded by abstract
335	Amaral Junior R, Kim LJ, Tufik S, Andersen ML. Is it possible to prevent obstructive sleep apnea with maxillomandibular orthopedic treatment during childhood? Sleep Breath. 2014;18(4):675-6.	Excluded by abstract
336	Amoric M. Efficacy and compliance in treatment of sleep apnea with Herbst mandibular advancement splints (OHA version). Int Orthod. 2013;11(2):193-209.	Excluded by abstract
337	Andren A, Hedberg P, Walker-Engstrom ML, Wahlen P, Tegelberg A. Effects of treatment with oral appliance on 24-h blood pressure in patients with obstructive sleep apnea and hypertension: a randomized clinical trial. Sleep Breath. 2013;17(2):705-12.	Excluded by abstract
338	Anitua E, Duran-Cantolla J, Almeida GZ, Alkhraisat MH. Minimizing the mandibular advancement in an oral appliance for the treatment of obstructive sleep apnea. Sleep Med. 2017;34:226-31.	Excluded by abstract
339	Anon. Dispositivos de avance mandibular (DAM) en el tratamiento del SAHS. Arch bronconeumol (Ed impr). 2005;41(supl.4):68-74.	Excluded by abstract
340	Antosz M. CBCT volumetric analyses have no value in assessing functional airway. Am J Orthod Dentofacial Orthop. 2015;147(1):10-1.	Excluded by abstract
341	Aragao W. Aragao's function regulator, the stomatognathic system and postural changes in children. J Clin Pediatr Dent. 1991;15(4):226-31.	Excluded by abstract
342	Arvystas MG, Wolford LM. Establishing ideal occlusion to achieve esthetic and functional excellence: an interdisciplinary approach to functional esthetics. Compend Contin Educ Dent. 2010;31(4):288-92, 94-6.	Excluded by abstract
343	Arya D, Singh SV, Tripathi A, Tripathi SK. A pilot study to compare patient perception of obstructive sleep apnea treatment with CPAP or appliance therapy. J Prosthet Dent. 2014;112(5):1188-93.	Excluded by abstract
344	Bailey DR. Oral Appliances for Sleep Breathing Disorders. Current Sleep Medicine Reports. 2016;2(2):114-9.	Excluded by abstract
345	Bailey DR. Snoring and sleep apnea: the dentist's role in treatment. Dent Today. 2000;19(11):52-5.	Excluded by abstract
346	Ballanti F, Ranieri S, Baldini A, Cozza P. Long term therapeutic efficacy of a soft monobloc mandibular advancement device in adults with obstructive sleep apnea. ScientificWorldJournal. 2015;2015:408469.	Excluded by abstract
347	Bamagoos A, Sutherland K, Chan A, Leigh M, Ngiam J, Darendeliler MA, et al. Photographic craniofacial analysis and mandibular advancement splint treatment response. Sleep and Biological Rhythms. 2015;13:44.	Excluded by abstract
348	Barewal RM, Hagen CC. Management of snoring and obstructive sleep apnea with mandibular repositioning appliances: A prosthodontic approach. Dental Clinics of North America. 2014;58(1):159-80.	Excluded by abstract
349	Barnes M, McEvoy RD, Banks S, Tarquinio N, Murray CG, Vowles N, et al. Efficacy of positive airway pressure and oral appliance in mild to moderate obstructive sleep apnea. Am J Respir Crit Care Med. 2004;170(6):656-64.	Excluded by abstract
350	Bartolucci ML, Bortolotti F, Raffaelli E, D'Anto V, Michelotti A, Alessandri Bonetti G. The effectiveness of different mandibular advancement amounts in OSA patients: a systematic review and meta-regression analysis. Sleep Breath. 2016;20(3):911-9.	Excluded by abstract
351	Bates CJ, McDonald JP. Patients' and sleeping partners' experience of treatment for sleep-related breathing disorders with a mandibular repositioning splint. Br Dent J. 2006;200(2):95-101; discussion 92.	Excluded by abstract
352	Battagel JM, Johal A, Kotecha BT. Sleep nasendoscopy as a predictor of treatment success in snorers using mandibular advancement splints. J Laryngol Otol. 2005;119(2):106-12.	Excluded by abstract
353	Battagel JM, Kotecha B. Dental side-effects of mandibular advancement splint wear in patients who snore. Clin Otolaryngol. 2005;30(2):149-56.	Excluded by abstract
354	Battagel JM, L'Estrange PR, Nolan P, Harkness B. The role of lateral cephalometric radiography and fluoroscopy in assessing mandibular advancement in sleep-related disorders. Eur J Orthod. 1998;20(2):121-32.	Excluded by abstract
355	Bayat M, Shariati M, Rakhshan V, Abbasi M, Fateh A, Sobouti F, et al. Cephalometric risk factors of obstructive sleep apnea. Cranio-the Journal of Craniomandibular Practice. 2017;35(5):321-6.	Excluded by abstract
356	Becerra N, Firmani M, Valencia E, Cazenave L, Sotomayor C, Espinosa P, et al. Efficiency of the Ocluch©MAD in the treatment of patients with OSAS and its association with craniofacial morphology. Sleep Science. 2018;11(1):12-9.	Excluded by abstract
357	Bender CA, Veneman W, Veenland JF, Mathijssen IM, Hop WC, Koudstaal MJ, et al. Orbital aspects following monobloc advancement in syndromic craniosynostosis. J Craniomaxillofac Surg. 2013;41(7):e146-53.	Excluded by abstract
358	Bender SD. An unusual case of hypnic headache ameliorated utilizing a mandibular advancement oral appliance. Sleep Breath. 2012;16(3):599-602.	Excluded by abstract
359	Bender SD. Oral appliance therapy for sleep-related breathing disorders. Operative Techniques in Otolaryngology - Head and Neck Surgery. 2012;23(1):72-8.	Excluded by abstract
360	Benoist L, de Ruiter M, de Lange J, de Vries N. A randomized, controlled trial of positional therapy versus oral appliance therapy for position-dependent sleep apnea. Sleep Med. 2017;34:109-17.	Excluded by abstract
361	Bhamrah G, Dhir A, Cash A, Ahmad S, Winchester LJ. Patient's experience of treatment for sleep apnoea with a mandibular advancement splint. Surgeon. 2015;13(5):256-62.	Excluded by abstract
362	Bishop B, Verrett R, Girvan T. A randomized crossover study comparing two mandibular repositioning appliances for treatment of obstructive sleep apnea. Sleep Breath. 2014;18(1):125-31.	Excluded by abstract

363	Blanco J, Zamarron C, Abeleira Pazos MT, Lamela C, Suarez Quintanilla D. Prospective evaluation of an oral appliance in the treatment of obstructive sleep apnea syndrome. Sleep Breath. 2005;9(1):20-5.	Excluded by abstract
364	Bloch KE, Iseli A, Zhang JN, Xie X, Kaplan V, Stoeckli PW, et al. A randomized, controlled crossover trial of two oral appliances for sleep apnea treatment. Am J Respir Crit Care Med. 2000;162(1):246-51.	Excluded by abstract
365	Bloch KE. Alternatives to CPAP in the treatment of the obstructive sleep apnea syndrome. Swiss Med Wkly. 2006;136(17-18):261-7.	Excluded by abstract
366	Bonilla CM. Apnea, therapeutic alternatives. Mandibular Advance Device (DAM). Sleep Science. 2019;12:33-4.	Excluded by abstract
367	Bosshard V, Masse JF, Series F. Prediction of oral appliance efficiency in patients with apnoea using phrenic nerve stimulation while awake. Thorax. 2011;66(3):220-5.	Excluded by abstract
368	Boudewyns A, Marklund M, Hochban W. Alternatives for OSAHS treatment: Selection of patients for upper airway surgery and oral appliances. European Respiratory Review. 2007;16(106):132-45.	Excluded by abstract
369	Braem M. In vitro retention of a new thermoplastic titratable mandibular advancement device. F1000Res. 2015;4:56.	Excluded by abstract
370	Brette C, Ramanantsoa H, Renouardiere J, Renouardiere R, Roisman G, Escourrou P. A mandibular advancement device for the treatment of obstructive sleep apnea: long-term use and tolerance. Int Orthod. 2012;10(4):363-76.	Excluded by abstract
371	Caldas SGFR, Ribeiro AA, Santos-Pinto Ld, Martins LP, Matoso RM. Efetividade dos aparelhos intrabucais de avanço mandibular no tratamento do ronco e da síndrome da apneia e hipopneia obstrutiva do sono (SAHOS): revisão sistemática. Rev Dent Press Ortod Ortop Facial (Impr). 2009;14(4):74-82.	Excluded by abstract
372	Caldas SGFR, Ribeiro AA, Santos-Pinto LD, Martins LP, Matoso RM. The effectiveness of mandibular advancement intraoral appliances in the treatment of the snoring and obstructive sleep apnea and hypopnea syndrome (OSAHS): Systematic review. Revista Dental Press de Ortodontia e Ortopedia Facial. 2009;14(4):74-82.	Excluded by abstract
373	Camacho M, Liu SY, Certal V, Capasso R, Powell NB, Riley RW. Large maxillomandibular advancements for obstructive sleep apnea: An operative technique evolved over 30 years. Journal of Cranio-Maxillofacial Surgery. 2015;43(7):1113-8.	Excluded by abstract
374	Campos HS. Distúrbios respiratórios do sono. J bras med. 2012;100(4):27-33.	Excluded by abstract
375	Carlos Villafranca Fd, Cobo Plana J, Macías Escalada E, Díaz Esnal B. Tratamiento de la apnea obstructiva del sueño con posicionadores mandibulares. RCOE, Rev Ilustre Cons Gen Col Odontól Estomatól Esp. 2002;7(4):379-86.	Excluded by abstract
376	Cartwright R. Return of the TRD. J Clin Sleep Med. 2009;5(5):439-40.	Excluded by abstract
377	Chae JM, Chang NY, Cho JH, Kang KH, Kim SC. Treatment of skeletal Class II adult patient with vertical and transverse problems caused by nasal airway obstruction using microimplant anchorage. Korean Journal of Orthodontics. 2009;39(4):257-72.	Excluded by abstract
378	Chan AS, Cistulli PA. Oral appliance treatment of obstructive sleep apnea: an update. Curr Opin Pulm Med. 2009;15(6):591-6.	Excluded by abstract
379	Chan AS, Lee RW, Cistulli PA. Non-positive airway pressure modalities: mandibular advancement devices/positional therapy. Proc Am Thorac Soc. 2008;5(2):179-84.	Excluded by abstract
380	Chate RAC. Do we really want a quick fix? British Dental Journal. 2000;188(4):177-86.	Excluded by abstract
381	Chate RAC. The Burden of Proof - a Critical-Review of Orthodontic Claims Made by Some General-Practitioners. American Journal of Orthodontics and Dentofacial Orthopedics. 1994;106(1):96-105.	Excluded by abstract
382	Chen H, Aarab G, De Lange J, Van Der Stelt P, Lobbezoo F, Darendeliler MA, et al. Differences in three-dimensional craniofacial anatomy between responders and non responders to mandibular advancement splint treatment in obstructive sleep apnea patients. Sleep Medicine. 2017;40:e54.	Excluded by abstract
383	Chen H, Aarab G, de Lange J, van der Stelt P, Lobbezoo F. The Effects of Noncontinuous Positive Airway Pressure Therapies on the Aerodynamic Characteristics of the Upper Airway of Obstructive Sleep Apnea Patients: A Systematic Review. J Oral Maxillofac Surg. 2018;76(7):1559.e1-.e11.	Excluded by abstract
384	Chen H, Aarab G, Liu JW, Yu YL, Guo J, van der Stelt PF, et al. A novel imaging technique to evaluate airflow characteristics in the upper airway of an obstructive sleep apnea patient. Clin Case Rep. 2017;5(7):1084-7.	Excluded by abstract
385	Chen H, Lowe AA, Strauss AM, de Almeida FR, Ueda H, Fleetham JA, et al. Dental changes evaluated with a 3D computer-assisted model analysis after long-term tongue retaining device wear in OSA patients. Sleep Breath. 2008;12(2):169-78.	Excluded by abstract
386	Chen H, Yagi K, Almeida FR, Pliska BT, Lowe AA. Oral appliance treatment for pediatric orthodontic patients with or without sleep problems. Sleep and Breathing. 2012;16(3):926.	Excluded by abstract
387	Chen H, Yagi K, Tsuda H, Almeida F, Lowe A. Klearwa yTM oral appliances for pediatric patients with retruded mandibles. Canadian Respiratory Journal. 2012;19(3):e39.	Excluded by abstract
388	Chung JW, Enciso R, Levendowski DJ, Morgan TD, Westbrook PR, Clark GT. Treatment outcomes of mandibular advancement devices in positional and nonpositional OSA patients. Oral Surg Oral Med Oral Pathol Oral Radiol Endod. 2010;109(5):724-31.	Excluded by abstract
389	Clark GT, Kobayashi H, Freymiller E. Mandibular advancement and sleep disordered breathing. J Calif Dent Assoc. 1996;24(4):49-54, 6-8, 60-1.	Excluded by abstract
390	Cobo Plana J, de Carlos Villafranca F, Macias Escalada E. [Orthodontics and the upper airway]. Orthod Fr. 2004;75(1):31-7.	Excluded by abstract
391	Cohen-Levy J, Petelle B, Pinguet J, Limerat E, Fleury B. Forces created by mandibular advancement devices in OSAS patients: a pilot study during sleep. Sleep Breath. 2013;17(2):781-9.	Excluded by abstract
392	Cohen-Levy J, Potenza J, Couloigner V. [Pediatric obstructive sleep apnea syndrome: Treatment strategy]. Arch Pediatr. 2017;24 Suppl 1:S39-s47.	Excluded by abstract
393	Cohen-Levy J. Orthodontic treatments of pediatric obstructive sleep apnea syndrome. Medecine du Sommeil. 2011;8(2):61-8.	Excluded by abstract
394	Cooke ME, Battagel JM. A thermoplastic mandibular advancement device for the management of non-apnoeic snoring: a randomized controlled trial. Eur J Orthod. 2006;28(4):327-38.	Excluded by abstract
395	Cortes DM, Wallace-Nadolski ME. Non-surgical, upper airway remodeling for skeletal class III and malocclusion with OSA. Sleep. 2017;40:A235-A6.	Excluded by abstract
396	Cozza P, Polimeni A, Ballanti F. A modified monobloc for the treatment of obstructive sleep apnoea in paediatric patients. European Journal of Orthodontics. 2004;26(5):523-30.	Excluded by abstract
397	Crivellin G, Bruno G, De Stefani A, Mazzoli A, Mandolini M, Brunzini A, et al. Strength distribution on TMJ using mandibular advancement device for OSAS treatment: A finite element study. Dental Cadmos. 2018;86(9):757-64.	Excluded by abstract
398	Cuccia AM, Caradonna C. Mandibular advancement devices: indications and predictors of treatment outcome. A review. Minerva Stomatol. 2007;56(9):427-43.	Excluded by abstract
399	Cunali PA, Almeida FR, Santos CD, Valdrichi NY, Nascimento LS, Dal-Fabbro C, et al. Mandibular exercises improve mandibular advancement device therapy for obstructive sleep apnea. Sleep Breath. 2011;15(4):717-27.	Excluded by abstract
400	Currier GF. Nonsurgical and interactive orthodontics. Curr Opin Dent. 1991;1(5):598-608.	Excluded by abstract
401	Daftary AS, Kotagal S. Treatment of childhood obstructive sleep apnea. Current Treatment Options in Neurology. 2010;12(5):369-78.	Excluded by abstract
402	DĂnuťrĂdescu O, AţĂrmure N, Man S, Albu S, BĂciuţ M, Bran S, et al. A case report of pediatric sleep apnea syndrome in an 8-years-old patient: Effects of an anterior mandibular positioning device and rapid palatal expansion. Clujul Medical. 2017;90:S160.	Excluded by abstract
403	David M, Bou Saba S, Liistro G, Rodenstein D, Rombaux P. [Orthodontic appliances in the treatment of sleep apnea: a cephalometric and polysomnographic study]. Bull Group Int Rech Sci Stomatol Odontol. 2000;42(2-3):73-81.	Excluded by abstract
404	de Almeida FR, Lowe AA, Otsuka R, Fastlicht S, Farbood M, Tsuiki S. Long-term sequellae of oral appliance therapy in obstructive sleep apnea patients: Part 2. Study-model analysis. American Journal of Orthodontics and Dentofacial Orthopedics. 2006;129(2):205-13.	Excluded by abstract
405	de Almeida FR, Lowe AA, Sung JO, Tsuiki S, Otsuka R. Long-term sequellae of oral appliance therapy in obstructive sleep apnea patients: Part 1. Cephalometric analysis. American Journal of Orthodontics and Dentofacial Orthopedics. 2006;129(2):195-204.	Excluded by abstract
406	de Britto Teixeira AO, Abi-Ramia LB, de Oliveira Almeida MA. Treatment of obstructive sleep apnea with oral appliances. Prog Orthod. 2013;14:10.	Excluded by abstract
407	de Carlos F, Cobo J, Fernandez Mondragon MP, Alvarez Suarez A, Calvo Blanco J. Orthoimplants: an alternative treatment for SAHS? Sleep Breath. 2010;14(2):171-4.	Excluded by abstract

408	de Carlos Villafranca F, Cobo Plana J, Diaz-Esnal B, Fernandez-Mondragon P, Macias Escalada E, Puente Rodriguez M. [Chronic snoring and obstructive sleep apnea-hypopnea syndrome in children]. Orthod Fr. 2003;74(3):431-57.	Excluded by abstract
409	De Coster T. [Orthopedic expansion of the maxilla]. Orthod Fr. 2006;77(2):253-64.	Excluded by abstract
410	de Lima CMP, Furquim LZ, Ramos AL. Short-term efficacy of mandibular advancement splint in treatment of obstructive sleep apnea-hypopnea syndrome. Dental Press Journal of Orthodontics. 2013;18(3):118-23.	Excluded by abstract
411	De Lourdes Rabelo Guimarães M, Hermont AP, Guimarães TM, Dal-Fabbro C, Bittencourt L, Junior CMC. Severe obstructive sleep apnea treatment with mandibular advancement device: A case report. Sleep Science. 2018;11(2):118-22.	Excluded by abstract
412	de Souza Carvalho AC, Magro Filho O, Garcia IR, Jr., Araujo PM, Nogueira RL. Cephalometric and three-dimensional assessment of superior posterior airway space after maxillomandibular advancement. Int J Oral Maxillofac Surg. 2012;41(9):1102-11.	Excluded by abstract
413	de Vries GE, Wijkstra PJ, Houwerzijl EJ, Kerstjens HAM, Hoekema A. Cardiovascular effects of oral appliance therapy in obstructive sleep apnea: A systematic review and meta-analysis. Sleep Med Rev. 2018;40:55-68.	Excluded by abstract
414	Deane SA, Cistulli PA, Ng AT, Zeng B, Petocz P, Darendeliler MA. Comparison of mandibular advancement splint and tongue stabilizing device in obstructive sleep apnea: a randomized controlled trial. Sleep. 2009;32(5):648-53.	Excluded by abstract
415	Deguchi T. Skeletal, dental, and functional effects of headgear-activator therapy on Class II malocclusion in Japanese: a clinical case report. Am J Orthod Dentofacial Orthop. 1991;100(3):274-85.	Excluded by abstract
416	Denolf PL, Vanderveken OM, Marklund ME, Braem MJ. The status of cephalometry in the prediction of non-CPAP treatment outcome in obstructive sleep apnea patients. Sleep Med Rev. 2016;27:56-73.	Excluded by abstract
417	Dieltjens M, Vanderveken OM, Hamans E, Verbraecken JA, Wouters K, Willemen M, et al. Treatment of obstructive sleep apnea using a custom-made titratable duobloc oral appliance: a prospective clinical study. Sleep Breath. 2013;17(2):565-72.	Excluded by abstract
418	Distel VA, Suntsov VG, Vagner VD, Karnitskaia IV. [A method for preventing and treating maxillodental anomalies related to disordered nose breathing]. Stomatologiia (Mosk). 1998;77(2):53-4.	Excluded by abstract
419	Doff MH, Finnema KJ, Hoekema A, Wijkstra PJ, de Bont LG, Stegenga B. Long-term oral appliance therapy in obstructive sleep apnea syndrome: a controlled study on dental side effects. Clin Oral Investig. 2013;17(2):475-82.	Excluded by abstract
420	Doff MH, Hoekema A, Pruim GJ, Huddleston Slater JJ, Stegenga B. Long-term oral-appliance therapy in obstructive sleep apnea: a cephalometric study of craniofacial changes. J Dent. 2010;38(12):1010-8.	Excluded by abstract
421	Domingos RG, Dolci JEL, Harashima T. Obstructive sleep apnea: clinical results of a case treated with an oral appliance. Braz j otorhinolaryngol (Impr). 2011;77(4):537-.	Excluded by abstract
422	Dort L, Brant R. A randomized, controlled, crossover study of a noncustomized tongue retaining device for sleep disordered breathing. Schlaf & Atmung [Sleep & breathing] [Internet]. 2008; 12(4):[369-73 pp.]. Available from: https://www.cochranelibrary.com/central/doi/10.1002/central/CN-00668422/full.	Excluded by abstract
423	Duarte ER, Frigério MLMA, Tavano O, Razuk PC, Costa MRCM, Martins CHF, et al. Treatment of snoring and sleep apnea syndrome with a removable mandibular advancement device in patients without TMD. Dental Press Journal of Orthodontics. 2012;17(2):90-6.	Excluded by abstract
424	Dunphy L, Sood V, Pilley R, Currie R. Mandibular advancement appliances: Are they effective in the management of Obstructive Sleep Apnoea (OSA)? British Journal of Oral and Maxillofacial Surgery. 2012;50:S40.	Excluded by abstract
425	Durán-Cantolla J, Aizpuru F, Miranda E, Alkhraisat M-H, Anitua E, Crovetto-Martínez R, et al. Efficacy of mandibular advancement device in the treatment of obstructive sleep apnea syndrome: A randomized controlled crossover clinical trial. Med oral patol oral cir bucal (Internet). 2015;20(5):605-15.	Excluded by abstract
426	Eggensperger N, Smolka K, Johner A, Rahal A, Thuer U, Iizuka T. Long-term changes of hyoid bone and pharyngeal airway size following advancement of the mandible. Oral Surg Oral Med Oral Pathol Oral Radiol Endod. 2005;99(4):404-10.	Excluded by abstract
427	El AS, El H, Palomo JM, Baur DA. A 3-dimensional airway analysis of an obstructive sleep apnea surgical correction with cone beam computed tomography. J Oral Maxillofac Surg. 2011;69(9):2424-36.	Excluded by abstract
428	El-Solh AA, Moitheennazima B, Akinnusi ME, Churder PM, Lafornara AM. Combined oral appliance and positive airway pressure therapy for obstructive sleep apnea: a pilot study. Sleep Breath. 2011;15(2):203-8.	Excluded by abstract
429	Esaki K, Kanegae H, Uchida T, Mizuma H, Sakamoto T, Kameyama T. Treatment of sleep apnea with a new separated type of dental appliance (mandibular advancing positioner). Kurume Med J. 1997;44(4):315-9.	Excluded by abstract
430	Fareen N, Alam MK. Recent advances and future opportunities of face mask appliance. International Medical Journal. 2017;24(1):81-6.	Excluded by abstract
431	Farole A, Mundenar MJ, Braitman LE. Posterior airway changes associated with mandibular advancement surgery: implications for patients with obstructive sleep apnea. Int J Adult Orthodon Orthognath Surg. 1990;5(4):255-8.	Excluded by abstract
432	Ferguson KA. Oral appliance therapy for the management of sleep-disordered breathing. Seminars in Respiratory and Critical Care Medicine. 1998;19(2):157-64.	Excluded by abstract
433	Fernandes P, Amaral J, Figueiredo J, Moralejo J, Pita I. Non-invasive ventilation after neuromuscular block antagonism with sugamadex in an asthmatic patient. European journal of anaesthesiology [Internet]. 2013; 30:[91 p.]. Available from: https://www.cochranelibrary.com/central/doi/10.1002/central/CN-01064811/full.	Excluded by abstract
434	Filho RM, Lima AL. Long-term outcome of skeletal Class II Division 1 malocclusion treated with rapid palatal expansion and Kloehn cervical headgear. Am J Orthod Dentofacial Orthop. 2003;124(2):216-24.	Excluded by abstract
435	Flanagan D. A dual-laminate snore reduction appliance: a report of four cases. Gen Dent. 2010;58(4):e156-61.	Excluded by abstract
436	Fleetham JA. Oral appliance treatment for snoring and obstructive sleep apnea/hypopnea. Minerva Pneumologica. 2007;46(3):223-8.	Excluded by abstract
437	Fransson AM, Tegelberg A, Leissner L, Wenneberg B, Isacsson G. Effects of a mandibular protruding device on the sleep of patients with obstructive sleep apnea and snoring problems: a 2-year follow-up. Sleep Breath. 2003;7(3):131-41.	Excluded by abstract
438	Fransson AMC, Kowalczyk A, Isacsson G. A prospective 10-year follow-up dental cast study of patients with obstructive sleep apnoea/snoring who use a mandibular protruding device. European Journal of Orthodontics. 2017;39(5):502-8.	Excluded by abstract
439	Frapier L, Garcia C, Pic E, Morant F, Belguendouz S, Gauthier A, et al. Successful orthodontic-surgical treatment: aiming for esthetics and function. Analysis of some clinical cases. Int Orthod. 2013;11(4):357-88.	Excluded by abstract
440	Friedlander AH, Walker LA, Friedlander IK, Felsenfeld AL. Diagnosing and comanaging patients with obstructive sleep apnea syndrome. J Am Dent Assoc. 2000;131(8):1178-84.	Excluded by abstract
441	Friedman M, Pulver T, Wilson MN, Golbin D, Leesman C, Lee G, et al. Otolaryngology office-based treatment of obstructive sleep apnea-hypopnea syndrome with titratable and nontitratable thermoplastic mandibular advancement devices. Otolaryngol Head Neck Surg. 2010;143(1):78-84.	Excluded by abstract
442	Fritsch KM, Iseli A, Russi EW, Bloch KE. Side effects of mandibular advancement devices for sleep apnea treatment. Am J Respir Crit Care Med. 2001;164(5):813-8.	Excluded by abstract
443	Fukawa A. Two Class II, division 1 patients with congenitally missing lower central incisors. Am J Orthod Dentofacial Orthop. 1993;104(5):425-43.	Excluded by abstract
444	Galang-Boquiren MT, Stache R, Viana MG, Prasad B, Carley D. Oral appliance and pharmacologic agents in treatment of obstructive sleep apnea: A pilot clinical study. Sleep Medicine. 2017;40:e104.	Excluded by abstract
445	Galiano-Castillo N, Ariza-Garcia A, Cantarero-Villanueva I, Fernandez-Lao C, Diaz-Rodriguez L, Legeren-Alvarez M, et al. Telehealth system (e-CUIDATE) to improve quality of life in breast cancer survivors: rationale and study protocol for a randomized clinical trial. Trials [Internet]. 2013; 14(1). Available from: https://www.cochranelibrary.com/central/doi/10.1002/central/CN-00920458/full.	Excluded by abstract
446	Galic T, Bozic J, Pecotic R, Ivkovic N, Valic M, Dogas Z. Improvement of Cognitive and Psychomotor Performance in Patients with Mild to Moderate Obstructive Sleep Apnea Treated with Mandibular Advancement Device: A Prospective 1-Year Study. J Clin Sleep Med. 2016;12(2):177-86.	Excluded by abstract
447	Gao H, Xiao D, Zhao Z. [Effects of Frankel II appliance on sagittal dimensions of upper airway in children]. Hua Xi Kou Qiang Yi Xue Za Zhi. 2003;21(2):116-7.	Excluded by abstract

448	Gao X, Otsuka R, Ono T, Honda E, Sasaki T, Kuroda T. Effect of titrated mandibular advancement and jaw opening on the upper airway in nonapneic men: a magnetic resonance imaging and cephalometric study. Am J Orthod Dentofacial Orthop. 2004;125(2):191-9.	Excluded by abstract
449	Gao XM, Otsuka R, Ono T, Honda E, Sasaki T, Kuroda T. [Changes in shape of upper airway during titrated mandibular advancement: a magnetic resonance imaging study]. Zhonghua Er Bi Yan Hou Tou Jing Wai Ke Za Zhi. 2005;40(2):137-40.	Excluded by abstract
450	Garg RK, Afifi AM, Sanchez R, King TW. Obstructive Sleep Apnea in Adults: The Role of Upper Airway and Facial Skeletal Surgery. Plast Reconstr Surg. 2016;138(4):889-98.	Excluded by abstract
451	Garreau E, Wojcik T, Bouscaillou J, Ferri J, Raoul G. [Comparative effectiveness of maxillomandibular advancement surgery versus mandibular advancement device for patients with moderate or severe obstructive sleep area]. Orthod Fr. 2014;85(2):163-73.	Excluded by abstract
452	Giannasi LC, Almeida FR, Magini M, Costa MS, de Oliveira CS, de Oliveira JC, et al. Systematic assessment of the impact of oral appliance therapy on the temporomandibular joint during treatment of obstructive sleep apnea: long-term evaluation. Sleep Breath. 2009;13(4):375-81.	Excluded by abstract
453	Giannasi LC, Almeida FR, Nacif SR, de Oliveira LV. Efficacy of an oral appliance for the treatment of obstructive sleep apnea. Int J Prosthodont. 2013;26(4):334-9.	Excluded by abstract
454	Giannasi LC, Almeida RC, Oliveira LVF. A markedly retrognathic and severe osa patient treated with an oral appliance: 3 years follow-up. Sleep Medicine. 2009;10:S69.	Excluded by abstract
455	Gibbs T. Classification of mandibular advancement appliances. Sleep and Biological Rhythms. 2014;12:31.	Excluded by abstract
456	Gindre L, Gagnadoux F, Meslier N, Fleury B, Gustin JM, Racineux JL. [Central apnea developing during treatment with a mandibular advancement device]. Rev Mal Respir. 2006;23(5 Pt 1):477-80.	Excluded by abstract
457	Godoy LBM, Palombini L, Poyares D, Dal-Fabbro C, Guimaraes TM, Klichouvicz PC, et al. Long-Term Oral Appliance Therapy Improves Daytime Function and Mood in Upper Airway Resistance Syndrome Patients. Sleep. 2017;40(12).	Excluded by abstract
458	Gomes MS. MSG – Sound Sleep – Aparelho intraoral auxiliar na terapia do ronco e síndrome da apneia e hipopneia obstrutiva do sono (SAHOS). Ortho Sci, Orthod sci pract. 2015;8(31):423-34.	Excluded by abstract
459	Goncalves JR, Gomes LC, Vianna AP, Rodrigues DB, Goncalves DA, Wolford LM. Airway space changes after maxillomandibular counterclockwise rotation and mandibular advancement with TMJ Concepts(R) total joint prostheses: three-dimensional assessment. Int J Oral Maxillofac Surg. 2013;42(8):1014-22.	Excluded by abstract
460	Gong X, Zhao Y, Li WR, Gao XM. [Efficacy of oral appliance therapy of obstructive sleep apnea and hypopnea syndrome in different periods of treatment]. Beijing Da Xue Xue Bao Yi Xue Ban. 2017;49(1):115-9.	Excluded by abstract
461	Gonzalez M, Macias-Escalada E, Cobo J, Fernandez Mondragon MP, Gomez-Moreno G, Martinez-Martinez M, et al. Can treatment with statins have a negative influence on the tolerance of mandibular advancement devices? Sleep Breath. 2016;20(4):1363-6.	Excluded by abstract
462	González Mangado N, Troncoso Acevedo MF, Gómez García T. Home Ventilation Therapy in Obstructive Sleep Apnea–Hypopnea Syndrome. Archivos de Bronconeumologia. 2014;50(12):528-34.	Excluded by abstract
463	Graber TM. The use of muscle forces by simple orthodontic appliances. Am J Orthod. 1979;76(1):1-20.	Excluded by abstract
464	Guilleminault C, Quo SD. Sleep-disordered breathing. A view at the beginning of the new Millennium. Dent Clin North Am. 2001;45(4):643-56.	Excluded by abstract
465	Guimarães MdLR, Pereira JBB, Jardim FFT, Costa TMMd, Becattini PR, Hermont AP. Efetividade em Longo-Prazo de dois Aparelhos Intraorais no Tratamento da Apneia Obstrutiva do Sono: Relato de um Caso. UNOPAR Cient, Ciênc biol saude. 2014;16(4).	Excluded by abstract
466	Gulati A, Chate RA, Howes TQ. Can a single cephalometric measurement predict obstructive sleep apnea severity? J Clin Sleep Med. 2010;6(1):64-8.	Excluded by abstract
467	Gupta A, Tripathi A, Sharma P. The long-term effects of mandibular advancement splint on cardiovascular fitness and psychomotor performance in patients with mild to moderate obstructive sleep apnea: a prospective study. Sleep Breath. 2017;21(3):781-9.	Excluded by abstract
468	Guttal SS, Patil NP, Mantur SS. Conservative treatment of obstructive sleep apnea: a case report. N Y State Dent J. 2008;74(5):57-9.	Excluded by abstract
469	Haviv Y, Kamer L, Sheinfeld R, Almoznino G, Bachar G. Successful Treatment of Extremely Severe Obstructive Sleep Apnea with a Dental Appliance. Isr Med Assoc J. 2018;20(7):429-32.	Excluded by abstract
470	Heike CL, Avellino AM, Mirza SK, Kifle Y, Perkins J, Sze R, et al. Sleep disturbances in 22q11.2 deletion syndrome: a case with obstructive and central sleep apnea. Cleft Palate Craniofac J. 2007;44(3):340-6.	Excluded by abstract
471	Hein H, Betge S, Gross DH, Magnussen H. Effects, Side Effects and Quality of Life Associated with Treatment of Sleep-Disordered Breathing Using a Functional Appliance - A Modified Activator (Bionator). Somnologie. 2004;8(2):46-52.	Excluded by abstract
472	Hernández Felipe O, Razón Behar R, Barrera Miclín R. Nueva pantalla vestibular. Su influencia en el tratamiento de las desarmonias dento-maxilofaciales. Rev cuba ortod. 1996;11(1):35-44.	Excluded by abstract
473	Hochban W, Hoch B. [Obstructive sleep apnea in the child: an interdisciplinary treatment concept with special reference to craniofacial changes]. Pneumologie. 1998;52(3):147-53.	Excluded by abstract
474	Hoekema A, de Lange J, Stegenga B, de Bont LG. Oral appliances and maxillomandibular advancement surgery: an alternative treatment protocol for the obstructive sleep apnea-hypopnea syndrome. J Oral Maxillofac Surg. 2006;64(6):886-91.	Excluded by abstract
475	Hoekema A, de Vries F, Heydenrijk K, Stegenga B. Implant-retained oral appliances: a novel treatment for edentulous patients with obstructive sleep apnea-hypopnea syndrome. Clin Oral Implants Res. 2007;18(3):383-7.	Excluded by abstract
476	Hoekema A, Doff MHJ, de Bont LGM, van der Hoeven JH, Wijkstra PJ, Pasma HR, et al. Predictors of obstructive sleep apnea-hypopnea treatment outcome. Journal of Dental Research. 2007;86(12):1181-6.	Excluded by abstract
477	Hoekema A, Stegenga B, Bakker M, Brouwer WH, de Bont LG, Wijkstra PJ, et al. Simulated driving in obstructive sleep apnoea-hypopnoea; effects of oral appliances and continuous positive airway pressure. Sleep Breath. 2007;11(3):129-38.	Excluded by abstract
478	Hoekema A, Stegenga B, De Bont LG. Efficacy and co-morbidity of oral appliances in the treatment of obstructive sleep apnea-hypopnea: a systematic review. Crit Rev Oral Biol Med. 2004;15(3):137-55.	Excluded by abstract
479	Hoekema A, Stegenga B, Wijkstra PJ, van der Hoeven JH, Meinesz AF, de Bont LG. Obstructive sleep apnea therapy. J Dent Res. 2008;87(9):882-7.	Excluded by abstract
480	Hoekema A, Voors AA, Wijkstra PJ, Stegenga B, van der Hoeven JH, Tol CG, et al. Effects of oral appliances and CPAP on the left ventricle and natriuretic peptides. Int J Cardiol. 2008;128(2):232-9.	Excluded by abstract
481	Holley AB, Lettieri CJ, Shah AA. Efficacy of an adjustable oral appliance and comparison with continuous positive airway pressure for the treatment of obstructive sleep apnea syndrome. Chest. 2011;140(6):1511-6.	Excluded by abstract
482	Hong Y, Gu X, Feng X, Wang Y. Modified coronoid process grafts combined with sagittal split osteotomy for treatment of bilateral temporomandibular joint ankylosis. J Oral Maxillofac Surg. 2002;60(1):11-8; discussion 8-9.	Excluded by abstract
483	Horihata A, Ueda H, Koh M, Watanabe G, Tanne K. Enhanced increase in pharyngeal airway size in Japanese class II children following a 1-year treatment with an activator appliance. Int J Orthod Milwaukee. 2013;24(4):35-40.	Excluded by abstract
484	Hourfar J, Kinzinger G, Lisson JA. The Relevance of the Posterior Airway Space for Orthodontics. Informationen Aus Orthodontie Und Kieferorthopaedie. 2020;52(2):98-104.	Excluded by abstract
485	Hoyer H, Limbrock GJ. Orofacial regulation therapy in children with Down syndrome, using the methods and appliances of Castillo-Morales. ASDC J Dent Child. 1990;57(6):442-4.	Excluded by abstract
486	Hsieh YJ, Liao YF, Chen NH, Chen YR. Changes in the calibre of the upper airway and the surrounding structures after maxillomandibular advancement for obstructive sleep apnoea. Br J Oral Maxillofac Surg. 2014;52(5):445-51.	Excluded by abstract
487	Hsieh YJ, Liao YF. Effects of maxillomandibular advancement on the upper airway and surrounding structures in patients with obstructive sleep apnoea: a systematic review. Br J Oral Maxillofac Surg. 2013;51(8):834-40.	Excluded by abstract
488	Huang M, Tan G, Lu S, Huang D, Fu L. Evaluation of effect for obstructive sleep apnea patients treated with soft-hard plastic mandibular advancing oral appliance. Sleep Medicine. 2017;40:e137.	Excluded by abstract
489	Huang MF. Detection for judging method to move mandible forward in the middle. Sleep and Biological Rhythms. 2011;9(4):289-90.	Excluded by abstract
490	Huang YS, Chuang LC, Hervy-Auboiron M, Paiva T, Lin CH, Guilleminault C. Neutral supporting mandibular advancement device with tongue bead for passive myofunctional therapy: a long term follow-up study. Sleep Med. 2018.	Excluded by abstract

491	Huang YS, Guilleminault C. Pediatric Obstructive Sleep Apnea: Where Do We Stand? Adv Otorhinolaryngol. 2017;80:136-44.	Excluded by abstract
492	Hugentobler M, Scolozzi P. Severe dental malocclusion: a rare and insidious complication of mandibular advancement devices for obstructive sleep apnea syndrome treatment. Oral Surg Oral Med Oral Pathol Oral Radiol Endod. 2010;109(3):e28-32.	Excluded by abstract
493	Huynh NT, Desplats E, Almeida FR. Orthodontics treatments for managing obstructive sleep apnea syndrome in children: A systematic review and meta-analysis. Sleep Med Rev. 2016;25:84-94.	Excluded by abstract
494	Huynh NT. Mandibular advancement splints decrease blood pressure similarly as with continuous positive airway pressure in sleep apnea. Oral Dis. 2016;22(5):348-9.	Excluded by abstract
495	Idris G, Galland B, Robertson CJ, Farella M. Efficacy of a Mandibular Advancement Appliance on Sleep Disordered Breathing in Children: A Study Protocol of a Crossover Randomized Controlled Trial. Front Physiol. 2016;7:353.	Excluded by abstract
496	Idris G, Galland B, Robertson CJ, Gray A, Farella M. Mandibular advancement appliances for sleep-disordered breathing in children: A randomized crossover clinical trial. J Dent. 2018;71:9-17.	Excluded by abstract
497	Ierardo G, Luzzi V, Polimeni A. Obstructive Sleep Apnea Syndrome (OSAS): evaluation and treatment of odontostomatological problems. Med Lav. 2017;108(4):293-6.	Excluded by abstract
498	Ihara K, Ogawa T, Shigeta Y, Kawamura N, Mizuno Y, Ando E, et al. The development and clinical application of novel connectors for oral appliance. J Prosthodont Res. 2011;55(3):184-8.	Excluded by abstract
499	Isacsson G, Fodor C, Sturebrand M. Obstructive sleep apnea treated with custom-made bibloc and monobloc oral appliances: a retrospective comparative study. Sleep Breath. 2017;21(1):93-100.	Excluded by abstract
500	Isacsson G, Nohlert E, Fransson A, Wiman Eriksson E, Ortlieb E, Fodor C, et al. Bibloc and monobloc oral appliances in the treatment of obstructive sleep apnoea: A multicenter, randomized, blinded, parallel-group trial. Sleep Medicine. 2017;40:e142-e3.	Excluded by abstract
501	Ishihara Y, Sugawara Y, Ei Hsu Hlaing E, Nasu M, Kataoka T, Odagaki N, et al. Orthodontic correction of severe Class II malocclusion in a patient with Prader-Willi syndrome. Am J Orthod Dentofacial Orthop. 2018;154(5):718-32.	Excluded by abstract
502	Itasaka Y, Miyazaki S, Tada H, Ishikawa K, Togawa K. Effectiveness of prosthetic mandibular advancement for obstructive sleep apnea: analysis by sleep position. Psychiatry Clin Neurosci. 1998;52(2):225-7.	Excluded by abstract
503	Ito S, Otake H, Tsuiki S, Miyao E, Noda A. Obstructive sleep apnea syndrome in a pubescent boy of short stature was improved with an orthodontic mandibular advancement oral appliance: a case report. J Clin Sleep Med. 2015;11(1):75-6.	Excluded by abstract
504	Itzhaki S, Dorchin H, Clark G, Lavie L, Lavie P, Pillar G. The effects of 1-year treatment with a herbst mandibular advancement splint on obstructive sleep apnea, oxidative stress, and endothelial function. Chest. 2007;131(3):740-9.	Excluded by abstract
505	Izci B, McDonald JP, Coleman EL, Mackay TW, Douglas NJ, Engleman HM. Clinical audit of subjects with snoring & sleep apnoea/hypopnoea syndrome fitted with mandibular repositioning splint. Respir Med. 2005;99(3):337-46.	Excluded by abstract
506	Jafari B, Roux F. Non-positive airway pressure therapy for obstructive sleep apnea. Current Respiratory Care Reports. 2012;1(2):111-22.	Excluded by abstract
507	Jagielak M, Wojtowicz A, Rogus P, Mocha M, Szalwinski M. Wing genioplasty by piezo saw-major improvement in the treatment of OSAS. International Journal of Oral and Maxillofacial Surgery. 2013;42(10):1330.	Excluded by abstract
508	Jahnig A, Krysewski R. The Goettingen "Pro-Stab" removable plate system. A retrospective cephalometric study of the effects of a new Class II treatment appliance. J Orofac Orthop. 1997;58(6):320-9.	Excluded by abstract
509	Jaiswal M, Srivastava GN, Pratap CB, Sharma VK, Chaturvedi TP. Effect of Oral Appliance for Snoring and Obstructive Sleep Apnea. Int J Orthod Milwaukee. 2015;26(3):67-71.	Excluded by abstract
510	Jalbert F, Lacassagne L, Bessard J, Dekeister C, Paoli JR, Tiberge M. Oral appliances or maxillomandibular advancement osteotomy for severe obstructive sleep apnoea in patients refusing CPAP. Revue de Stomatologie et de Chirurgie Maxillo-Faciale. 2012;113(1):19-26.	Excluded by abstract
511	Jia PZ, Fu MK, Zeng XL. [Treatment of OSAS with modified twin-block advancement appliances]. Zhonghua Kou Qiang Yi Xue Za Zhi. 2005;40(1):42-5.	Excluded by abstract
512	Johal A, Arya D, Winchester LJ, Venn PJ, Brooks H. The effect of a mandibular advancement splint in subjects with sleep-related breathing disorders. Br Dent J. 2005;199(9):591-6; discussion 81; quiz 608.	Excluded by abstract
513	Johal A, Battagel J, Hector M. Controlled, prospective trial of psychosocial function before and after mandibular advancement splint therapy. Am J Orthod Dentofacial Orthop. 2011;139(5):581-7.	Excluded by abstract
514	Johal A, Battagel JM, Kotecha BT. Sleep nasendoscopy: a diagnostic tool for predicting treatment success with mandibular advancement splints in obstructive sleep apnoea. Eur J Orthod. 2005;27(6):607-14.	Excluded by abstract
515	Johal A, Gill G, Ferman A, McLaughlin K. The effect of mandibular advancement appliances on awake upper airway and masticatory muscle activity in patients with obstructive sleep apnoea. Clin Physiol Funct Imaging. 2007;27(1):47-53.	Excluded by abstract
516	Johal A. Health-related quality of life in patients with sleep-disordered breathing: effect of mandibular advancement appliances. J Prosthet Dent. 2006;96(4):298-302.	Excluded by abstract
517	Johnston C, Gleadhill I, Cinnamond M, Gabbey J, Burden D. Mandibular advancement appliances and obstructive sleep apnoea: a randomized clinical trial. European journal of orthodontics [Internet]. 2002; 24(3):[251-62 pp.]. Available from: https://www.cochranelibrary.com/central/doi/10.1002/central/CN-00395067/full.	Excluded by abstract
518	Jones R, Badlani J, Jones C. Maxillary, mandibular and chin advancement surgery for the treatment of obstructive sleep apnoea. Aust Dent J. 2010;55(3):314-21.	Excluded by abstract
519	Kalladka M. Dental sleep medicine. Sleep and Vigilance. 2017;1(2):137-8.	Excluded by abstract
520	Kannan A, Sathyanarayana HP, Padmanabhan S. Effect of functional appliances on the airway dimensions in patients with skeletal class II malocclusion: A systematic review. J Orthod Sci. 2017;6(2):54-64.	Excluded by abstract
521	Kaur H, Uludag H, Dederich D, El-Bialy T. Dose dependent effect of low intensity pulsed ultrasound on the condylar growth during functional appliance treatment. Journal of Bone and Mineral Research. 2013;28.	Excluded by abstract
522	Kim JS, Kim JK, Hong SC, Cho JH. Changes in the upper airway after counterclockwise maxillomandibular advancement in young Korean women with class II malocclusion deformity. J Oral Maxillofac Surg. 2013;71(9):1603.e1-6.	Excluded by abstract
523	Kim KB. How has our interest in the airway changed over 100 years? American Journal of Orthodontics and Dentofacial Orthopedics. 2015;148(5):740-7.	Excluded by abstract
524	Kim T, Kim HH, Hong S, Baek SH, Kim KW, Suh SH, et al. Change in the Upper Airway of Patients With Obstructive Sleep Apnea Syndrome Using Computational Fluid Dynamics Analysis: Conventional Maxillomandibular Advancement Versus Modified Maxillomandibular Advancement With Anterior Segmental Setback Osteotomy. J Craniofac Surg. 2015;26(8):e765-70.	Excluded by abstract
525	Kim YK, Kim JW, Yoon IY, Rhee CS, Lee CH, Yun PY. Influencing factors on the effect of mandibular advancement device in obstructive sleep apnea patients: analysis on cephalometric and polysomnographic parameters. Sleep Breath. 2014;18(2):305-11.	Excluded by abstract
526	Kisnisci RS. Differential diagnosis of obstructive sleep apnea for the management by skeletal surgery. International Journal of Oral and Maxillofacial Surgery. 2011;40(10):1019.	Excluded by abstract
527	Knappe SW, Bakke M, Svanholt P, Petersson A, Sonnesen L. Long-term side effects on the temporomandibular joints and oro-facial function in patients with obstructive sleep apnoea treated with a mandibular advancement device. J Oral Rehabil. 2017;44(5):354-62.	Excluded by abstract
528	Knappe SW, Sonnesen L. Mandibular positioning techniques to improve sleep quality in patients with obstructive sleep apnea: current perspectives. Nat Sci Sleep. 2018;10:65-72.	Excluded by abstract
529	Kochar GD, Chakranarayan A, Kohli S, Kohli VS, Khanna V, Jayan B, et al. Effect of surgical mandibular advancement on pharyngeal airway dimensions: a three-dimensional computed tomography study. Int J Oral Maxillofac Surg. 2016;45(5):553-9.	Excluded by abstract
530	Kochar GD, Londhe S, Jayan B, Chopra SS, Kohli S, Verma M, et al. Management of Tooth Size Arch Size Discrepancy (TSASD) in Patients with Sleep Disorder Breathing. Int J Orthod Milwaukee. 2016;27(2):9-13.	Excluded by abstract
531	Kochar GD, Sharma M, Roy Chowdhury SK, Londhe SM, Kumar P, Jain A, et al. Pharyngeal airway evaluation following isolated surgical mandibular advancement: A 1-year follow-up. Am J Orthod Dentofacial Orthop. 2019;155(2):207-15.	Excluded by abstract
532	Kochel J, Meyer-Marcotty P, Sickel F, Lindorf H, Stellzig-Eisenhauer A. Short-term pharyngeal airway changes after mandibular advancement surgery in adult Class II-Patients--a three-dimensional retrospective study. J Orofac Orthop. 2013;74(2):137-52.	Excluded by abstract

533	Köhler JFW, Bradasch VL. O papel do ortodontista na apnéia obstrutiva noturna. Dens(Curitiba). 1994;10:19-28.	Excluded by abstract
534	Krekmanov L, Andersson L, Ringqvist M, Wilhelmsson B, Walker-Engstrom ML, Tegelberg A, et al. Anterior-inferior mandibular osteotomy in treatment of obstructive sleep apnea syndrome. Int J Adult Orthodon Orthognath Surg. 1998;13(4):289-98.	Excluded by abstract
535	Krekmanov L. Orthognathic surgery without the use of postoperative intermaxillary fixation. A clinical and cephalometric evaluation of surgical correction of mandibular and maxillary deformities. Swed Dent J Suppl. 1989;61:8-62.	Excluded by abstract
536	Kucukkeles N, Arun T. Bio-thermal Herbst application during the mixed dentition period. J Clin Pediatr Dent. 1994;18(4):253-8.	Excluded by abstract
537	Kumari P, Roy SK, Roy ID, Rajput AK, Prasanna Kumar MP, Datana S, et al. Changes in posterior airway space and mandibular plane hyoid distance following mandibular advancement DO. Ann Maxillofac Surg. 2016;6(2):182-9.	Excluded by abstract
538	Kurtulmus H, Cotert HS. Management of obstructive sleep apnea in an edentulous patient with a combination of mandibular advancement splint and tongue-retaining device: a clinical report. Sleep Breath. 2009;13(1):97-102.	Excluded by abstract
539	Kuzniar TJ, Kovacevic-Ristanovic R, Freedom T. Complex sleep apnea unmasked by the use of a mandibular advancement device. Sleep Breath. 2011;15(2):249-52.	Excluded by abstract
540	Lahey IET, Lee SH, Kaban LB. Skeletal stability of patients undergoing maxillomandibular advancement for treatment of obstructive sleep apnea. Journal of Oral and Maxillofacial Surgery. 2014;72(9):e25-e6.	Excluded by abstract
541	Lam B, Sam K, Lam JC, Lai AY, Lam CL, Ip MS. The efficacy of oral appliances in the treatment of severe obstructive sleep apnea. Sleep Breath. 2011;15(2):195-201.	Excluded by abstract
542	Lamont J, Baldwin DR, Hay KD, Veale AG. Effect of two types of mandibular advancement splints on snoring and obstructive sleep apnoea. Eur J Orthod. 1998;20(3):293-7.	Excluded by abstract
543	Landry ML, Rompre PH, Manzini C, Guitard F, de Grandmont P, Lavigne GJ. Reduction of sleep bruxism using a mandibular advancement device: an experimental controlled study. Int J Prosthodont. 2006;19(6):549-56.	Excluded by abstract
544	Laspos C, Metaxas E, Nicolaou Z. The use of Mandibular Advancement Devices for Treatment of Obstructive Sleep Apnea. Informationen Aus Orthodontie Und Kieferorthopaedie. 2016;48(4):249-53.	Excluded by abstract
545	Lavigne GJ, Herrero Babiloni A, Beetz G, Dal Fabbro C, Sutherland K, Huynh N, et al. Critical Issues in Dental and Medical Management of Obstructive Sleep Apnea. Journal of Dental Research. 2020;99(1):26-35.	Excluded by abstract
546	Lawton H, Battagel J, Kotecha B. A comparison of the Twin Block and Herbst mandibular advancement splints in the treatment of patients with obstructive sleep apnoea: a prospective study. European journal of orthodontics [Internet]. 2005; 27(1):[82-90 pp.]. Available from: https://www.cochranelibrary.com/central/doi/10.1002/central/CN-00502515/full.	Excluded by abstract
547	Lazard DS, Blumen M, Levy P, Chauvin P, Fragny D, Buchet I, et al. The tongue-retaining device: efficacy and side effects in obstructive sleep apnea syndrome. J Clin Sleep Med. 2009;5(5):431-8.	Excluded by abstract
548	Lee CH, Mo JH, Choi IJ, Lee HJ, Seo BS, Kim DY, et al. The mandibular advancement device and patient selection in the treatment of obstructive sleep apnea. Arch Otolaryngol Head Neck Surg. 2009;135(5):439-44.	Excluded by abstract
549	Lee SH, Kaban LB, Lahey ET. Skeletal stability of patients undergoing maxillomandibular advancement for treatment of obstructive sleep apnea. Journal of Oral and Maxillofacial Surgery. 2015;73(4):694-700.	Excluded by abstract
550	Leite FG, Rodrigues RC, Ribeiro RF, Eckeli AL, Regalo SC, Sousa LG, et al. The use of a mandibular repositioning device for obstructive sleep apnea. Eur Arch Otorhinolaryngol. 2014;271(5):1023-9.	Excluded by abstract
551	Lekerud A, Sand L, Englund A, Hirsch J. Treatment of sleep apnoea using a mandibular advancement splint--an open prospective study. In vivo (athens, greece) [Internet]. 2012; 26(5):[841-5 pp.]. Available from: https://www.cochranelibrary.com/central/doi/10.1002/central/CN-00967866/full.	Excluded by abstract
552	Lentini-Oliveira D, Carvalho F, Rodrigues C, Ye Q, Hu R, Minami-Sugaya H, et al. Orthodontic and orthopaedic treatment for anterior open bite in children. Cochrane Database of Systematic Reviews [Internet]. 2014; (9). Available from: http://dx.doi.org/10.1002/14651858.CD005515.pub3.	Excluded by abstract
553	Lettieri CJ, Paolino N, Eliasson AH, Shah AA, Holley AB. Comparison of adjustable and fixed oral appliances for the treatment of obstructive sleep apnea. J Clin Sleep Med. 2011;7(5):439-45.	Excluded by abstract
554	Levendowski DJ, Morgan TD, Patrickus JE, Westbrook PR, Berka C, Zavora T, et al. In-home evaluation of efficacy and titration of a mandibular advancement device for obstructive sleep apnea. Sleep Breath. 2007;11(3):139-47.	Excluded by abstract
555	Li KK. Maxillomandibular advancement for obstructive sleep apnea. J Oral Maxillofac Surg. 2011;69(3):687-94.	Excluded by abstract
556	Li W, Sofi MH, Yeh N, Sehra S, McCarthy BP, Patel DR, et al. Thymic selection pathway regulates the effector function of CD4 T cells. J Exp Med. 2007;204(9):2145-57.	Excluded by abstract
557	Li X, Zhou HL, Lou XT, Hu Z, Shen G. [Effect of functional appliance on upper airway in adolescent patients with skeletal Class II malocclusion]. Shanghai Kou Qiang Yi Xue. 2017;26(2):222-7.	Excluded by abstract
558	Liao YF, Chiu YT, Lin CH, Chen YA, Chen NH, Chen YR. Modified maxillomandibular advancement for obstructive sleep apnoea: Towards a better outcome for Asians. International Journal of Oral and Maxillofacial Surgery. 2015;44(2):189-94.	Excluded by abstract
559	Lim J, Lasserson T, Fleetham J, Wright J. Oral appliances for obstructive sleep apnoea. Cochrane Database of Systematic Reviews [Internet]. 2006; (1). Available from: http://dx.doi.org/10.1002/14651858.CD004435.pub3.	Excluded by abstract
560	Limme M. [Orthodontic treatment in mouth breathing]. Acta Otorhinolaryngol Belg. 1993;47(2):263-71.	Excluded by abstract
561	Lin CH, Liao YF, Chen NH, Lo LJ, Chen YR. Three-dimensional computed tomography in obstructive sleep apneics treated by maxillomandibular advancement. Laryngoscope. 2011;121(6):1336-47.	Excluded by abstract
562	Lindman R, Bondemark L. A review of oral devices in the treatment of habitual snoring and obstructive sleep apnoea. Swed Dent J. 2001;25(1):39-51.	Excluded by abstract
563	Liu CY, Lu HY, Dong FS, Ma WS, Wang J, Hu XY, et al. Effects of a mandibular advancement device on genioglossus in obstructive sleep apnoea hypopnea syndrome. Eur J Orthod. 2015;37(3):290-6.	Excluded by abstract
564	Liu SR, Yi HL, Guan J, Chen B, Wu HM, Yin SK. Changes in facial appearance after maxillomandibular advancement for severe obstructive sleep apnoea hypopnoea syndrome in Chinese patients: a subjective and objective evaluation. Int J Oral Maxillofac Surg. 2012;41(9):1112-9.	Excluded by abstract
565	Liu Y, Lowe AA, Fleetham JA, Park YC. Cephalometric and physiologic predictors of the efficacy of an adjustable oral appliance for treating obstructive sleep apnea. Am J Orthod Dentofacial Orthop. 2001;120(6):639-47.	Excluded by abstract
566	Liu YH, Zhao XG, Xu BF, Li J, Lu MX. [Computer-aided mandibular repositioning system for efficacy prediction of oral appliance in patients with obstructive sleep apnea-hypopnea syndrome]. Zhonghua Kou Qiang Yi Xue Za Zhi. 2006;41(2):86-9.	Excluded by abstract
567	Lockerman LZ. Oral appliance management of obstructive sleep apnea: a case report. J Mass Dent Soc. 2006;55(2):18-20.	Excluded by abstract
568	Loudon M. The Loudon Chateau repositioning appliance. Int J Orthod Milwaukee. 2014;25(3):23-9.	Excluded by abstract
569	Louro RS, Calasans-Maia JA, Mattos CT, Masterson D, Calasans-Maia MD, Maia LC. Three-dimensional changes to the upper airway after maxillomandibular advancement with counterclockwise rotation: a systematic review and meta-analysis. Int J Oral Maxillofac Surg. 2018;47(5):622-9.	Excluded by abstract
570	Lowe AA. Dentistry's role in sleep-disordered breathing. Cranio. 2013;31(2):74-6.	Excluded by abstract
571	Lowe AA. Principles of oral appliance therapy for the management of sleep disordered breathing. Oral and Maxillofacial Surgery Clinics of North America. 2002;14(3):305-17.	Excluded by abstract
572	Lu HY, Dong F, Liu CY, Wang J, Liu Y, Xiao W. An animal model of obstructive sleep apnoea-hypopnea syndrome corrected by mandibular advancement device. Eur J Orthod. 2015;37(3):284-9.	Excluded by abstract
573	Lu HY, Wang W, Zhou Z, Liu CY, Liu Y, Xiao W, et al. Treatment of obstructive sleep apnoea-hypopnea syndrome by mandible advanced device reduced neuron apoptosis in frontal cortex of rabbits. Eur J Orthod. 2018;40(3):273-80.	Excluded by abstract
574	Luzzi V, Ierardo G, Di Carlo G, Saccucci M, Polimeni A. Obstructive sleep apnea syndrome in the pediatric age: the role of the dentist. Eur Rev Med Pharmacol Sci. 2019;23(1 Suppl):9-14.	Excluded by abstract

575	Ma SY, Whittle T, Descallar J, Murray GM, Darendeliler MA, Cistulli P, et al. Association between resting jaw muscle electromyographic activity and mandibular advancement splint outcome in patients with obstructive sleep apnea. Am J Orthod Dentofacial Orthop. 2013;144(3):357-67.	Excluded by abstract
576	Ma YY, Zhang JJ, Gao XM. [Treatment outcome evaluation of different mandibular advancements using oral appliance to treat obstructive sleep apnea and hyponea syndrome: a systematic review]. Beijing Da Xue Xue Bao Yi Xue Ban. 2017;49(4):691-8.	Excluded by abstract
577	Machado MA, Juliano L, Taga M, de Carvalho LB, do Prado LB, do Prado GF. Titratable mandibular repositioner appliances for obstructive sleep apnea syndrome: are they an option? Sleep Breath. 2007;11(4):225-31.	Excluded by abstract
578	Machado-Júnior A-J, Signorelli L-G, Zancanella E, Crespo A-N. Randomized controlled study of a mandibular advancement appliance for the treatment of obstructive sleep apnea in children: A pilot study. Med oral patol oral cir bucal (Internet). 2016;21(4):e403-e7.	Excluded by abstract
579	Machado-Júnior A-J, Zancanella E, Crespo A-N. Rapid maxillary expansion and obstructive sleep apnea: A review and meta-analysis. Med oral patol oral cir bucal (Internet). 2016;21(4):e465-e9.	Excluded by abstract
580	Maganzini AL, Alhussain IY. Treatment of obstructive sleep apnea with combined orthognathic-orthodontic approach assessed by nocturnal polysomnography. N Y State Dent J. 2008;74(3):36-40.	Excluded by abstract
581	Maia S, Raveli DB, Santos-Pinto Ad, Raveli TB, Gomez SP. Avaliação tomográfica no tratamento com Herbst em adulto jovem. Dental press j orthod (Impr). 2010;15(5):130-6.	Excluded by abstract
582	Marchese-Ragona R, Manfredini D, Mion M, Vianello A, Staffieri A, Guarda-Nardini L. Oral appliances for the treatment of obstructive sleep apnea in patients with low C-PAP compliance: a long-term case series. Cranio. 2014;32(4):254-9.	Excluded by abstract
583	Marklund M, Carlberg B, Forsgren L, Olsson T, Stenlund H, Franklin K. Oral Appliance Therapy in Patients With Daytime Sleepiness and Snoring or Mild to Moderate Sleep Apnea: a Randomized Clinical Trial. JAMA internal medicine [Internet]. 2015; 175(8):[1278-85 pp.]. Available from: https://www.cochranelibrary.com/central/doi/10.1002/central/CN-01076679/full.	Excluded by abstract
584	Marklund M, Franklin KA, Persson M. Orthodontic side-effects of mandibular advancement devices during treatment of snoring and sleep apnoea. European Journal of Orthodontics. 2001;23(2):135-44.	Excluded by abstract
585	Marklund M, Franklin KA, Sahlin C, Lundgren R. The effect of a mandibular advancement device on apneas and sleep in patients with obstructive sleep apnea. Chest. 1998;113(3):707-13.	Excluded by abstract
586	Marklund M, Franklin KA. Treatment of elderly patients with snoring and obstructive sleep apnea using a mandibular advancement device. Sleep Breath. 2015;19(1):403-5.	Excluded by abstract
587	Marklund M, Persson M, Franklin KA. Treatment success with a mandibular advancement device is related to supine-dependent sleep apnea. Chest. 1998;114(6):1630-5.	Excluded by abstract
588	Marklund M, Sahlin C, Stenlund H, Persson M, Franklin KA. Mandibular advancement device in patients with obstructive sleep apnea : long-term effects on apnea and sleep. Chest. 2001;120(1):162-9.	Excluded by abstract
589	Marklund M, Stenlund H, Franklin KA. Mandibular advancement devices in 630 men and women with obstructive sleep apnea and snoring: tolerability and predictors of treatment success. Chest. 2004;125(4):1270-8.	Excluded by abstract
590	Marklund M, Verbraecken J, Randerath W. Non-CPAP therapies in obstructive sleep apnoea: mandibular advancement device therapy. Eur Respir J. 2012;39(5):1241-7.	Excluded by abstract
591	Marklund M. Update on Oral Appliance Therapy for OSA. Current Sleep Medicine Reports. 2017;3(3):143-51.	Excluded by abstract
592	Martínez-Gomis J, Willaert E, Nogues L, Pascual M, Somoza M, Monasterio C. Five years of sleep apnea treatment with a mandibular advancement device side effects and technical complications. Angle Orthodontist. 2010;80(1):30-6.	Excluded by abstract
593	Martínez-González J-M, Martínez-Rodríguez N, Martín-Ares M, Arias-Irimia O, Barona-Dorado C. Odontostomatological therapeutic possibilities in patients with sleep apnea. Med oral patol oral cir bucal (Internet). 2010;15(4):605-10.	Excluded by abstract
594	Martins OFM, Chaves Junior CM, Rossi RRP, Cunali PA, Dal-Fabbro C, Bittencourt L. Side effects of mandibular advancement splints for the treatment of snoring and obstructive sleep apnea: A systematic review. Dental Press Journal of Orthodontics. 2018;23(4).	Excluded by abstract
595	Maurya R, Singh H, Mishra HA, Gupta A. Modified Interim Mandibular Advancement (MIMA) Appliance for Symptomatic Correction of Obstructive Sleep Apnea. J Clin Diagn Res. 2016;10(8):Zh01-3.	Excluded by abstract
596	McGuinness NJ, McDonald JP. Changes in natural head position observed immediately and one year after rapid maxillary expansion. European Journal of Orthodontics. 2006;28(2):126-34.	Excluded by abstract
597	Mehra P, Downie M, Pita MC, Wolford LM. Pharyngeal airway space changes after counterclockwise rotation of the maxillomandibular complex. Am J Orthod Dentofacial Orthop. 2001;120(2):154-9.	Excluded by abstract
598	Meurice JC, Attali V, Collet JM, D'Ortho MP, El Chater P, Kerbrat JB, et al. ORCADES: Efficacy and tolerability of a custom-made CAD/CAM mandibular repositioning device (MRD) for treating obstructive sleep apnoea (OSA). European Respiratory Journal. 2014;44.	Excluded by abstract
599	Meyer A, Rasche K, Hohenhorst W, Ostrowsky A, Pelser M, Randerath W. Mandibular advancement devices for sleep disordered breathing: Consensus paper of North-Rhine Westphalia's society of sleep medicine. Somnologie. 2018;22(4):257-61.	Excluded by abstract
600	Miano S, Rizzoli A, Evangelisti M, Bruni O, Ferri R, Pagani J, et al. NREM sleep instability changes following rapid maxillary expansion in children with obstructive apnea sleep syndrome. Sleep medicine [Internet]. 2009; 10(4):[471-8 pp.]. Available from: https://www.cochranelibrary.com/central/doi/10.1002/central/CN-00701149/full.	Excluded by abstract
601	Mickelson SA. Hyoid advancement to the mandible (hyo-mandibular advancement). Operative Techniques in Otolaryngology - Head and Neck Surgery. 2012;23(1):56-9.	Excluded by abstract
602	Miljus D, Tihacek-Sojic L, Milic-Lemic A, Andjelkovic M. Treatment of obstructive sleep apnea patients using oral appliances--our experiences. Vojnosanit Pregl. 2014;71(7):623-6.	Excluded by abstract
603	Millman RP, Rosenberg CL, Carlisle CC, Kramer NR, Kahn DM, Bonitati AE. The efficacy of oral appliances in the treatment of persistent sleep apnea after uvulopalatopharyngoplasty. Chest. 1998;113(4):992-6.	Excluded by abstract
604	Miloro M. Mandibular distraction osteogenesis for pediatric airway management. J Oral Maxillofac Surg. 2010;68(7):1512-23.	Excluded by abstract
605	Minagi HO, Okuno K, Nohara K, Sakai T. Predictors of side effects with long-term oral appliance therapy for obstructive sleep apnea. Journal of Clinical Sleep Medicine. 2018;14(1):119-25.	Excluded by abstract
606	Moghadam BK, Yousefian JZ. Orofacial complications associated with forward repositioning of the mandible in snore guard users. Gen Dent. 2003;51(6):544-7.	Excluded by abstract
607	Mohsenin N, Mostofi MT, Mohsenin V. The role of oral appliances in treating obstructive sleep apnea. J Am Dent Assoc. 2003;134(4):442-9.	Excluded by abstract
608	Mokhirev MA, Volchek DA, Tardov MV, Ospanova GB, Arutunov GR, Vydrina AE, et al. Orthognatic surgery planning in complex treating obstructive sleep apnea. Part 1. Klinicheskaya stomatologiya. 2019(1):40-5.	Excluded by abstract
609	Moore KE, Esther MS. Current medical management of sleep-related breathing disorders. Oral and Maxillofacial Surgery Clinics of North America. 2002;14(3):297-304.	Excluded by abstract
610	Moscarino S, Kötter F, Brandt M, Modabber A, Kniha K, Hölzle F, et al. Influence of different surgical concepts for moderate skeletal class II and III treatment on the nasopharyngeal airway space. J Craniomaxillofac Surg. 2019;47(10):1489-97.	Excluded by abstract
611	Mostafiz W, Dalci O, Sutherland K, Malhotra A, Srinivasan V, Darendeliler MA, et al. Influence of oral and craniofacial dimensions on mandibular advancement splint treatment outcome in patients with obstructive sleep apnea. Chest. 2011;139(6):1331-9.	Excluded by abstract
612	Motamedi MRK, Motamedi AK. Functional treatment of skeletal Class II malocclusion using bone-anchored devices and intermaxillary elastics. Dental Hypotheses. 2015;6(3):94-6.	Excluded by abstract
613	Nabarro PAD, Hõfling RTB. Efetividade do aparelho ortopédico Bionator de Balters no tratamento do ronco e apnéia do sono. Rev Dent Press Ortod Ortop Facial (Impr). 2008;13(4):36-44.	Excluded by abstract
614	Nakazawa Y, Sakamoto T, Yasutake R, Yamaga K, Kotorii T, Miyahara Y, et al. Treatment of sleep apnea with prosthetic mandibular advancement (PMA). Sleep. 1992;15(6):499-504.	Excluded by abstract
615	Namaki S, Maekawa N, Iwata J, Namaki M, Yonehara Y. Correlation between hyoid bone position, width of pharynx and swallowing function before-after orthognathic surgery for mandibular deficiency. Journal of Oral and Maxillofacial Surgery. 2014;72(9):e124-e5.	Excluded by abstract

616	Nassi Ribak R, Altamirano A, Casasa Araujo A. Correción esquelética clase II, adelantamiento mandibular con aparatología funcional Twin Force. Evaluación a través del haz de cono. Cient dent (Ed impr). 2018;15(1):45-52.	Excluded by abstract
617	Nauert K. Orthodontic treatment of obstructive sleep apnea in children-a case report. Atemwegs- und Lungenkrankheiten. 2019;45(1):46-8.	Excluded by abstract
618	Nazarali N, Altalibi M, Nazarali S, Major MP, Flores-Mir C, Major PW. Mandibular advancement appliances for the treatment of paediatric obstructive sleep apnea: a systematic review. Eur J Orthod. 2015;37(6):618-26.	Excluded by abstract
619	Nazarali N, Altalibi M, Nazarali S, Major MP, Major PM, Flores-Mir C. Mandibular advancement appliances for the treatment of pediatric obstructive sleep apnea: A systematic review. Sleep. 2015;38:A363.	Excluded by abstract
620	Ng A, Gotsopoulos H, Darendeliler AM, Cistulli PA. Oral appliance therapy for obstructive sleep apnea. Treat Respir Med. 2005;4(6):409-22.	Excluded by abstract
621	Ng AT, Darendeliler MA, Petocz P, Cistulli PA. Cephalometry and prediction of oral appliance treatment outcome. Sleep Breath. 2012;16(1):47-58.	Excluded by abstract
622	Ng AT, Qian J, Cistulli PA. Oropharyngeal collapse predicts treatment response with oral appliance therapy in obstructive sleep apnea. Sleep. 2006;29(5):666-71.	Excluded by abstract
623	Ng JH, Yow M. Oral Appliances in the Management of Obstructive Sleep Apnea. Sleep Med Clin. 2019;14(1):109-18.	Excluded by abstract
624	Ngan PW, Wei SH, Yen PK. Orthodontic treatment of the primary dentition. J Am Dent Assoc. 1988;116(3):336-40.	Excluded by abstract
625	Ngiam J, Balasubramaniam R, Darendeliler MA, Cheng AT, Waters K, Sullivan CE. Clinical guidelines for oral appliance therapy in the treatment of snoring and obstructive sleep apnoea. Australian Dental Journal. 2013;58(4):408-19.	Excluded by abstract
626	Ngiam J, Kyung HM. Microimplant mandibular advancement (MiMA) therapy for the treatment of snoring and obstructive sleep apnea (OSA). Korean Journal of Orthodontics. 2010;40(2):115-26.	Excluded by abstract
627	Ngiam J, Kyung HM. Microimplant-based mandibular advancement therapy for the treatment of snoring and obstructive sleep apnea: a prospective study. Angle Orthod. 2012;82(6):978-84.	Excluded by abstract
628	Ngo R, Pullano E, Peacock ZS, Lahey ET, August M. Does the Medical Comorbidity Profile of Obstructive Sleep Apnea Patients Treated With Maxillomandibular Advancement Differ From That of Obstructive Sleep Apnea Patients Managed Nonsurgically? Journal of Oral and Maxillofacial Surgery. 2018;76(9):1999.e1-.e8.	Excluded by abstract
629	Nie P, Zhu M, Lu XF, Fang B. Bone-anchored maxillary expansion and bilateral interoral mandibular distraction osteogenesis in adult with severe obstructive sleep apnea syndrome. J Craniofac Surg. 2013;24(3):949-52.	Excluded by abstract
630	Nishigawa K, Hayama R, Matsuka Y. Complications causing patients to discontinue using oral appliances for treatment of obstructive sleep apnea. J Prosthodont Res. 2017;61(2):133-8.	Excluded by abstract
631	Norrhem N, Marklund M. An oral appliance with or without elastic bands to control mouth opening during sleep-a randomized pilot study. Sleep Breath. 2016;20(3):929-38.	Excluded by abstract
632	Norrhem N, Nemeczek H, Marklund M. Changes in lower incisor irregularity during treatment with oral sleep apnea appliances. Sleep Breath. 2017;21(3):607-13.	Excluded by abstract
633	Nout E, Koudstaal MJ, Wolvius EB, Van der Wal KG. Additional orthognathic surgery following Le Fort III and monobloc advancement. Int J Oral Maxillofac Surg. 2011;40(7):679-84.	Excluded by abstract
634	Nunes Junior WR, Oliveira I, Faria MEJ, Di Francesco RC. Prader-willy syndrome and the multiprofessional treatment of sleep apnea: A case report. Sleep Medicine. 2009;10:S73.	Excluded by abstract
635	Okawara Y, Tsuiki S, Hiyama S, Hashimoto K, Ono T, Ohyama K. Oral appliance titration and nasal resistance in nonapneic subjects. Am J Orthod Dentofacial Orthop. 2004;126(5):620-2.	Excluded by abstract
636	Okuno K, Ikai K, Matsumura-Ai E, Araie T. Titration technique using endoscopy for an oral appliance treatment of obstructive sleep apnea. J Prosthet Dent. 2018;119(3):350-3.	Excluded by abstract
637	Okushi T, Tonogi M, Arisaka T, Kobayashi S, Tsukamoto Y, Morishita H, et al. Effect of maxillomandibular advancement on morphology of velopharyngeal space. J Oral Maxillofac Surg. 2011;69(3):877-84.	Excluded by abstract
638	Ono T, Lowe A, Ferguson K, Pae E, Fleetham J. The effect of the tongue retaining device on awake genioglossus muscle activity in patients with obstructive sleep apnea. American journal of orthodontics and dentofacial orthopedics [Internet]. 1996; 110(1):[28-35 pp.]. Available from: https://www.cochranelibrary.com/central/doi/10.1002/central/CN-00126945/full.	Excluded by abstract
639	Ortu E, Aprile G, Cattaneo R, Pietropaoli D, Mummolo A, Monaco A. Use of EQ O.S.A. device in management of malocclusion: A clinical case. Dental Cadmos. 2020;88(1):47-52.	Excluded by abstract
640	O'Sullivan RA, Hillman DR, Mateljan R, Pantin C, Finucane KE. Mandibular advancement splint: an appliance to treat snoring and obstructive sleep apnea. Am J Respir Crit Care Med. 1995;151(1):194-8.	Excluded by abstract
641	Otsuka R, Ribeiro de Almeida F, Lowe AA, Linden W, Ryan F. The effect of oral appliance therapy on blood pressure in patients with obstructive sleep apnea. Sleep Breath. 2006;10(1):29-36.	Excluded by abstract
642	Ottaviano G, Maculan P, Borghetto G, Favero V, Galletti B, Savietto E, et al. Nasal function before and after rapid maxillary expansion in children: a randomized, prospective, controlled study. International journal of pediatric otorhinolaryngology [Internet]. 2018; 115:[133-8 pp.]. Available from: https://www.cochranelibrary.com/central/doi/10.1002/central/CN-01652855/full.	Excluded by abstract
643	Padma A, Ramakrishnan N, Narayanan V. Management of obstructive sleep apnea: A dental perspective. Indian J Dent Res. 2007;18(4):201-9.	Excluded by abstract
644	Paliga JT, Goldstein JA, Storm PB, Taylor JA. Monobloc minus Le Fort II for single-stage treatment of the Apert phenotype. J Craniofac Surg. 2013;24(4):1380-2.	Excluded by abstract
645	Palm E, Franklin KA, Marklund M. Mandibular tori size is related to obstructive sleep apnea and treatment success with an oral appliance. Sleep Breath. 2014;18(2):431-8.	Excluded by abstract
646	Palotie T, Riekki S, Makitie A, Bachour A, Arte S, Back L. The Effect of mandible advancement splints in mild, moderate, and severe obstructive sleep apnea-the need for sleep registrations during follow up. Eur J Orthod. 2017;39(5):497-501.	Excluded by abstract
647	Paluch Z, Twardokęs M, Stelmańska K, Cielińska M. Influence of orthodontic treatment with functional appliances on pharyngeal dimensions in patients with Class II malocclusions-Review of literature. Journal of Stomatology. 2015;68(5):559-65.	Excluded by abstract
648	Panula K, Keski-Nisula K. Irreversible alteration in occlusion caused by a mandibular advancement appliance: an unexpected complication of sleep apnea treatment. Int J Adult Orthodon Orthognath Surg. 2000;15(3):192-6.	Excluded by abstract
649	Paredes-Gallardo V, Garcia-Sanz V, Bellot-Arcis C. Miniscrew-assisted multidisciplinary orthodontic treatment with surgical mandibular advancement and genioplasty in a brachyfacial Class II patient with mandibular asymmetry. American Journal of Orthodontics and Dentofacial Orthopedics. 2017;152(5):679-92.	Excluded by abstract
650	Parsi GK, Alsulaiman AA, Kotak B, Mehra P, Will LA, Motro M. Volumetric changes of the upper airway following maxillary and mandibular advancement using cone beam computed tomography. Int J Oral Maxillofac Surg. 2019;48(2):203-10.	Excluded by abstract
651	Patel D, Ash S, Evans J. The role of orthodontics and oral and maxillofacial surgery in the management of obstructive sleep apnoea - a single case report. Br Dent J. 2004;196(5):264-7.	Excluded by abstract
652	Paul SA, Simon SS, Issac B, Kumar S. Management of severe sleep apnea secondary to juvenile arthritis with temporomandibular joint replacement and mandibular advancement. Journal of Pharmacy and Bioallied Sciences. 2015;7(6):S687-S90.	Excluded by abstract
653	Perez CV, de Leeuw R, Okeson JP, Carlson CR, Li HF, Bush HM, et al. The incidence and prevalence of temporomandibular disorders and posterior open bite in patients receiving mandibular advancement device therapy for obstructive sleep apnea. Sleep Breath. 2013;17(1):323-32.	Excluded by abstract
654	Phillips CL, Grunstein RR, Darendeliler A, Mihailidou A, Yee BJ, Cistulli PA. A comparative effectiveness trial of continuous positive airway pressure (CPAP) versus oral appliance (OA) therapy in obstructive sleep apnea (OSA). Sleep and Biological Rhythms. 2011;9(4):294.	Excluded by abstract
655	Pliska B, Lowe AA, Almeida FR. The orthodontist and the obstructive sleep apnea patient. Int J Orthod Milwaukee. 2012;23(3):19-22.	Excluded by abstract
656	Pliska BT, Almeida F. Effectiveness and outcome of oral appliance therapy. Dent Clin North Am. 2012;56(2):433-44.	Excluded by abstract
657	Pliska BT, Nam H, Chen H, Lowe AA, Almeida FR. Oral appliance treatment of obstructive sleep apnea: Progression of long-term side effects. Sleep and Breathing. 2012;16(3):925-6.	Excluded by abstract

658	Prabhakar RR, Saravanan R, Karthikeyan MK, Vishnuchandran C, Sudeepthi. Prevalence of malocclusion and need for early orthodontic treatment in children. J Clin Diagn Res. 2014;8(5):Zc60-1.	Excluded by abstract
659	Prabhat KC, Goyal L, Bey A, Maheshwari S. Recent advances in the management of obstructive sleep apnea: The dental perspective. J Nat Sci Biol Med. 2012;3(2):113-7.	Excluded by abstract
660	Prado FB, Rossi AC, Freire AR, Groppo FC, De Moraes M, Caria PH. Pharyngeal airway space and frontal and sphenoid sinus changes after maxillomandibular advancement with counterclockwise rotation for Class II anterior open bite malocclusions. Dentomaxillofac Radiol. 2012;41(2):103-9.	Excluded by abstract
661	Prathibha BN, Jagger RG, Saunders M, Smith AP. Use of a mandibular advancement device in obstructive sleep apnoea. J Oral Rehabil. 2003;30(5):507-9.	Excluded by abstract
662	Prescinotto R, Haddad FL, Fukuchi I, Gregorio LC, Cunali PA, Tufik S, et al. Impact of upper airway abnormalities on the success and adherence to mandibular advancement device treatment in patients with Obstructive Sleep Apnea Syndrome. Braz J Otorhinolaryngol. 2015;81(6):663-70.	Excluded by abstract
663	Qaseem A, Holty JE, Owens DK, Dallas P, Starkey M, Shekelle P. Management of obstructive sleep apnea in adults: A clinical practice guideline from the American College of Physicians. Ann Intern Med. 2013;159(7):471-83.	Excluded by abstract
664	Quinnell TG, Clutterbuck-James AL. Alternatives to continuous positive airway pressure 2: mandibular advancement devices compared. Curr Opin Pulm Med. 2014;20(6):595-600.	Excluded by abstract
665	Quintela MdM, Uechi CH, Pacheco Filho F, Motta RHL, Flório FM. Avaliação da concordância inicial do uso de aparelhos utilizados na terapia dos distúrbios respiratórios do sono. Ortho Sci, Orthod sci pract. 2010;3(11):198-204.	Excluded by abstract
666	Quintela MdM, Vedovello Filho M, Yoshida AH, Flório FM, Motta RHL. Aparelhos de avanço mandibular para apneia obstrutiva do sono: evoluções técnicas e protocolos clínicos. Ortodontia. 2009;42(1):50-8.	Excluded by abstract
667	Raffaini M, Pisani C. Clinical and cone-beam computed tomography evaluation of the three-dimensional increase in pharyngeal airway space following maxillo-mandibular rotation-advancement for Class II-correction in patients without sleep apnoea (OSA). J Craniomaxillofac Surg. 2013;41(7):552-7.	Excluded by abstract
668	Ramar K, Dort LC, Katz SG, Lettieri CJ, Harrod CG, Thomas SM, et al. Clinical Practice Guideline for the Treatment of Obstructive Sleep Apnea and Snoring with Oral Appliance Therapy: An Update for 2015. J Clin Sleep Med. 2015;11(7):773-827.	Excluded by abstract
669	Randerath WJ, Heise M, Hinz R, Ruehle KH. An individually adjustable oral appliance vs continuous positive airway pressure in mild-to-moderate obstructive sleep apnea syndrome. Chest. 2002;122(2):569-75.	Excluded by abstract
670	Randerath WJ. Alternatives to positive airway pressure for obstructive sleep apnea syndrome. Expert Review of Respiratory Medicine. 2009;3(3):255-63.	Excluded by abstract
671	Ranieri AL, Tufik S, de Siqueira JT. Refractory cluster headache in a patient with bruxism and obstructive sleep apnea: a case report. Sleep Breath. 2009;13(4):429-33.	Excluded by abstract
672	Ranieri S, Laganà G, Lombardo EC, Cozza P. Sleep breathing disorders in adult: Role of orthodontist. Dental Cadmos. 2018;86(6):501-14.	Excluded by abstract
673	Ravizzini A. El equilibrio biológico: casos clínicos. Rev Asoc Argent Ortop Funcional Maxilares. 2006;35(2):21-9.	Excluded by abstract
674	Remmelink HJ, Hoeke A. [Dutch national guidelines for diagnosis and treatment of obstructive sleep apnea syndrome in adults]. Ned Tijdschr Tandheelkd. 2010;117(4):227-31.	Excluded by abstract
675	Robertson CJ. Obstructive sleep apnoea. Part II: Treatment with a customised dental appliance. N Z Dent J. 1997;93(411):4-9.	Excluded by abstract
676	Robertson CJ. Treatment of long-standing nocturnal enuresis by mandibular advancement. Sleep Breath. 2004;8(1):57-60.	Excluded by abstract
677	Rodriguez-Lozano FJ, Saez-Yuguero Mdel R, Linares Tovar E, Bermejo Fenoll A. Sleep apnea and mandibular advancement device. Revision of the literature. Med Oral Patol Oral Cir Bucal. 2008;13(9):E549-54.	Excluded by abstract
678	Rohida NS, Bhad WA. Accidental ingestion of a fractured Twin-block appliance. Am J Orthod Dentofacial Orthop. 2011;139(1):123-5.	Excluded by abstract
679	Ronchi P, Cinquini V, Ambrosoli A, Caprioglio A. Maxillomandibular advancement in obstructive sleep apnea syndrome patients: a restrospective study on the sagittal cephalometric variables. J Oral Maxillofac Res. 2013;4(2):e5.	Excluded by abstract
680	Ronchi P, Novelli G, Colombo L, Valsecchi S, Oldani A, Zucconi M, et al. Effectiveness of maxillo-mandibular advancement in obstructive sleep apnea patients with and without skeletal anomalies. Int J Oral Maxillofac Surg. 2010;39(6):541-7.	Excluded by abstract
681	Rondeau B. The benefits of early orthodontic treatment. Gen Dent. 2003;51(2):114-9.	Excluded by abstract
682	Rosario HD, Oliveira GMS, Freires IA, de Souza Matos F, Paranhos LR. Efficiency of bimaxillary advancement surgery in increasing the volume of the upper airways: a systematic review of observational studies and meta-analysis. Eur Arch Otorhinolaryngol. 2017;274(1):35-44.	Excluded by abstract
683	Rose E, Ridder GJ, Staats R. [Endoscopically-assisted adjustment of an oral appliance in patients with obstructive sleep apnoea]. Laryngorhinootologie. 2002;81(9):619-23.	Excluded by abstract
684	Rose E, Staats R, Schulte-Monting J, Jonas IE. Treatment of obstructive sleep apnea with the Karwetzky oral appliance. Eur J Oral Sci. 2002;110(2):99-105.	Excluded by abstract
685	Rose E. [Use of oral appliances in therapy of sleep related breathing disorders]. Laryngorhinootologie. 2006;85 Suppl 1:26-9.	Excluded by abstract
686	Rose EC, Barthlen GM, Staats R, Jonas IE. Therapeutic efficacy of an oral appliance in the treatment of obstructive sleep apnea: a 2-year follow-up. Am J Orthod Dentofacial Orthop. 2002;121(3):273-9.	Excluded by abstract
687	Rose EC, Germann M, Sorichter S, Jonas IE. Case control study in the treatment of obstructive sleep-disordered breathing with an intraoral protrusive appliance. J Orofac Orthop. 2004;65(6):489-500.	Excluded by abstract
688	Rose EC, Staats R, Virchow C, Jr., Jonas IE. Occlusal and skeletal effects of an oral appliance in the treatment of obstructive sleep apnea. Chest. 2002;122(3):871-7.	Excluded by abstract
689	Rossi M, Wolford LM, Cevidanes L, Cassano D, Cunha L, Mansur D, et al. TMJ condylar changes after bimaxillary advancement and disc repositioning. Journal of Oral and Maxillofacial Surgery. 2012;70(9):e-94.	Excluded by abstract
690	Rubio-Bueno P, Capote Moreno A, Landete P, Zamora E, Wix R, Ancochea J, et al. Apnea obstructiva del sueño: un abordaje innovador mínimamente invasivo mediante distracción de rama mandibular. Rev esp cir oral maxilofac. 2018;40(2):55-64.	Excluded by abstract
691	Rubio-Bueno P, Capote Moreno A, Landete P, Zamora E, Wix R, Ancochea J, et al. Obstructive sleep apnoea: An innovative minimally invasive approach using mandibular branch distraction. Revista Espanola de Cirugia Oral y Maxilofacial. 2017.	Excluded by abstract
692	Ruoff CM, Guilleminault C. Orthodontics and sleep-disordered breathing. Sleep Breath. 2012;16(2):271-3.	Excluded by abstract
693	Saglam-Aydinatay B, Taner T. Oral appliance therapy in obstructive sleep apnea: Long-term adherence and patients experiences. Med oral patol oral cir bucal (Internet). 2018;23(1):e72-e7.	Excluded by abstract
694	Sahoo NK, Jayan B, Ramakrishna N, Chopra SS, Kochar G. Evaluation of upper airway dimensional changes and hyoid position following mandibular advancement in patients with skeletal class II malocclusion. J Craniofac Surg. 2012;23(6):e623-7.	Excluded by abstract
695	Sakamoto Y, Yanamoto S, Rokutanda S, Naruse T, Imayama N, Hashimoto M, et al. Predictors of obstructive sleep apnoea-hypopnea severity and oral appliance therapy efficacy by using lateral cephalometric analysis. J Oral Rehabil. 2016;43(9):649-55.	Excluded by abstract
696	Sampol Rubio G, Macías Escalada E, Montserrat Canal JM, Terán Santos J. Los dispositivos de avance mandibular en el tratamiento de la apnea obstructiva del sueño. Una opción necesaria y eficaz. Med clín (Ed impr). 2018;151(1):34-8.	Excluded by abstract
697	Sánchez-Ariza CA. Tratamiento con dispositivos orales para síndrome de apnea-hipopnea obstructiva del sueño (SAHOS). Rev Fac Med (Bogotá). 2017;65(supl.1):121-8.	Excluded by abstract
698	Sander FG. [New oral plate]. Zahnarztl Mitt. 1989;79(16):1769-70, 72, 75.	Excluded by abstract
699	Sander FG. Mouth opening and its influencing through the SII appliance during the night. J Orofac Orthop. 2001;62(2):133-45.	Excluded by abstract
700	Santos Junior JFd, Abrahão M, Gregório LC, Zonato AI, Gumieiro EH. Mentoplastia para avanço do músculo genioglosso em pacientes com síndrome da apnéia-hipopnéia do sono obstrutiva e retrognatismo mandibular. Rev bras otorrinolaringol. 2007;73(4):480-6.	Excluded by abstract
701	Sasayama S, Izumi T, Matsuzaki M, Matsumori A, Asanoi H, Momomura S, et al. Improvement of quality of life with nocturnal oxygen therapy in heart failure patients with central sleep apnea. Circulation journal [Internet]. 2009; 73(7):[1255-62 pp.]. Available from: https://www.cochranelibrary.com/central/doi/10.1002/central/CN-00719452/full.	Excluded by abstract

702	Sasayama S, Izumi T, Seino Y, Ueshima K, Asanoi H. Effects of nocturnal oxygen therapy on outcome measures in patients with chronic heart failure and cheyne-stokes respiration. Circulation journal [Internet]. 2006; 70(1):[1-7 pp.]. Available from: https://www.cochranelibrary.com/central/doi/10.1002/central/CN-00553855/full.	Excluded by abstract
703	Sato K. [Oral appliances used in treating obstructive sleep apnea syndrome--participation by otolaryngologists]. Nihon Jibiinkoka Gakkai Kaiho. 2003;106(2):150-5.	Excluded by abstract
704	Schendel S, Powell N, Jacobson R. Maxillary, mandibular, and chin advancement: treatment planning based on airway anatomy in obstructive sleep apnea. J Oral Maxillofac Surg. 2011;69(3):663-76.	Excluded by abstract
705	Schendel SA, Broujerdi JA, Jacobson RL. Three-dimensional upper-airway changes with maxillomandibular advancement for obstructive sleep apnea treatment. Am J Orthod Dentofacial Orthop. 2014;146(3):385-93.	Excluded by abstract
706	Schessl J, Rose E, Korinthenberg R, Henschen M. Severe obstructive sleep apnea alleviated by oral appliance in a three-year-old boy. Respiration. 2008;76(1):112-6.	Excluded by abstract
707	Schierle HP, Schliephake H, Dempf R, Freihorst J. [Experiences with distraction osteogenesis in therapy of severe peripheral airway obstruction in infancy and early childhood]. Mund Kiefer Gesichtschir. 1998;2(3):146-52.	Excluded by abstract
708	Schwarting S, Huebers U, Heise M, Schlieper J, Hauschild A. Position paper on the use of mandibular advancement devices in adults with sleep-related breathing disorders. A position paper of the German Society of Dental Sleep Medicine (Deutsche Gesellschaft Zahnaerztliche Schlafmedizin, DGZS). Sleep Breath. 2007;11(2):125-6.	Excluded by abstract
709	Seehra J, Sherriff M, Winchester L. Craniofacial characteristics of successful responders to mandibular advancement splint therapy: a pilot study. British Journal of Oral & Maxillofacial Surgery. 2014;52(4):314-6.	Excluded by abstract
710	Sharma S, Essick G, Schwartz D, Aronsky AJ. Sleep medicine care under one roof: a proposed model for integrating dentistry and medicine. J Clin Sleep Med. 2013;9(8):827-33.	Excluded by abstract
711	Shen HL, Wen YW, Chen NH, Liao YF. Craniofacial morphologic predictors of oral appliance outcomes in patients with obstructive sleep apnea. J Am Dent Assoc. 2012;143(11):1209-17.	Excluded by abstract
712	Shott SR. Evaluation and management of pediatric obstructive sleep apnea beyond tonsillectomy and adenoidectomy. Curr Opin Otolaryngol Head Neck Surg. 2011;19(6):449-54.	Excluded by abstract
713	Shrivastava D, Bixby JK, Livornese DS, Urena F, Bixby MJ, Jain V. Efficacy of Oral Appliance Therapy in the Treatment of Severe OSA in CPAP-Resistant Cases. Sleep and Vigilance. 2018;2(2):119-25.	Excluded by abstract
714	Silveira M. A síndrome da apnéia obstrutiva do sono, o ronco e seu tratamento com o aparelho Apnout. J bras ortodon ortop facial. 2001;6(32):151-4.	Excluded by abstract
715	Singh GD, Callister JD. Effect of a maxillary appliance in an adult with obstructive sleep apnea: a case report. Cranio. 2013;31(3):171-5.	Excluded by abstract
716	Sjoholm TT, Polo OJ, Rauhala ER, Vuoriluoto J, Helenius HY. Mandibular advancement with dental appliances in obstructive sleep apnoea. J Oral Rehabil. 1994;21(5):595-603.	Excluded by abstract
717	Smith AM, Battagel JM. Non-apneic snoring and the orthodontist: radiographic pharyngeal dimension changes with supine posture and mandibular protrusion. J Orthod. 2004;31(2):124-31.	Excluded by abstract
718	Smith AM, Battagel JM. Non-apneic snoring and the orthodontist: the effectiveness of mandibular advancement splints. J Orthod. 2004;31(2):115-23.	Excluded by abstract
719	Smith DM, Stradling JR. Can mandibular advancement devices be a satisfactory substitute for short term use in patients on nasal continuous positive airway pressure? Thorax. 2002;57(4):305-8.	Excluded by abstract
720	Smith I. The choice of treatment for obstructive sleep apnoea. CME Bulletin Respiratory Medicine. 2000;2(3):64-8.	Excluded by abstract
721	Smith RK, Cruz S. Monitoring mandibular advancement appliances: Ten most common problems. Sleep and Breathing. 2012;16(3):921-2.	Excluded by abstract
722	Smith SD. Oral appliances in the treatment of obstructive sleep apnea. Atlas Oral Maxillofac Surg Clin North Am. 2007;15(2):193-211.	Excluded by abstract
723	Snatos Junior JFd, Aidar LAdA, Dominguez-Rodriguez GC, Novikoff S, Abrahão M. Avaliação do espaço aéreo faríngeo em pacientes com retrognatismo mandibular tratados com aparelho Herbst. Ortodontia. 2004;37(3):8-13.	Excluded by abstract
724	Stellzig-Eisenhauer A, Meyer-Marcotty P. [Interaction between otorhinolaryngology and orthodontics: correlation between the nasopharyngeal airway and the craniofacial complex]. Laryngorhinootologie. 2010;89 Suppl 1:S72-8.	Excluded by abstract
725	Suga H, Mishima K, Nakano H, Nakano A, Matsumura M, Mano T, et al. Different therapeutic mechanisms of rigid and semi-rigid mandibular repositioning devices in obstructive sleep apnea syndrome. J Craniomaxillofac Surg. 2014;42(8):1650-4.	Excluded by abstract
726	Sunitha C, Kumar SA. Obstructive sleep apnea and its management. Indian J Dent Res. 2010;21(1):119-24.	Excluded by abstract
727	Susarla SM, Abramson ZR, Dodson TB, Kaban LB. Upper airway length decreases after maxillomandibular advancement in patients with obstructive sleep apnea. J Oral Maxillofac Surg. 2011;69(11):2872-8.	Excluded by abstract
728	Sutherland K, Blignaut A, Dalci O, Chan A, Darendeliler A, Cistulli P. Craniofacial assessment of oral appliance treatment responders and non-responders using cone beam computed tomography (CBCT). Sleep and Biological Rhythms. 2014;12:30.	Excluded by abstract
729	Sutherland K, Chan A, Singh P, Ngiam J, Dalci O, Darendeliler A, et al. Multimodal phenotyping for prediction of oral appliance treatment outcome in obstructive sleep apnoea. Sleep and Biological Rhythms. 2014;12:46.	Excluded by abstract
730	Sutherland K, Chan ASL, Ngiam J, Dalci O, Darendeliler MA, Cistulli PA. Awake Multimodal Phenotyping for Prediction of Oral Appliance Treatment Outcome. J Clin Sleep Med. 2018;14(11):1879-87.	Excluded by abstract
731	Sutherland K, Deane SA, Chan AS, Schwab R, Zeng B, Ng AT, et al. Comparative effects of mandibular advancement splint and tongue stabilising device on upper airway structure in obstructive sleep apnea. Sleep. 2010;33:A122-A3.	Excluded by abstract
732	Sutherland K, Mostafiz W, Dalci O, Malhotra A, Srinivasan V, Darendeliler MA, et al. Influence of oral dimensions on mandibular advancement splint treatment outcome in obstructive sleep apnoea. Sleep and Biological Rhythms. 2010;8:A49.	Excluded by abstract
733	Sutherland K, Vanderveken OM, Tsuda H, Marklund M, Gagnadoux F, Kushida CA, et al. Oral appliance treatment for obstructive sleep apnea: an update. J Clin Sleep Med. 2014;10(2):215-27.	Excluded by abstract
734	Sutherland K, Williams M, Lee R, Srinivasan V, Chan A, Darendeliler MA, et al. Photographic craniofacial analysis and mandibular advancement splint treatment outcome. Sleep and Biological Rhythms. 2010;8:A45.	Excluded by abstract
735	Suzuki H, Ebato A, Yasuda A, Takeuchi H, Chow CM, Komiyama O. Concurrent treatment of obstructive sleep apnea with a two-piece mandibular advancement device with an elastic retention band and orofacial myofunctional therapy: A case report. Journal of Sleep Research. 2018;27.	Excluded by abstract
736	Svanholt P, Petri N, Wildschiodtz G, Sonnesen L, Kjaer I. Associations between craniofacial morphology, head posture, and cervical vertebral body fusions in men with sleep apnea. American Journal of Orthodontics and Dentofacial Orthopedics. 2009;135(6).	Excluded by abstract
737	Taddei M, Alkhamis N, Tagariello T, D'Alessandro G, Mariucci EM, Piana G. Effects of rapid maxillary expansion and mandibular advancement on upper airways in Marfan's syndrome children: a home sleep study and cephalometric evaluation. Sleep Breath. 2015;19(4):1213-20.	Excluded by abstract
738	Tan HL, Kheirandish-Gozal L, Gozal D. Adenotonsillectomy in Pediatric OSA: Time to Look Elsewhere. Current Sleep Medicine Reports. 2018;4(3):243-53.	Excluded by abstract
739	Tan SK, Leung WK, Tang ATH, Zwahlen RA. Effects of mandibular setback with or without maxillary advancement osteotomies on pharyngeal airways: An overview of systematic reviews. PLoS One. 2017;12(10):e0185951.	Excluded by abstract
740	Tan SK, Leung WK, Tang ATH, Zwahlen RA. How does mandibular advancement with or without maxillary procedures affect pharyngeal airways? An overview of systematic reviews. PLoS One. 2017;12(7):e0181146.	Excluded by abstract
741	Tan SK, Leung WK, Tang ATH, Zwahlen RA. Letter to the editor on the article "Impact on the upper airway space of different types of orthognathic surgery for the correction of skeletal class III malocclusion: A systematic review and meta-analysis". Int J Surg. 2017;45:156-7.	Excluded by abstract
742	Tan YK, L'Estrange PR, Luo YM, Smith C, Grant HR, Simonds AK, et al. Mandibular advancement splints and continuous positive airway pressure in patients with obstructive sleep apnoea: a randomized cross-over trial. Eur J Orthod. 2002;24(3):239-49.	Excluded by abstract
743	Tanoue N, Nagano K, Yanamoto S, Mizuno A. Comparative evaluation of the breaking strength of a simple mobile mandibular advancement splint. Eur J Orthod. 2009;31(6):620-4.	Excluded by abstract

744	Tartaglia GM, Grandi G, Mian F, Sforza C, Ferrario VF. Non-invasive 3D facial analysis and surface electromyography during functional pre-orthodontic therapy: a preliminary report. J appl oral sci. 2009;17(5):487-94.	Excluded by abstract
745	Teixeira AOB, Andrade ALL, Almeida R, Almeida MAO. Side effects of intraoral devices for OSAS treatment. Braz J Otorhinolaryngol. 2018;84(6):772-80.	Excluded by abstract
746	Ten Berge DM, Braem MJ, Altenburg A, Dieltjens M, Van de Heyning PH, Vanhaecht K, et al. Evaluation of the impact of a clinical pathway on the organization of a multidisciplinary dental sleep clinic. Sleep Breath. 2014;18(2):325-34.	Excluded by abstract
747	Thomas D, Markman S, Sofferman B. Sleep basics and sleep-pain interrelations for orofacial pain dentists. The Alpha omegan. 2013;106(1-2):29-33.	Excluded by abstract
748	Tison C, Sebille-Elhage S, Ferri J. [Mandibular advancement device: a 5-year long experience in obstructive sleep apnea/hypopnea syndrome]. Rev Stomatol Chir Maxillofac. 2011;112(2):80-6.	Excluded by abstract
749	Torres HM, Valladares-Neto J, Torres EM, Freitas RZ, Silva MA. Effect of Genioplasty on the Pharyngeal Airway Space Following Maxillomandibular Advancement Surgery. J Oral Maxillofac Surg. 2017;75(1):189.e1-.e12.	Excluded by abstract
750	Trento GdS, Santos FAOdS, Klüppel LE, Costa DJd, Rebellato NLB, Scariot R. Pharyngeal airspace in patients undergoing orthognathic surgery for mandibular advancement. Braz j oral sci. 2015;14(2):112-6.	Excluded by abstract
751	Tsuiki S, Hiyama S, Ono T, Imamura N, Ishiwata Y, Kuroda T, et al. Effects of a titratable oral appliance on supine airway size in awake non-apneic individuals. Sleep. 2001;24(5):554-60.	Excluded by abstract
752	Tsuiki S, Ito E, Isono S, Ryan CF, Komada Y, Matsuura M, et al. Oropharyngeal crowding and obesity as predictors of oral appliance treatment response to moderate obstructive sleep apnea. Chest. 2013;144(2):558-63.	Excluded by abstract
753	Ubaldo ED, Greenlee GM, Moore J, Sommers E, Bollen AM. Cephalometric analysis and long-term outcomes of orthognathic surgical treatment for obstructive sleep apnoea. Int J Oral Maxillofac Surg. 2015;44(6):752-9.	Excluded by abstract
754	Ueda H, Almeida FR, Chen H, Lowe AA. Effect of 2 jaw exercises on occlusal function in patients with obstructive sleep apnea during oral appliance therapy: A randomized controlled trial. American Journal of Orthodontics and Dentofacial Orthopedics. 2009;135(4).	Excluded by abstract
755	Ueda H, Almeida FR, Lowe AA, Ruse ND. Changes in occlusal contact area during oral appliance therapy assessed on study models. Angle Orthodontist. 2008;78(5):866-72.	Excluded by abstract
756	Vanderveken OM, Devolder A, Marklund M, Boudewyns AN, Braem MJ, Okkerse W, et al. Comparison of a custom-made and a thermoplastic oral appliance for the treatment of mild sleep apnea. Am J Respir Crit Care Med. 2008;178(2):197-202.	Excluded by abstract
757	Vanderveken OM, Van de Heyning P, Braem MJ. Retention of mandibular advancement devices in the treatment of obstructive sleep apnea: an in vitro pilot study. Sleep Breath. 2014;18(2):313-8.	Excluded by abstract
758	Veldhuis SK, Doff MH, Stegenga B, Nieuwenhuis JA, Wijkstra PJ. Oral appliance to assist non-invasive ventilation in a patient with amyotrophic lateral sclerosis. Sleep Breath. 2015;19(1):61-3.	Excluded by abstract
759	Verbruggen AE, Dieltjens M, Wouters K, De Volder I, Van de Heyning PH, Braem MJ, et al. Prevalence of residual excessive sleepiness during effective oral appliance therapy for sleep-disordered breathing. Sleep Med. 2014;15(2):269-72.	Excluded by abstract
760	Verburg FE, Bollen KHA, Donker HJ, Kramer GJC. The effectiveness of two types of MADS for OSA therapy. Clin Oral Investig. 2018;22(5):1995-2003.	Excluded by abstract
761	Vieira BB, Itikawa CE, de Almeida LA, Sander HH, Aragon DC, Anselmo-Lima WT, et al. Facial features and hyoid bone position in preschool children with obstructive sleep apnea syndrome. Eur Arch Otorhinolaryngol. 2014;271(5):1305-9.	Excluded by abstract
762	Vieira BB, Itikawa CE, de Almeida LA, Sander HS, Fernandes RM, Anselmo-Lima WT, et al. Cephalometric evaluation of facial pattern and hyoid bone position in children with obstructive sleep apnea syndrome. Int J Pediatr Otorhinolaryngol. 2011;75(3):383-6.	Excluded by abstract
763	Villa MP, Miano S, Rizzoli A. Mandibular advancement devices are an alternative and valid treatment for pediatric obstructive sleep apnea syndrome. Sleep Breath. 2012;16(4):971-6.	Excluded by abstract
764	Wang J, Zheng W, Zhang Z, Li C, Zhou Y. [Percutaneous anterior odontoid and transarticular screw fixation for type II odontoid fractures in elderly patients]. Zhongguo Xiu Fu Chong Jian Wai Ke Za Zhi. 2013;27(9):1090-3.	Excluded by abstract
765	Wang L, Liu YH. [Comparison of the efficacy of 2 types of mandibular advancement device in severe obstructive sleep apnea hypopnea syndrome]. Shanghai Kou Qiang Yi Xue. 2014;23(6):713-7.	Excluded by abstract
766	Wardly D, Wolford LM, Veerappan V. Idiopathic intracranial hypertension eliminated by counterclockwise maxillomandibular advancement: a case report. Cranio. 2017;35(4):259-67.	Excluded by abstract
767	Weaver TE, Calik MW, Farabi SS, Fink AM, Galang-Boquiren MT, Kapella MC, et al. Innovative treatments for adults with obstructive sleep apnea. Nat Sci Sleep. 2014;6:137-47.	Excluded by abstract
768	Wilhelmsson B, Tegelberg A, Walker-Engstrom ML, Ringqvist M, Andersson L, Krekmanov L, et al. A prospective randomized study of a dental appliance compared with uvulopalatopharyngoplasty in the treatment of obstructive sleep apnoea. Acta Otolaryngol. 1999;119(4):503-9.	Excluded by abstract
769	Wishney M, Darendeliler MA, Dalci O. Myofunctional therapy and prefabricated functional appliances: an overview of the history and evidence. Australian Dental Journal. 2019;64(2):135-44.	Excluded by abstract
770	Yadav R, Bhutia O, Shukla G, Roychoudhury A. Distraction osteogenesis for management of obstructive sleep apnoea in temporomandibular joint ankylosis patients before the release of joint. Journal of Cranio-Maxillofacial Surgery. 2014;42(5):588-94.	Excluded by abstract
771	Yadav R, Roychoudhury A, Bhutia O. Mandibular lengthening by distraction osteogenesis in retrognathic temporomandibular joint ankylosis patients suffering from obstructive sleep apnea. Journal of Oral and Maxillofacial Surgery. 2011;69(9):e46-e7.	Excluded by abstract
772	Yadav R, Roychoudhury A. To evaluate the effects of distraction osteogenesis in temporo mandibular joint ankylosis patients suffering from obstructive sleep apnea. International Journal of Oral and Maxillofacial Surgery. 2011;40(10):1038.	Excluded by abstract
773	Yin W, Feng S, Guan W. [Mandibular advancement for the treatment of micrognathia with obstructive sleep apnea]. Zhonghua Zheng Xing Shao Shang Wai Ke Za Zhi. 1994;10(4):265-9.	Excluded by abstract
774	Yoshida K, Sakamoto K, Takagi A, Iizuka T. Three-piece oral appliance with herbst attachments for persistent vegetative state patient with sleep-disordered breathing. Int J Prosthodont. 2003;16(4):350-4.	Excluded by abstract
775	Yoshida K. Effect on blood pressure of oral appliance therapy for sleep apnea syndrome. Int J Prosthodont. 2006;19(1):61-6.	Excluded by abstract
776	Yu B, Ding M, Shi J, Koirala U, Wang W, Ma Q. [Different maxillomandibular advancement methods for the treatment of severe obstructive sleep apneahypopnea syndrome]. Zhonghua Kou Qiang Yi Xue Za Zhi. 2015;50(4):202-5.	Excluded by abstract
777	Yuen H, Rossouw PE, Wolford LM, Wang H. Pharyngeal Airway Space Changes After Condylar Replacement and Mandibular Advancement Surgery. J Oral Maxillofac Surg. 2018;76(6):1165-74.	Excluded by abstract
778	Zakhar A, Wirth C, Farrow E, Tison C, Ferri J, Raoul G. [Surgical treatment of Obstructive Sleep Apnea Syndrome. Functional assessment]. Rev Stomatol Chir Maxillofac Chir Orale. 2014;115(2):79-83.	Excluded by abstract
779	Zeng B, Ng AT, Darendeliler MA, Petocz P, Cistulli PA. Use of flow-volume curves to predict oral appliance treatment outcome in obstructive sleep apnea. Am J Respir Crit Care Med. 2007;175(7):726-30.	Excluded by abstract
780	Zeng B, Ng AT, Qian J, Petocz P, Darendeliler MA, Cistulli PA. Influence of nasal resistance on oral appliance treatment outcome in obstructive sleep apnea. Sleep. 2008;31(4):543-7.	Excluded by abstract
781	Zhou J, Liu YH. A randomised titrated crossover study comparing two oral appliances in the treatment for mild to moderate obstructive sleep apnoea/hypopnoea syndrome. J Oral Rehabil. 2012;39(12):914-22.	Excluded by abstract
782	Куроедова ВД, Чикор ТА, Макарова АН, Ким АА. [Orthodontic treatment effect of on the condition of the upper airways]. Wiad Lek. 2016;69(6):734-736.	Excluded by abstract
783	Aragão W. Respirador bucal (RB). Odontol mod. 1986;13(7):39-41.	Excluded; missing fulltext
784	Godoy Estéves GF. Por qué ortopedia funcional? Rev Asoc Argent Ortop Funcional Maxilares. 1999;30(1):7-26.	Excluded; missing fulltext

785	Godoy Estévez GF, Francesconi MA, Chaile MM. Ortopedia funcional integralista, traumatología y estudios por imágenes. Rev Asoc Argent Ortop Funcional Maxilares. 2012;38(1):2-24.	Excluded; missing fulltext
786	Lorenz de Sarín GI, Galetti S, Sarín Y. Influencia del tratamiento con aparatología ortopédico-funcional sobre la luz del rinofaringe. Rev Asoc Argent Ortop Funcional Maxilares. 2014;40(1):33-8.	Excluded; missing fulltext
787	Lorenz GI, Rivas NH, Ceccarelli A. Las laterodesviaciones mandibulares, fuoncionales y esqueletales. Su resolución desde la ortopedia funcional. Rev Asoc Argent Ortop Funcional Maxilares. 1999;30(1):27-48.	Excluded; missing fulltext
788	Rondeau B. The Rick-A-Nator appliance. Funct Orthod. 1990;7(4):4-6, 8-12.	Excluded; missing fulltext
789	Wang W, Mo SC, Wang L. Changes of airway before and after Twin-block treatment in patients with mandibular retrusion. Shanghai kou qiang yi xue/shanghai journal of stomatology. 2018;27(6):607-11.	Excluded; missing fulltext
790	{NCT} Comparison of Treatment Effects of PowerScope2 and Forsus Using CBCT. Https://clinicaltrialsgov/show/nct03296644 [Internet]. 2017.	Excluded; protocol, ongoing
791	{NCT} Effects of Herbst Appliance Therapy to Improve Airway Dimension. Https://clinicaltrialsgov/show/nct02448017 [Internet]. 2015.	Excluded; protocol, ongoing
792	{NCT} Effects of Orthopedic Mandibular Advancement in Class II Division 1 Malocclusion on Pharyngeal Airway. https://clinicaltrialsgov/show/NCT04255511. 2020.	Excluded; protocol, ongoing
793	{NCT} Upper Airway's Pressure Drop Analyses After Mandibular Advancement and Maxillary Expansion. https://clinicaltrialsgov/show/NCT04190953. 2019.	Excluded; protocol, ongoing
794	{ISRCTN} Effects of different functional therapy protocols on airway dimensions during Herbst appliance therapy. http://wwwwhoint/trialsearch/Trial2aspx?TrialID=ISRCTN69743543. 2018.	Excluded; protocol, published
795	Bernkopf E, Broia V, Bertarini AM, Polcino P, Macri F. Oral breathing and malocclusion. Medico e Bambino. 2002;21(2):107-12.	Excluded; non-clinical study
796	Champagne M. Brainstorming. Int J Orthod Milwaukee. 2005;16(4):34-7.	Excluded; non-clinical study
797	Champagne M. Time passes and changes come. Int J Orthod Milwaukee. 2008;19(4):5-12.	Excluded; non-clinical study
798	Li X, Long H, Lai W. Effect of Herbst appliance on upper airway changes in orthodontic patients. American Journal of Orthodontics and Dentofacial Orthopedics. 2020;158(1):5-6.	Excluded; non-clinical study
799	Oliveira PM, Cheib-Vilefort PL, Gontijo HdP, Souki BQ, Melgaco CA, Franchi L, et al. Effect of Herbst appliance on upper airway changes in orthodontic patients Response. American Journal of Orthodontics and Dentofacial Orthopedics. 2020;158(1):6-7.	Excluded; non-clinical study
800	Owen Iii AH. Functional appliances-a valuable tool in your armamentarium. American Journal of Orthodontics and Dentofacial Orthopedics. 1991;100(4):31A-3A.	Excluded; non-clinical study
801	Padmanabhan S. Effect of functional appliances on the airway in Class II malocclusions. Journal of the World Federation of Orthodontists. 2020;9(3):S27-S30.	Excluded; non-clinical study
802	Rana M, August J, Levi J, Parsi G, Motro M, Debassio W. Alternative Approaches to Adenotonsillectomy and Continuous Positive Airway Pressure (CPAP) for the Management of Pediatric Obstructive Sleep Apnea (OSA): A Review. Sleep Disorders. 2020;2020.	Excluded; non-clinical study
803	Anusuya V, Jena AK, Sharan J. Effects of functional appliance treatment on pharyngeal airway passage dimensions in Class II malocclusion subjects with retrognathic mandibles: A systematic review. Apos Trends in Orthodontics. 2019;9(3):138-48.	Excluded; review
804	Mohamed RN, Basha S, Al-Thomali Y. Changes in upper airway dimensions following orthodontic treatment of skeletal Class II malocclusion with twin block appliance: A systematic review. Turkish Journal of Orthodontics. 2020;33(1):59-64.	Excluded; review
805	Santana LG, Avelar K, Flores-Mir C, Marques LS. Incremental or maximal mandibular advancement in the treatment of class II malocclusion through functional appliances: A systematic review with meta-analysis. Orthodontics and Craniofacial Research. 2020.	Excluded; review
806	Xiang M, Hu B, Liu Y, Sun J, Song J. Changes in airway dimensions following functional appliances in growing patients with skeletal class II malocclusion: A systematic review and meta-analysis. Int J Pediatr Otorhinolaryngol. 2017;97:170-80.	Excluded; review
807	Bignotti D, De Stefani AD, Mezzofranco L, Bruno G, Gracco A. Multidisciplinary approach in a 12-year-old patient affected by severe obstructive sleep apnea: A case-report. Sleep Medicine Research. 2019;10(2):103-7.	Excluded; case report / series
808	Chakraborti A, Castillo M, Zahid E. A CASE OF OSA IN A PATIENT WITH A VENTRICULO-PERITONEAL SHUNT: A REAL HEADACHE. Chest. 2020;158(4):A2318.	Excluded; case report / series
809	Conaway JR, Scherr SC. Multidisciplinary management of the airway in a trauma-induced brain injury patient. Sleep Breath. 2004;8(3):165-70.	Excluded; case report / series
810	De Stefani A, Bruno G, Agostini L, Mezzofranco L, Gracco A. Resolution of a severe grade of obstructive sleep apnea syndrome with mandibular advancement device: A case report. Sleep Medicine Research. 2020;11(1):44-8.	Excluded; case report / series
811	Ebato A, Suzuki H, Sakamaki T, Ooguchi S, Chow CM, Komiyama O. Obstructive sleep apnea treatment with a twopiece mandibular advancement device with an elastic retention band in combination with orofacial myofunctional therapy: A case report. Sleep Science. 2019;12(1):57-60.	Excluded; case report / series
812	Ebrahimzadeh P, Almodovar K, Tablizo MA. A treatment of lacrimal duct air regurgitation in a patient intolerant to PAP therapy. Sleep. 2019;42:A419.	Excluded; case report / series
813	Faria PR. A utilização da Ortodontia Miofuncional no tratamento de crianças e adolescentes. Ortho Sci, Orthod sci pract. 2014;7(27):401-6.	Excluded; case report / series
814	Galeotti A, Festa P, Pavone M, De Vincentiis GC. Effects of simultaneous palatal expansion and mandibular advancement in a child suffering from OSA. Acta Otorhinolaryngol Ital. 2016;36(4):328-32.	Excluded; case report / series
815	Garcez AS, Suzuki SS, Storto CJ, Cusmanich KG, Elkenawy I, Moon W. Effects of maxillary skeletal expansion on respiratory function and sport performance in a para-athlete - A case report. Phys Ther Sport. 2019;36:70-7.	Excluded; case report / series
816	Lee K-H, Kim K-A, Kwon Y-D, Kim S-W, Kim S-J. Maxillomandibular advancement surgery after long-term use of a mandibular advancement device in a post-adolescent patient with obstructive sleep apnea. Korean Journal of Orthodontics. 2019;49(4):265-76.	Excluded; case report / series
817	Li X, Wang H, Li S, Bai Y. Treatment of a Class II Division 1 malocclusion with the combination of a myofunctional trainer and fixed appliances. American Journal of Orthodontics and Dentofacial Orthopedics. 2019;156(4):545-54.	Excluded; case report / series
818	Rose E, Schessl J. Orthodontic procedures in the treatment of obstructive sleep apnea in children. J Orofac Orthop. 2006;67(1):58-67.	Excluded; case report / series
819	Singh GD, Kim H. Upper oropharyngeal airway changes in Korean adults following biomimetic oral appliance therapy. Sleep Medicine. 2019;64:S353-S4.	Excluded; case report / series
820	Acar M, Saylisoy S, San T, Cingi C, Ay Y, Karabag A, et al. Comparison of the effects of mandibular protruding devices on obstructive sleep apnoea patients and healthy volunteers. J Craniomaxillofac Surg. 2014;42(7):1465-8.	Excluded; obstructive sleep apnea
821	AlSaty G, Xiang J, Burns M, Eliliwi M, Palomo JM, Martin C, et al. Follow-up observation of patients with obstructive sleep apnea treated by maxillomandibular advancement. Am J Orthod Dentofacial Orthop. 2020;158(4):527-34.	Excluded; obstructive sleep apnea
822	Ash S. A chrome cobalt mandibular advancement appliance for snoring and obstructive sleep apnoea used during active aligner orthodontic treatment. Journal of orthodontics. 2020;47(2):181-4.	Excluded; obstructive sleep apnea
823	Bamagoos AA, Cistulli PA, Sutherland K, Ngiam J, Burke PGR, Bilston LE, et al. Dose-dependent effects of mandibular advancement on upper airway collapsibility and muscle function in obstructive sleep apnea. Sleep. 2019;42(6).	Excluded; obstructive sleep apnea
824	Bartolucci ML, Bortolotti F, Martina S, Corazza G, Michelotti A, Alessandri-Bonetti G. Dental and skeletal long-term side effects of mandibular advancement devices in obstructive sleep apnea patients: A systematic review with meta-regression analysis. European Journal of Orthodontics. 2019;41(1):89-100.	Excluded; obstructive sleep apnea

825	Battagel JM, Johal A, L'Estrange PR, Croft CB, Kotecha B. Changes in airway and hyoid position in response to mandibular protrusion in subjects with obstructive sleep apnoea (OSA). Eur J Orthod. 1999;21(4):363-76.	Excluded; obstructive sleep apnea
826	Bernhold M, Bondemark L. A magnetic appliance for treatment of snoring patients with and without obstructive sleep apnea. American Journal of Orthodontics and Dentofacial Orthopedics. 1998;113(2):144-55.	Excluded; obstructive sleep apnea
827	Bonham PE, Currier GF, Orr WC, Othman J, Nanda RS. The effect of a modified functional appliance on obstructive sleep apnea. Am J Orthod Dentofacial Orthop. 1988;94(5):384-92.	Excluded; obstructive sleep apnea
828	Buller M, Jodeh DS, Rottgers SA. Maxillomandibular Advancement for the Treatment of Obstructive Sleep Apnea in Patients With Normal or Class I Malocclusion. J Craniofac Surg. 2020;31(3):716-9.	Excluded; obstructive sleep apnea
829	Chan AS, Sutherland K, Schwab RJ, Zeng B, Petocz P, Lee RW, et al. The effect of mandibular advancement on upper airway structure in obstructive sleep apnoea. Thorax. 2010;65(8):726-32.	Excluded; obstructive sleep apnea
830	Chan ASL, Sutherland K, Schwab RJ, Zeng B, Petocz P, Lee RWW, et al. The effect of mandibular advancement splints on upper airway anatomy in obstructive sleep apnoea. Sleep and Biological Rhythms. 2009;7:A17.	Excluded; obstructive sleep apnea
831	Chen H, Aarab G, Lobbezoo F, De Lange J, Van der Stelt P, Darendeliler MA, et al. Differences in three-dimensional craniofacial anatomy between responders and non-responders to mandibular advancement splint treatment in obstructive sleep apnoea patients. Eur J Orthod. 2019.	Excluded; obstructive sleep apnea
832	Choi JK, Hur YK, Lee JM, Clark GT. Effects of mandibular advancement on upper airway dimension and collapsibility in patients with obstructive sleep apnea using dynamic upper airway imaging during sleep. Oral Surg Oral Med Oral Pathol Oral Radiol Endod. 2010;109(5):712-9.	Excluded; obstructive sleep apnea
833	Cossellu G, Biagi R, Sarcina M, Mortellaro C, Farronato G. Three-dimensional evaluation of upper airway in patients with obstructive sleep apnea syndrome during oral appliance therapy. J Craniofac Surg. 2015;26(3):745-8.	Excluded; obstructive sleep apnea
834	Cozza P, Ballanti F, Castellano M, Fanucci E. Role of computed tomography in the evaluation of orthodontic treatment in adult patients with obstructive sleep apnea syndrome (OSA). Prog Orthod. 2008;9(1):6-16.	Excluded; obstructive sleep apnea
835	Cunha TCA, Guimaraes TM, Schultz TCB, Almeida FR, Cunha TM, Simamoto PCJ, et al. Predictors of success for mandibular repositioning appliance in obstructive sleep apnea syndrome. Braz Oral Res. 2017;31:e37.	Excluded; obstructive sleep apnea
836	Cupido GF, Gelardi M, La Mantia I, Aragona SE, Vicini C, Ciprandi G, et al. Broncalt®, class II medical device, in patients with acute upper airways disease: a survey in clinical practice. Acta Biomed. 2019;90(7-s):24-9.	Excluded; obstructive sleep apnea
837	Cupido GF, Gelardi M, La Mantia I, Aragona SE, Vicini C, Ciprandi G, et al. Broncalt®, class II medical device, in patients with chronic relapsed upper airways disease: a survey in clinical practice. Acta Biomed. 2019;90(7-s):36-40.	Excluded; obstructive sleep apnea
838	Cupido GF, Gelardi M, La Mantia I, Aragona SE, Vicini C, Ciprandi G, et al. Broncalt®, class II medical device, in patients with chronic upper airways disease: a survey in clinical practice. Acta Biomed. 2019;90(7-s):30-5.	Excluded; obstructive sleep apnea
839	de Ruiter MHT, Apperloo RC, Milstein DMJ, de Lange J. Assessment of obstructive sleep apnoea treatment success or failure after maxillomandibular advancement. Int J Oral Maxillofac Surg. 2017;46(11):1357-62.	Excluded; obstructive sleep apnea
840	Edwards B, Andara C, Landry S, Sands S, Joosten S, Owens R, et al. Upper-Airway Collapsibility and Loop Gain Predict the Response to Oral Appliance Therapy in Patients with Obstructive Sleep Apnea. American journal of respiratory and critical care medicine. 2016; 194(11):1413-22.	Excluded; obstructive sleep apnea
841	Fluger KR, Zhou A, Al-Jewair T. Low-Quality Evidence Supports the Use of Mandibular Advancement Appliances in Managing Obstructive Sleep Apnea in Children. J Evid Based Dent Pract. 2020;20(1):101411.	Excluded; obstructive sleep apnea
842	Fransson AMC, Benavente-Lundahl C, Isacsson G. A prospective 10-year cephalometric follow-up study of patients with obstructive sleep apnea and snoring who used a mandibular protruding device. American Journal of Orthodontics and Dentofacial Orthopedics. 2020;157(1):91-7.	Excluded; obstructive sleep apnea
843	Gale DJ, Sawyer RH, Woodcock A, Stone P, Thompson R, O'Brien K. Do oral appliances enlarge the airway in patients with obstructive sleep apnoea? A prospective computerized tomographic study. Eur J Orthod. 2000;22(2):159-68.	Excluded; obstructive sleep apnea
844	Geoghegan F, Ahrens A, McGrath C, Hagg U. An evaluation of two different mandibular advancement devices on craniofacial characteristics and upper airway dimensions of Chinese adult obstructive sleep apnea patients. Angle Orthod. 2015;85(6):962-8.	Excluded; obstructive sleep apnea
845	Huang YS, Chuang LC, Hervy-Auboiron M, Paiva T, Lin CH, Guilleminault C. Neutral supporting mandibular advancement device with tongue bead for passive myofunctional therapy: a long term follow-up study. Sleep Med. 2019;60:69-74.	Excluded; obstructive sleep apnea
846	Isacsson G, Nohlert E, Fransson AMC, Bornefalk-Hermansson A, Wiman Eriksson E, Ortlieb E, et al. Use of bibloc and monobloc oral appliances in obstructive sleep apnoea: a multicentre, randomized, blinded, parallel-group equivalence trial. European journal of orthodontics. 2019;41(1):80-8.	Excluded; obstructive sleep apnea
847	Ishida M, Inoue Y, Suto Y, Okamoto K, Ryoke K, Higami S, et al. Mechanism of action and therapeutic indication of prosthetic mandibular advancement in obstructive sleep apnea syndrome. Psychiatry Clin Neurosci. 1998;52(2):227-9.	Excluded; obstructive sleep apnea
848	Ishiyama H, Hasebe D, Sato K, Sakamoto Y, Furuhashi A, Komori E, et al. The Efficacy of Device Designs (Mono-block or Bi-block) in Oral Appliance Therapy for Obstructive Sleep Apnea Patients: A Systematic Review and Meta-Analysis. International Journal of Environmental Research and Public Health. 2019;16(17).	Excluded; obstructive sleep apnea
849	Johal A, Sheriteh Z, Battagel J, Marshall C. The use of videofluoroscopy in the assessment of the pharyngeal airway in obstructive sleep apnoea. Eur J Orthod. 2011;33(2):212-9.	Excluded; obstructive sleep apnea
850	Kissmann G, Leal R, Franco CAdB. Apneia obstrutiva do sono: da suspeita diagnóstica ao tratamento. J bras med. 2013;101(3):37-43.	Excluded; obstructive sleep apnea
851	Maspero C, Giannini L, Galbiati G, Kairyte L, Farronato G. Upper airway obstuction in class II patients. Effects of Andresen activator on the anatomy of pharingeal airway passage. Cone beam evalution. Stomatologija. 2015;17(4):124-30.	Excluded; obstructive sleep apnea
852	Matsumura Y, Ueda H, Nagasaki T, Medina CC, Iwai K, Tanimoto K. Multislice Computed Tomography Assessment of Airway Patency Changes Associated with Mandibular Advancement Appliance Therapy in Supine Patients with Obstructive Sleep Apnea. Sleep Disorders. 2019;2019.	Excluded; obstructive sleep apnea
853	Mayer G, Meier-Ewert K. Cephalometric predictors for orthopaedic mandibular advancement in obstructive sleep apnoea. European Journal of Orthodontics. 1995;17(1):35-43.	Excluded; obstructive sleep apnea
854	Mostafiz WR, Carley DW, Viana MGC, Ma S, Dalci O, Darendeliler MA, et al. Changes in sleep and airway variables in patients with obstructive sleep apnea after mandibular advancement splint treatment. Am J Orthod Dentofacial Orthop. 2019;155(4):498-508.	Excluded; obstructive sleep apnea
855	Nakai T, Matsuo A, Takata Y, Usui Y, Kitamura K, Chikazu D. Role of dental sleep medicine in management of patients with obstructive sleep apnea disorders using a team approach. Acta Odontol Scand. 2018;76(8):605-11.	Excluded; obstructive sleep apnea
856	Ogutcen-Toller M, Sarac YS, Cakír-Ozkan N, Sarac D, Sakan B. Computerized tomographic evaluation of effects of mandibular anterior repositioning on the upper airway: A pilot study. Journal of Prosthetic Dentistry. 2004;92(2):184-9.	Excluded; obstructive sleep apnea
857	Patel S, Rinchuse D, Zullo T, Wadhwa R. Long-term dental and skeletal effects of mandibular advancement devices in adults with obstructive sleep apnoea: A systematic review. International Orthodontics. 2019;17(1):3-11.	Excluded; obstructive sleep apnea
858	Pavoni C, Cretella Lombardo E, Lione R, Bollero P, Ottaviani F, Cozza P. Orthopaedic treatment effects of functional therapy on the sagittal pharyngeal dimensions in subjects with sleep-disordered breathing and Class II malocclusion. Acta Otorhinolaryngol Ital. 2017;37(6):479-85.	Excluded; obstructive sleep apnea
859	Piskin B, Karakoc O, Genc H, Akay S, Sipahi C, Erdem M, et al. Effects of varying mandibular protrusion and degrees of vertical opening on upper airway dimensions in apneic dentate subjects. J Orofac Orthop. 2015;76(1):51-65.	Excluded; obstructive sleep apnea
860	Proothi M, Grazina VJR, Gold AR. Chronic insomnia remitting after maxillomandibular advancement for mild obstructive sleep apnea: a case series. J Med Case Rep. 2019;13(1):252.	Excluded; obstructive sleep apnea
861	Remy F, Bonnaure P, Moisdon P, Burgart P, Godio-Raboutet Y, Thollon L, et al. Preliminary results on the impact of simultaneous palatal expansion and mandibular advancement on the respiratory status recorded during sleep in OSAS children. Journal of Stomatology, Oral and Maxillofacial Surgery. 2020.	Excluded; obstructive sleep apnea

862	Sakamoto Y, Furuhashi A, Komori E, Ishiyama H, Hasebe D, Sato K, et al. The Most Effective Amount of Forward Movement for Oral Appliances for Obstructive Sleep Apnea: A Systematic Review. Int J Environ Res Public Health. 2019;16(18).	Excluded; obstructive sleep apnea
863	Silva FBNN, Cavalieri-Pereira L, Pedroso-Oliveira G, Rocha JLS, Brancher GQB. Orthognathic surgery with mandibular osteotomy modified for OSAS treatment in class II patient. International Journal of Oral and Maxillofacial Surgery. 2019;48:277.	Excluded; obstructive sleep apnea
864	Singh GD, Heit T. Changes is sleep parameters following short-term biomimetic oral appliance therapy for obstructive sleep apnea in adults. Sleep medicine. 2019;64:S353-.	Excluded; obstructive sleep apnea
865	Spyrides GM, Spyrides SMM, Roizman E, Corpas L, Zagury R, Semeone PN. Apnéia e ronco tratados com placa oclusais. J bras ortodon ortop facial. 2000;5(25):43-54.	Excluded; obstructive sleep apnea
866	Stache R, Prasad B, Viana G, Galang-Boquiren MT. Oral appliance and pharmacologic agents in treatment of sleep apnea. Sleep. 2019;42:A224.	Excluded; obstructive sleep apnea
867	Sutherland K, Chan ASL, Ngiam J, Darendeliler MA, Cistulli PA. Qualitative assessment of awake nasopharyngoscopy for prediction of oral appliance treatment response in obstructive sleep apnoea. Sleep Breath. 2018;22(4):1029-36.	Excluded; obstructive sleep apnea
868	Umemoto G, Toyoshima H, Yamaguchi Y, Aoyagi N, Yoshimura C, Funakoshi K. Therapeutic Efficacy of Twin-Block and Fixed Oral Appliances in Patients with Obstructive Sleep Apnea Syndrome. J Prosthodont. 2019;28(2):e830-e6.	Excluded; obstructive sleep apnea
869	Van der Cruyssen F, Vuylsteke P, Claerhout M, Vanden Bulcke M, Timmermans L, Delsupehe K. The effect of maxillary-mandibular advancement surgery on two-dimensional cephalometric analysis, polysomnographic and patient-reported outcomes in 32 patients with sleep disordered breathing: A retrospective cohort study. Oral and Maxillofacial Surgery Cases. 2019;5(3).	Excluded; obstructive sleep apnea
870	Vecchierini MF, Attali V, Collet JM, d'Ortho MP, Goutorbe F, Kerbrat JB, et al. Sex differences in mandibular repositioning device therapy effectiveness in patients with obstructive sleep apnea syndrome. Sleep and Breathing. 2019;23(3):837-48.	Excluded; obstructive sleep apnea
871	Wadi MHA, Vargas Neto J, Vedovello Filho M, Nouer PRdA, Sallum EJ. Placas oclusais no tratamento da síndrome da apnéia obstrutiva do sono: uma alternativa conservadora. Ortodontia. 2002;35(2):137-44.	Excluded; obstructive sleep apnea
872	Wu Q, Wang Y, Wang P, Xiang Z, Ye B, Li J. The inverted-L ramus osteotomy versus sagittal split ramus osteotomy in maxillomandibular advancement for the treatment of obstructive sleep apnea patients: A retrospective study. Journal of Cranio-Maxillofacial Surgery. 2019;47(12):1839-47.	Excluded; obstructive sleep apnea
873	Yanyan M, Min Y, Xuemei G. Mandibular advancement appliances for the treatment of obstructive sleep apnea in children: a systematic review and meta-analysis. Sleep Med. 2019;60:145-51.	Excluded; obstructive sleep apnea
874	Zhang M, Liu Y, Liu Y, Yu F, Yan S, Chen L, et al. Effectiveness of oral appliances versus continuous positive airway pressure in treatment of OSA patients: An updated meta-analysis. Cranio. 2019;37(6):347-64.	Excluded; obstructive sleep apnea
875	Cretella Lombardo E, Franchi L, Lione R, Chiavaroli A, Cozza P, Pavoni C. Evaluation of sagittal airway dimensions after face mask therapy with rapid maxillary expansion in Class III growing patients. Int J Pediatr Otorhinolaryngol. 2020;130:109794.	Excluded; no Class II
876	da Silva Machado V, Cantharino de Carvalho BA, Vedovello SAS, Valdrighi H, Santamaria Júnior M. Pharyngeal Airway Modifications in Skeletal Class III Patients Undergoing Bimaxillary Advancement Surgery. J Oral Maxillofac Surg. 2019;77(10):2126.e1-.e8.	Excluded; no Class II
877	Fareen N, Alam MK, Khamis MF, Mokhtar N. Treatment effects of two different appliances on pharyngeal airway space in mixed dentition Malay children. Int J Pediatr Otorhinolaryngol. 2019;125:159-63.	Excluded; no Class II
878	Hiyama S, Tsuiki S, Ono T, Kuroda T, Ohyama K. Effects of mandibular advancement on supine airway size in normal subjects during sleep. Sleep. 2003;26(4):440-5.	Excluded; no Class II
879	Radescu OD, Colosi HA, Albu S. Effects of rapid palatal expansion (RPE) and twin block mandibular advancement device (MAD) on pharyngeal structures in Class II pediatric patients from Cluj-Napoca, Romania. Cranio. 2018:1-8.	Excluded; no Class II
880	Alcalde LFA, Faria PEP, Nogueira RLM, Chihara L, Sant'Ana E. Computed tomography visualizing alterations in the upper airway after orthognathic surgery. Journal of Cranio-Maxillofacial Surgery. 2019;47(7):1041-5.	Excluded; no functional appliance
881	An JH, Park SB, Choi YK, Lee SH, Kim KB, Kim YI. Cone-Beam Computed Tomography Evaluation of Pharyngeal Airway Space Changes After Bimaxillary Orthognathic Surgery in Patients With Class III Skeletal Deformities: A 6-Year Follow-Up Study. Journal of Oral and Maxillofacial Surgery. 2019;77(12):2534-44.	Excluded; no functional appliance
882	Bellerive A, Montpetit A, El-Khatib H, Carra MC, Remise C, Desplats E, et al. The effect of rapid palatal expansion on sleep bruxism in children. Sleep and breathing. 2015;19(4):1265-71.	Excluded; no functional appliance
883	Chen Y, Zhang J, Rao N, Han Y, Ferraro N, August M. Independent risk factors for long-term skeletal relapse after mandibular advancement with bilateral sagittal split osteotomy. International Journal of Oral and Maxillofacial Surgery. 2020;49(6):779-86.	Excluded; no functional appliance
884	Cheng-Hui Lin C, Wang PF, Ray Han Loh S, Lau HT, Sheng-Ping Hsu S. Maxillomandibular Rotational Advancement: Airway, Aesthetics, and Angle's Considerations. Sleep Med Clin. 2019;14(1):83-9.	Excluded; no functional appliance
885	do Vale F, Rodrigues ML, Francisco I, Roseiro A, Santos I, Caramelo F, et al. Short-term pharyngeal airway space changes after mandibular advancement surgery in Class II patients-a two-dimensional retrospective study. Orthod Craniofac Res. 2019;22(2):81-6.	Excluded; no functional appliance
886	do Vale F, Rodrigues ML, Francisco I, Roseiro A, Santos I, Caramelo F, et al. Short-term pharyngeal airway space changes after mandibular advancement surgery in Class II patients—a two-dimensional retrospective study. Orthodontics and Craniofacial Research. 2019;22(2):81-6.	Excluded; no functional appliance
887	Foltan R, Rybinova K. The impact of mandibular advancement on the upper airway patterns--cephalometric study. Prague Med Rep. 2007;108(2):147-54.	Excluded; no functional appliance
888	Furche S, Edwards SP, Aronovich S, Hummon G, Shah KB, Conley RS. 3D Airway changes using cone beam computed tomography in patients following mandibular advancement surgery with and without constriction. Orthod Craniofac Res. 2019;22 Suppl 1:36-42.	Excluded; no functional appliance
889	Gurani SF, Di Carlo G, Thorn JJ, Ingerslev J, Cattaneo PM, Pinholt EM. Two-Year Postoperative Upper Airway Cone-Beam Computed Tomographic Outcomes Based on a Verified Upper Airway Analysis Following Bimaxillary Orthognathic Surgery. Journal of Oral and Maxillofacial Surgery. 2019;77(7):1435-45.	Excluded; no functional appliance
890	Havron AG, Aronovich S, Shelgikar AV, Kim HL, Conley RS. 3D Airway changes using CBCT in patients following mandibular setback surgery ± maxillary advancement. Orthod Craniofac Res. 2019;22 Suppl 1:30-5.	Excluded; no functional appliance
891	Kapadia H, Susarla S, Hopper R. Orthodontic considerations in combined orthodontic-surgical management of achondroplasia. Cleft Palate-Craniofacial Journal. 2019;56(1):125.	Excluded; no functional appliance
892	Kiliç B, Amuk NG, Hacioğlu Z, Demirbaş AE. The Effect of Bimaxillary Surgery on the Airway Morphology in Class III Patients: Taking Into Account the Head Posture During Walking. J Craniofac Surg. 2019;30(6):1686-91.	Excluded; no functional appliance
893	Nishanth R, Sinha R, Paul D, Uppada UK, Rama Krishna BV, Tiwari P. Evaluation of Changes in the Pharyngeal Airway Space as a Sequele to Mandibular Advancement Surgery: A Cephalometric Study. Journal of Maxillofacial and Oral Surgery. 2020;19(3):407-13.	Excluded; no functional appliance
894	Niskanen I, Kurimo J, Järnstedt J, Himanen SL, Helminen M, Peltomäki T. Effect of Maxillomandibular Advancement Surgery on Pharyngeal Airway Volume and Polysomnography Data in Obstructive Sleep Apnea Patients. J Oral Maxillofac Surg. 2019;77(8):1695-702.	Excluded; no functional appliance
895	Okuno K, Furuhashi A, Nakamura S, Suzuki H, Arisaka T, Taga H, et al. Japanese Cross-Sectional Multicenter Survey (JAMS) of Oral Appliance Therapy in the Management of Obstructive Sleep Apnea. Int J Environ Res Public Health. 2019;16(18).	Excluded; no functional appliance
896	Rückschloß T, Ristow O, Berger M, Engel M, Freudlsperger C, Hoffmann J, et al. Relations between mandible-only advancement surgery, the extent of the posterior airway space, and the position of the hyoid bone in Class II patients: a three-dimensional analysis. Br J Oral Maxillofac Surg. 2019;57(10):1032-8.	Excluded; no functional appliance
897	Sahoo NK, Agarwal SS, Datana S, Bhandari SK. Effect of Mandibular Advancement Surgery on Tongue Length and Height and Its Correlation with Upper Airway Dimensions. Journal of Maxillofacial and Oral Surgery. 2020;19(4):624-9.	Excluded; no functional appliance
898	Tsui WK, Yang Y, McGrath C, Leung YY. Improvement in quality of life after skeletal advancement surgery in patients with moderate-to-severe obstructive sleep apnoea: a longitudinal study. International Journal of Oral and Maxillofacial Surgery. 2020;49(3):333-41.	Excluded; no functional appliance
899	Tsui WK, Yang Y, McGrath C, Leung YY. Mandibular distraction osteogenesis versus sagittal split ramus osteotomy in managing obstructive sleep apnea: A randomized clinical trial. J Craniomaxillofac Surg. 2019;47(5):750-7.	Excluded; no functional appliance

900	Wiedemeyer V, Berger M, Martini M, Kramer FJ, Heim N. Predictability of pharyngeal airway space dimension changes after orthognathic surgery in class II patients: A mathematical approach. J Craniomaxillofac Surg. 2019;47(10):1504-9.	Excluded; no functional appliance
901	Afzal E, Fida M. Evaluation of the change in the tongue posture and in the hyoid bone position after Twin Block appliance therapy in skeletal class II subjects. Dental and Medical Problems. 2019;56(4):379-84.	Excluded; no control group
902	Ali B, Shaikh A, Fida M. CHANGES IN ORO-PHARYNGEAL AIRWAY DIMENSIONS AFTER TREATMENT WITH FUNCTIONAL APPLIANCE IN CLASS II SKELETAL PATTERN. J Ayub Med Coll Abbottabad. 2015;27(4):759-63.	Excluded; no control group
903	Arora V, Sharma R, Chowdhary S. Comparative evaluation of treatment effects between two fixed functional appliances for correction of Class II malocclusion: A single-center, randomized controlled trial. Angle Orthod. 2018 May;88(3):259-266.	Excluded; no control group
904	Celikoglu M, Buyuk SK, Ekizer A, Unal T. Pharyngeal airway effects of Herbst and skeletal anchored Forsus FRD EZ appliances. Int J Pediatr Otorhinolaryngol. 2016;90:23-8.	Excluded; no control group
905	Ciavarella D, Lo Russo L, Mastrovincenzo M, Padalino S, Montaruli G, Giannatempo G, et al. Cephalometric evaluation of tongue position and airway remodelling in children treated with swallowing occlusal contact intercept appliance (S.O.C.I.A.). Int J Pediatr Otorhinolaryngol. 2014;78(11):1857-60.	Excluded; no control group
906	Dalla Torre D, Burtscher D, Widmann G, Rasse M, Puelacher T, Puelacher W. Long-term influence of mandibular advancement on the volume of the posterior airway in skeletal Class II-patients: a retrospective analysis. Br J Oral Maxillofac Surg. 2017;55(8):780-6.	Excluded; no control group
907	Erbas B, Kocadereli I. Upper airway changes after Xbow appliance therapy evaluated with cone beam computed tomography. Angle Orthod. 2014;84(4):693-700.	Excluded; no control group
908	Fantini SMd, Andrighetto AR. Estudo da correlação entre a alteração do espaço aéreo orofaríngeo e as mudanças da posição natural da cabeça e da altura facial ânter-inferior em indivíduos assintomáticos, com maloclusões de classe II de angle, submetidos à desprogramação neuromuscular com placa oclusal. Ortodontia. 2004;37(2):14-21.	Excluded; no control group
909	Godt A, Koos B, Hagen H, Goz G. Changes in upper airway width associated with Class II treatments (headgear vs activator) and different growth patterns. Angle Orthod. 2011;81(3):440-6.	Excluded; no control group
910	Gómez SPP. Tomografia computadorizada de feixe cônico para avaliação do tratamento da classe II divisão 1ª com o aparelho Herbst no espaço aéreo faríngeo. 2010:95-.	Excluded; no control group
911	Gopal R, Tripathi T, Rai P, Kanase A. Three-dimensional assessment of pharyngeal airway space by MRI in class II division 1 patients treated by twin block appliance. Journal of Clinical and Diagnostic Research. 2018;12(9):ZC20-ZC3.	Excluded; no control group
912	Gu M, Savoldi F, Hägg U, McGrath CPJ, Wong RWK, Yang Y. Upper Airway Changes following Functional Treatment with the Headgear Herbst or Headgear Twin Block Appliance Assessed on Lateral Cephalograms and Magnetic Resonance Imaging. ScientificWorldJournal. 2019;2019:1807257.	Excluded; no control group
913	Gul Amuk N, Kurt G, Baysal A, Turker G. Changes in pharyngeal airway dimensions following incremental and maximum bite advancement during Herbst-rapid palatal expander appliance therapy in late adolescent and young adult patients: a randomized non-controlled prospective clinical study. Eur J Orthod. 2019;41(3):322-30.	Excluded; no control group
914	Hourfar J, Kinzinger GS, Meissner LK, Lisson JA. Effects of two different removable functional appliances on depth of the posterior airway space : A retrospective cephalometric study. J Orofac Orthop. 2017;78(2):166-75.	Excluded; no control group
915	Isidor S, Di Carlo G, Cornelis MA, Isidor F, Cattaneo PM. Three-dimensional evaluation of changes in upper airway volume in growing skeletal Class II patients following mandibular advancement treatment with functional orthopedic appliances. Angle Orthod. 2018;88(5):552-9.	Excluded; no control group
916	Julku J, Pirilä-Parkkinen K, Pirttiniemi P. Airway and hard tissue dimensions in children treated with early and later timed cervical headgear- a randomized controlled trial. European journal of orthodontics. 2018; 40(3):285-95.	Excluded; no control group
917	Kinzinger G, Czapka K, Ludwig B, Glasl B, Gross U, Lisson J. Effects of fixed appliances in correcting Angle Class II on the depth of the posterior airway space: FMA vs. Herbst appliance--a retrospective cephalometric study. J Orofac Orthop. 2011;72(4):301-20.	Excluded; no control group
918	Koay WL, Yang Y, Tse CS, Gu M. Effects of Two-Phase Treatment with the Herbst and Preadjusted Edgewise Appliances on the Upper Airway Dimensions. ScientificWorldJournal. 2016;2016:4697467.	Excluded; no control group
919	Lin YC, Lin HC, Tsai HH. Changes in the Pharyngeal Airway and Position of the Hyoid Bone After Treatment With a Modified Bionator in Growing Patients With Retrognathia. Journal of Experimental and Clinical Medicine. 2011;3(2):93-8.	Excluded; no control group
920	Manni A, Pasini M, Giuca MR, Morganti R, Cozzani M. A retrospective cephalometric study on pharyngeal airway space changes after rapid palatal expansion and Herbst appliance with or without skeletal anchorage. Prog Orthod. 2016;17(1):29.	Excluded; no control group
921	Ozdemir F, Ulkur F, Nalbantgil D. Effects of fixed functional therapy on tongue and hyoid positions and posterior airway. Angle Orthod. 2014;84(2):260-4.	Excluded; no control group
922	Peterson ST. A CBCT Study of Pharyngeal Airway Changes Due to Fixed Functional Appliances. MSc Thesis, Loma Linda University, 2016.	Excluded; no control group
923	Rădescu OD, Colosi HA, Albu S. Effects of rapid palatal expansion (RPE) and twin block mandibular advancement device (MAD) on pharyngeal structures in Class II pediatric patients from Cluj-Napoca, Romania. Cranio. 2020;38(1):22-9.	Excluded; no control group
924	Ravera S, Rollet D, Cugliari G, Deregibus A, Castroflorio T. Interceptive treatment effects of EF preformed appliance in pre-pubertal and pubertal skeletal Class II growing patients: A retrospective controlled study. European Journal of Paediatric Dentistry. 2020;21(2):123-8.	Excluded; no control group
925	Restrepo C, Santamaria A, Pelaez S, Tapias A. Oropharyngeal airway dimensions after treatment with functional appliances in class II retrognathic children. J Oral Rehabil. 2011;38(8):588-94.	Excluded; no control group
926	Schutz TC, Dominguez GC, Hallinan MP, Cunha TC, Tufik S. Class II correction improves nocturnal breathing in adolescents. Angle Orthod. 2011;81(2):222-8.	Excluded; no control group
927	Shetty K, Saidath K, Shetty A, Ravi MS, Shashidhar K, Anushree A. Assessment and comparison of oropharyngeal airway dimensions in skeletal Class II cases treated with forsus FRD and Twin Block Appliances. Nitte University Journal of Health Science. 2017;7(2):8-18.	Excluded; no control group
928	Singh GD, Garcia-Motta AV, Hang WM. Evaluation of the posterior airway space following Biobloc therapy: geometric morphometrics. Cranio. 2007;25(2):84-9.	Excluded; no control group
929	Smailiene D, Intiene A, Dobradziejute I, Kusleika G. Effect of Treatment with Twin-Block Appliances on Body Posture in Class II Malocclusion Subjects: A Prospective Clinical Study. Med Sci Monit. 2017;23:343-52.	Excluded; no control group
930	Temani P, Jain P, Rathee P, Temani R. Volumetric changes in pharyngeal airway in Class II division 1 patients treated with Forsus-fixed functional appliance: A three-dimensional cone-beam computed tomography study. Contemp Clin Dent. 2016;7(1):31-5.	Excluded; no control group
931	Thakur VK, Londhe SM, Kumar P, Sharma M, Jain A, Pradhan I. Evaluation and quantification of airway changes in Class II division 1 patients undergoing myofunctional therapy using twin block appliance. Medical Journal Armed Forces India. 2020.	Excluded; no control group
932	Thereza-Bussolaro C, Oh HS, Lagravère M, Flores-Mir C. Pharyngeal dimensional changes in class II malocclusion treatment when using Forsus® or intermaxillary elastics - An exploratory study. Int Orthod. 2019;17(4):667-77.	Excluded; no control group
933	Verma G, Tandon P, Nagar A, Singh GP, Singh A. Cephalometric evaluation of hyoid bone position and pharyngeal spaces following treatment with Twin block appliance. J Orthod Sci. 2012;1(3):77-82.	Excluded; no control group
934	Vinoth SK, Thomas AV, Nethravathy R. Cephalomteric changes in airway dimensions with twin block therapy in growing Class II patients. J Pharm Bioallied Sci. 2013;5(Suppl 1):S25-9.	Excluded; no control group
935	Yassaei S, Tabatabaei Z, Ghafurifard R. Stability of pharyngeal airway dimensions: tongue and hyoid changes after treatment with a functional appliance. Int J Orthod Milwaukee. 2012;23(1):9-15.	Excluded; no control group
936	Zhang C, He H, Ngan P. Effects of twin block appliance on obstructive sleep apnea in children: a preliminary study. Sleep Breath. 2013;17(4):1309-14.	Excluded; no control group
937	Abdalla Y, Brown L, Sonnesen L. Effects of a fixed functional appliance on upper airway volume: A 3-dimensional cone-beam computed tomography study. Am J Orthod Dentofacial Orthop. 2020;158(1):40-9.	Excluded; control group not Class II
938	Han S, Choi YJ, Chung CJ, Kim JY, Kim KH. Long-term pharyngeal airway changes after bionator treatment in adolescents with skeletal Class II malocclusions. Korean J Orthod. 2014;44(1):13-9.	Excluded; control group not Class II

939	Iwasaki T, Sato H, Suga H, Minami A, Yamamoto Y, Takemoto Y, et al. Herbst appliance effects on pharyngeal airway ventilation evaluated using computational fluid dynamics. Angle Orthod. 2017;87(3):397-403.	Excluded; control group not Class II
940	Iwasaki T, Takemoto Y, Inada E, Sato H, Saitoh I, Kakuno E, et al. Three-dimensional cone-beam computed tomography analysis of enlargement of the pharyngeal airway by the Herbst appliance. Am J Orthod Dentofacial Orthop. 2014;146(6):776-85.	Excluded; control group not Class II
941	Li L, Liu H, Cheng H, Han Y, Wang C, Chen Y, et al. CBCT evaluation of the upper airway morphological changes in growing patients of class II division 1 malocclusion with mandibular retrusion using twin block appliance: a comparative research. PLoS One. 2014;9(4):e94378.	Excluded; control group cross-sectional
942	Li L, Wu W, Yan G, Liu L, Liu H, Li G, et al. Analogue simulation of pharyngeal airflow response to Twin Block treatment in growing patients with Class II(1) and mandibular retrognathia. Sci Rep. 2016;6:26012.	Excluded; control group cross-sectional
943	Aras I, Pasaoglu A, Olmez S, Unal I, Aras A. Upper airway changes following single-step or stepwise advancement using the Functional Mandibular Advancer. J Orofac Orthop. 2016;77(6):454-62.	Excluded; control group receiving treatment
944	Hanggi MP, Teuscher UM, Roos M, Peltomaki TA. Long-term changes in pharyngeal airway dimensions following activator-headgear and fixed appliance treatment. Eur J Orthod. 2008;30(6):598-605.	Excluded; control group receiving treatment
945	E HW. Pharyngeal airway increases with LARS treatment for retrognathia. Cranio. 2020;38(1):30-3.	Excluded; inadequate data
946	Gu M, Lin Y, McGrath CPJ, Hagg U, Wong RWK, Yang Y. Evaluation of the upper airway dimensions following Herbst appliance treatment in adolescents: A retrospective study. Apos Trends in Orthodontics. 2020;10(3):153-63.	Excluded; inadequate data
947	H Williamson E. Pharyngeal airway increases with LARS treatment for retrognathia. Cranio. 2018 May 31:1-4.	Excluded; inadequate data
948	Aksu M, Gorucu-Coskuner H, Taner T. Assessment of upper airway size after orthopedic treatment for maxillary protrusion or mandibular retrusion. Am J Orthod Dentofacial Orthop. 2017;152(3):364-70.	Included
949	Alhammadi MS, Elfeky HY, Fayed MS, Ishaq RAR, Halboub E, Al-Mashraqi AA. Three-dimensional skeletal and pharyngeal airway changes following therapy with functional appliances in growing skeletal Class II malocclusion patients : A controlled clinical trial. J Orofac Orthop. 2019 Sep;80(5):254-265.	Included
950	Ali B, Shaikh A, Fida M. Effect of Clark's twin-block appliance (CTB) and non-extraction fixed mechano-therapy on the pharyngeal dimensions of growing children. Dental Press J Orthod. 2015;20(6):82-8.	Included
951	Atik E, Gorucu-Coskuner H, Kocadereli I. Dentoskeletal and airway effects of the X-Bow appliance versus removable functional appliances (Frankel-2 and Trainer) in prepubertal Class II division 1 malocclusion patients. Australian Orthodontic Journal. 2017;33(1):3-13.	Included
952	Bavbek NC, Tuncer BB, Turkoz C, Ulusoy C, Tuncer C. Changes in airway dimensions and hyoid bone position following class II correction with forsus fatigue resistant device. Clin Oral Investig. 2016;20(7):1747-55.	Included
953	Cortese M, Pigato G, Casiraghi G, Ferrari M, Bianco E, Maddalone M. Evaluation of the Oropharyngeal Airway Space in Class II Malocclusion Treated with Mandibular Activator: A Retrospective Study. J Contemp Dent Pract. 2020;21(6):666-72.	Included
954	Drosen C, Bock NC, von Bremen J, Pancherz H, Ruf S. Long-term effects of Class II Herbst treatment on the pharyngeal airway width. Eur J Orthod. 2018;40(1):82-9.	Included
955	Elfeky HY, Fayed MMS. Three-dimensional effects of twin block therapy on pharyngeal airway parameters in Class II malocclusion patients. Journal of the World Federation of Orthodontists. 2015;4(3):114-9.	Included
956	Entrenas I, González-Chamorro E, Álvarez-Abad C, Muriel J, Menéndez-Díaz I, Cobo T. Evaluation of changes in the upper airway after Twin Block treatment in patients with Class II malocclusion. Clin Exp Dent Res. 2019;5:259–268.	Included
957	Fabiani G, Galvan Galvan J, Raucci G, Elyasi M, Pacheco-Pereira C, Flores-Mir C, et al. Pharyngeal airway changes in pre-pubertal children with Class II malocclusion after Frankel-2 treatment. Eur J Paediatr Dent. 2017;18(4):291-5.	Included
958	Ghodke S, Utreja AK, Singh SP, Jena AK. Effects of twin-block appliance on the anatomy of pharyngeal airway passage (PAP) in class II malocclusion subjects. Prog Orthod. 2014;15:68.	Included
959	Göymen M, Mourad D, Güleç A. Evaluation of Airway Measurements in Class II Patients Following Functional Treatment. Turk J Orthod. 2019 Mar;32(1):6-10.	Included
960	Jena AK, Singh SP, Utreja AK. Effectiveness of twin-block and Mandibular Protraction Appliance-IV in the improvement of pharyngeal airway passage dimensions in Class II malocclusion subjects with a retrognathic mandible. Angle Orthod. 2013;83(4):728-34.	Included
961	Kilinc DD, Sayar G. Pharyngeal airway changes of patients after Class II activator treatment. Selcuk Dent J 2018;5:8-12.	Included
962	Oliveira PM, Cheib-Vilefort PL, de Pársia Gontijo H, Melgaço CA, Franchi L, McNamara JA, Jr., et al. Three-dimensional changes of the upper airway in patients with Class II malocclusion treated with the Herbst appliance: A cone-beam computed tomography study. Am J Orthod Dentofacial Orthop. 2020;157(2):205-11.	Included
963	Ozbek MM, Memikoglu TU, Gogen H, Lowe AA, Baspinar E. Oropharyngeal airway dimensions and functional-orthopedic treatment in skeletal Class II cases. Angle Orthod. 1998;68(4):327-36.	Included
964	Pavoni C, Cretella Lombardo E, Franchi L, Lione R, Cozza P. Treatment and post-treatment effects of functional therapy on the sagittal pharyngeal dimensions in Class II subjects. Int J Pediatr Otorhinolaryngol. 2017;101:47-50.	Included
965	Rizk S, Kulbersh VP, Al-Qawasmi R. Changes in the oropharyngeal airway of Class II patients treated with the mandibular anterior repositioning appliance. Angle Orthod. 2016;86(6):955-61.	Included
966	Rongo R, Martina S, Bucci R, Festa P, Galeotti A, Alessandri Bonetti G, et al. Short-term effects of the Sander bite-jumping appliance on the pharyngeal airways in subjects with skeletal Class II malocclusion: A retrospective case-control study. Journal of Oral Rehabilitation. 2020.	Included
967	Ulusoy C, Canigur Bavbek N, Tuncer BB, Tuncer C, Turkoz C, Gencturk Z. Evaluation of airway dimensions and changes in hyoid bone position following class II functional therapy with activator. Acta Odontol Scand. 2014;72(8):917-25.	Included

Table S3. Malocclusion characteristics of the patients within the included studies.

Study	Malocclusion
Aksu 2017 [29]	ANB>5º; Class II molar relationship; no respiratory problems
Alhammadi 2019 [30]	Overjet≥5mm; ≥½ Class II molar relationship
Ali 2015 [31]	ANB>4º; SNB<78º; SN-ML 28-36º; bilateral Class II molar relationship
Atik 2017 [32]	Class II molar relationship; Class II/1; SNB<78º; overjet≥4mm
Bavbek 2016 [33]	ANB>4º; Class II/1; SNB<80º; overjet>5mm; SN-ML 26-38º; no respiratory problems
Cortese 2020 [34]	ANB>4º; overjet >4mm; Class II/1; retrognathic mandible
Drosen 2018 [35]	ANB>4º; Class II molar relationship (for the EG)
Elfeky 2015 [36]	ANB>4º; overjet>4mm; Class II molar relationship; no respiratory problems
Entrenas 2019 [37]	Mandibular Class II
Fabiani 2017 [38]	Class II/1; overjet>4mm; ANB>4º; SNB<78º; bilateral Cl. ½-full II molar relationship; cervical vertebrae maturation stage 1-2; good compliance (for the experimental group)
Ghodke 2014 [39]	Bilateral Class II molar relationship; SNA 79-84º; SNB≤º76; overjet=6-10mm; FMA 20-28º
Goymen 2019 [40]	Class II/1; ANB>4º; overjet>5mm
Jena 2013 [41]	Class II/1; bilateral Class II molar relationship; overjet=6-10mm; FMA 20-25º; no proclined anterior teeth
Kilinc 2018 [42]	Class II molar relationship; retrognathic mandible; during growth spurt
Oliveira 2020 [43]	Class II; ANB>4º; retrognathic mandible; cervical vertebrae maturation stage 2-5
Ozbek 1998 [44]	Class II molar relationship, ANB>4º; retrognathic mandible; overjet>5mm; no respiratory problems; hand-wrist radiograph $<MP3_{cap}$
Pavoni 2017 [45]	½ or full Class II molar relationship; ANB≥4º; overjet>5mm; normo-/hypo-divergent; cervical vertebrae maturation stage 2-3; sleep disordered breathing (for the experimental group)
Rizk 2016 [46]	Class II molar relationship; ANB≥4.5º; SNB ≤77º; SN-ML≥27º; cervical vertebrae maturation stage<5; no respiratory problems
Rongo 2020 [47]	Full Class II molar relationship; overjet>6mm; retrognathic mandible; cervical vertebrae maturation stage 2-3; SN-ML 26-38º
Ulusoy 2014 [48]	ANB>5º; SNB<80º; overjet>5mm; SN-ML 26-38º; no respiratory problems

Table S4. Detailed risk of bias of included non-randomized studies.

Reference	Aksu 2017	Alhammadi 2019	Ali 2015	Atik 2017	Bavbek 2016	Cortese 2020	Drosen 2018	Elfeky 2015	Entrenas 2019	Fabiani 2017	Ghodke 2014
Was the study prospective?	N	Y	N	N	N	N	N	Y	Y	N	Y
Was selection of patients based on any factor that could influence the outcome (malocclusion, airways, compliance, missed appointments, breakages)?	PN	N	PY	N	N	N	PN	N	PN	PY	PN
Were FA/CTR groups clearly defined?	PY	Y	Y	PY	Y	Y	PN	Y	PY	Y	Y
Were FA/CTR patients treated/observed at the same place/time?	Y	Y	N	NI	PY	NI	N	Y	PY	PY	PY
Were FA/CTR patients matched for baseline age?	Y	PN	Y	N	PN	Y	Y	PY	PY	PY	NI
Were FA/CTR patients matched for baseline sex?	PN	Y	Y	PY	PY	Y	Y	Y	Y	PN	PY
Were FA/CTR patients matched for baseline malocclusion?	PY	PY	PN	Y	PY	Y	Y	PY	NI	PN	PY
Were FA/CTR patients matched for baseline airway measurements?	PY	N	N	N	PY	PY	Y	PY	N	N	PY
Was the use of other appliances the same among FA/CTR patients?	NA	NA	N	NA	PY	NA	PN	NA	NA	NA	PN
Was the observation period similar for FA/CTR patients?	PY	NI	Y	N	N	N	PN	Y	NI	PY	Y
Were FA/CTR patients measured exactly the same way?	Y	Y	Y	Y	Y	Y	Y	Y	Y	Y	Y
Were FA/CTR patients measured blindly?	N	N	N	N	N	N	N	N	N	N	N
Was the adequate sample? (25 patients per group)	N	N	Y	N	N	N	N	N	Y	N	N

CTR, untreated control group; FA, fixed appliance group; N, no; NA, not applicable; NI, no information; PN, probably no; PY, probably yes; Y, yes.

Table S4. Detailed risk of bias of included non-randomized studies (*continued*).

Reference	Goymen 2019	Jena 2013	Kilinc 2018	Oliveira 2020	Ozbek 1998	Pavoni 2017	Rizk 2016	Rongo 2020	Ulusoy 2014
Was the study prospective?	N	N	N	N	N	Y	N	N	N
Was selection of patients based on any factor that could influence duration (malocclusion, airways, compliance, missed appointments, breakages)?	N	N	PN	PN	N	PY	PN	PN	PN
Were FA/CTR groups clearly defined?	Y	Y	PY	Y	PY	PY	PY	PY	PY
Were FA/CTR patients treated/observed at the same place/time?	PY	PY	NI	PY	PN	NI	NI	PN	NI
Were FA/CTR patients matched for baseline age?	N	N	NI	NI	PY	PY	NI	PY	PY
Were FA/CTR patients matched for baseline sex?	NI	PN	PN	PY	PY	PY	NI	PY	PN
Were FA/CTR patients matched for baseline malocclusion?	N	PN	N	NI	PY	PY	NI	PY	PY
Were FA/CTR patients matched for baseline airway measurements?	N	PN	N	PN	PN	PN	PN	PN	PY
Was the use of other appliances the same among FA/CTR patients?	NA	PN	NA	N	PN	NA	NA	NA	NA
Was the observation period similar for FA/CTR patients?	NI	PN	Y	PN	N	PY	NI	PY	PY
Were FA/CTR patients measured exactly the same way?	Y	Y	Y	Y	Y	PY	PY	Y	PY
Were FA/CTR patients measured blindly?	N	N	N	N	N	N	N	N	N
Was the adequate sample? (25 patients per group)	N	N	N	N	N	Y	N	Y	N

CTR, untreated control group; FA, fixed appliance group; N, no; NA, not applicable; NI, no information; PN, probably no; PY, probably yes; Y, yes.

Table S5. Results of individual studies not included in meta-analyses.

Study	Appliance	Outcome*	MD (95% CI)	P	CR
Aksu 2017	Activator	Epiglottic airway space (mm)	0.90 (-0.65, 2.45)	0.26	-
Fabiani 2017	Fränkel-2	PNS-H (mm)	0.15 (-0.88, 1.18)	0.78	-
Fabiani 2017	Fränkel-2	Ptm-Ba (mm)	-0.34 (-1.27, 0.59)	0.47	-
Goymen 2019	Twin-Block / FFRD	Hypopharynx dimension (mm)	-0.61 (-0.86, -0.37)	<0.001	No
Goymen 2019	Twin-Block / FFRD	Nasopharynx dimension (mm)	0.66 (0.35, 0.97)	<0.001	Yes
Oliveira 2020	Herbst	Nasal cavity volume (mm^3)	-1513.75 (-4033.94, 1006.44)	0.24	-
Pavoni 2017	Activator	Phw1-Psp (mm)	4.50 (3.88, 5.13)	<0.001	Yes
Pavoni 2017	Activator	lin_U-PNS (mm)	1.60 (1.01, 2.19)	<0.001	No
Rizk 2016	MARA	Oropharynx cross-section (mm)	3.99 (1.98, 6.00)	<0.001	No
Rongo 2020	Sander	Pharynx dimension (on the B-Go line; mm)	0.60 (-1.84, 3.04)	0.63	-
Ulusoy 2014	Activator	Nasopharynx area (mm^2)	160.00 (-371.76, 691.76)	0.56	-
Ulusoy 2014	Activator	Pharynx dimension (at the velum; mm)	0.25 (-1.57, 2.07)	0.79	-

* from explanation of each landmark, consult the original studies.

CI, confidence interval; CR, clinically relevant (judged as effect being larger than one standard deviation of the control group pre-treatment); FFRD, Forsus Fatigue Resistant Device; MARA, Mandibular Anterior Repositioning Appliance; MD, mean difference.

Figure S1. Contour-enhanced funnel plots for assessing reporting biases and publication bias.

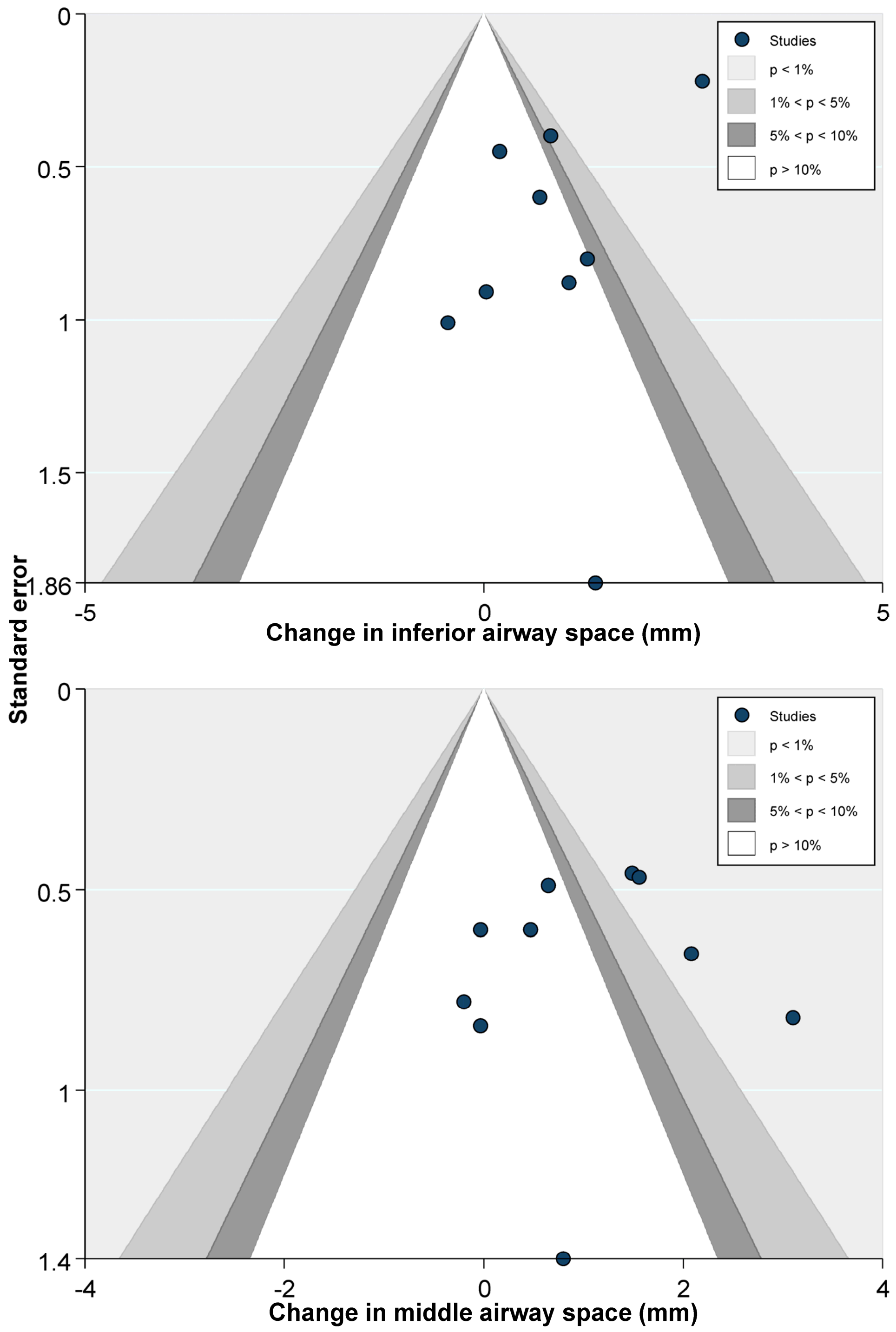

Supplement: Supplementary file 1 [file jcm-09-03806-s001.pdf]
